# On the boundary Carrollian conformal algebra

**DOI:** 10.1007/s11005-026-02118-z

**Published:** 2026-07-16

**Authors:** Lucas Buzaglo, Xiao He, Tuan Anh Pham, Haijun Tan, Girish S. Vishwa, Kaiming Zhao

**Affiliations:** 1 Department of Mathematics, UC San Diego, La Jolla, CA 92093-0112 USA; 2 Paris Curie Engineer School, Beijing University of Chemical Technology, Beijing, 100029 People’s Republic of China; 3 Maxwell Institute and School of Mathematics, The University of Edinburgh, James Clerk Maxwell Building, Peter Guthrie Tait Road, Edinburgh, EH9 3FD Scotland, UK; 4 School of Mathematics and Statistics, Northeast Normal University, Changchun, 130024 Jilin People’s Republic of China; 5 Department of Mathematics, Wilfrid Laurier University, Waterloo, ON N2L 3C5 Canada

**Keywords:** Boundary Carrollian conformal field theory, BCCA, Tensionless open string, Witt algebra, Virasoro algebra, BMS algebra, Whittaker module, Primary 17B68, 17B10, Secondary 17B81, 17B65

## Abstract

We initiate the mathematical study of the boundary Carrollian conformal algebra (BCCA), an infinite-dimensional Lie algebra recently discovered in the context of Carrollian physics. The BCCA is an intriguing object from both physical and mathematical perspectives, since it is a filtered but not graded Lie algebra. In this paper, we first construct some modules for the BCCA and one of its subalgebras, which we call $\mathcal {O}$, by restriction of well-known modules of the BMS_3_ and Witt algebras, respectively. Along the way, we prove the irreducibility criteria for the so-called induced modules of the BMS_3_ algebra (which we prefer to call massive modules to avoid ambiguity) and show that this is the same criteria for the irreducibility of the Verma modules of the BMS_3_ algebra. Interestingly, the modules generated by the action of the BCCA on the generating vector of the massive modules are also irreducible under the same criteria. When these criteria hold, every massive module decomposes into a direct sum of two BCCA-submodules, each of which we conjecture to be indecomposable. Meanwhile, restricting Verma modules to the BCCA and $\mathcal {O}$ leads to free or “almost-free” modules, which are not particularly interesting from a representation-theoretic viewpoint. This motivates the construction of BCCA modules intrinsically. To do this, we go through some structure theory on the BCCA to define a new basis and a decreasing filtration on the algebra, using which we construct Whittaker modules over the BCCA and the subalgebra $\mathcal {O}$ and prove criteria for their irreducibility.

## Introduction

Over the past two decades, research on infinite-dimensional Lie algebras has enjoyed continuous motivation and inspiration from theoretical physics. Indeed, with the discovery of more examples of infinite-dimensional Lie algebras in physical contexts comes the need to better understand these algebras and their representations. On many occasions, this leads to a productive cycle of progress in both fields, due to the resulting mathematics research informing physics in some manner, which once again motivates another mathematical study. The origin of this paper is no different—we present a first study on another infinite-dimensional Lie algebra that was recently discovered in physics, known as the boundary Carrollian conformal algebra (BCCA), which we denote by $\widehat{\mathfrak {b}}$. Its centreless version is denoted by $\mathfrak {b}$.

The BCCA was discovered as the symmetry algebra of a Carrollian conformal field theory (CCFT) with boundary in [12], in which it is formulated as a subalgebra of the BMS_3_ algebra (realised as operators in a quantum field theory) that preserves a choice of boundary on a CCFT on a cylinder. Its original basis consists of the elements $\mathcal {O}_n :=L_n - L_{-n}$ (n≥1), $P_n :=M_n + M_{-n}$ (n≥0), and $C_M$, with its Lie bracket given by

1.1 $$\begin{aligned} \begin{gathered} {[}\mathcal {O}_n,\mathcal {O}_m] = (n - m)\mathcal {O}_{n + m} - (n + m)\mathcal {O}_{n - m}, \\ {[}\mathcal {O}_n, P_m] = (n - m)P_{n + m} + (n + m)P_{n - m} + \frac{1}{6}n(n^2 - 1) \delta _{m,n} C_M, \\ {[}P_n,P_m] = 0, \end{gathered} \end{aligned}$$

where $L_n$, $M_n$, and $C_M$ are elements of the BMS_3_ algebra defined in Definition 2.3.

In fact, the authors of [12] showed that there exist at least three different boundary-preserving subalgebras of BMS_3_, but they choose to work with the choice given by (1.1) since it emerges as the algebra of symmetries on the worldsheet of the tensionless open strings, as they demonstrate in their paper. This serves as the primary motivation for our work: understanding the representations of the symmetry algebra of a string theory worldsheet can provide insights into the spectrum of the string theory. Nonetheless, it is interesting to note that one of the other two choices was also discovered in [3] as the worldsheet symmetry algebra of *“topological” open strings*. This finding presents a further case for the relevance of our work: there may exist connections between the methods developed in this paper and tropical geometry, which in turn could inform string theory.

There are two important features that the BCCA lacks that makes its study both challenging and warranted, these being a grading and a Virasoro subalgebra. Symmetry algebras of string theory worldsheets usually have both, making them amenable to thorough, quantum mechanical treatment using the power tools of vertex operator algebras and semi-infinite cohomology [24, 25, 43, 44, 64]. Today, these toolkits can be coupled with the troves of knowledge on the representation theory of integer-graded infinite-dimensional Lie algebras, to which the past two decades of active research in both physics [1, 13–18, 34, 46, 47, 53] and mathematics [4, 6, 19, 20, 39, 50, 56, 57, 61, 62, 65, 67, 69, 73, 84] have contributed. However, with neither a grading nor a Virasoro subalgebra, the BCCA cannot be studied using these well-established techniques. Hence, understanding the representations of the BCCA presents a novel challenge for both physicists and mathematicians. We therefore develop and employ new approaches that we hope can lay the groundwork for future studies on infinite-dimensional Lie algebras that are not integer-graded while shedding some light on the spectrum of the open tensionless string.

Since the BCCA is defined as a subalgebra of the BMS_3_ algebra, it is natural to ask whether irreducible modules over BMS_3_ remain irreducible upon restriction to the BCCA. We therefore start by studying representations of the BCCA and its subalgebra $\mathcal {O}:={{\,\textrm{span}\,}}\{\mathcal {O}_n \mid n \ge 1\}$ by restricting well-known representations of the Virasoro and BMS_3_ algebras, namely the tensor density modules, Verma modules, the so-called induced modules introduced in [22] which we prefer to call “massive modules” (see [34]), and the modules $\Omega (\lambda ,a)$ introduced in [66]. We work over an arbitrary, characteristic-zero field k to prove the following.

### Theorem 1.1

(Lemma 2.7, Propositions 3.3, 3.5, 3.11, and 3.18) Let $h_L, h_M, c_L, c_M, a, b, \textbf{M}, \textbf{s} \in \Bbbk$, and $\lambda \in \Bbbk ^*$. Then the following hold.

1. The tensor density module *I*(0, *b*) (see Definition 2.2) decomposes into a direct sum of two $\mathcal {O}$-submodules.
2. When restricted to $\mathcal {O}$, the Virasoro Verma module $V(h_L,c_L)$ (see Definition 3.1) is a free module of rank 1. That is, $V(h_L,c_L) \cong U(\mathcal {O})$ as $\mathcal {O}$-modules.
3. When restricted to the BCCA, the BMS_3_ Verma module $V(h_L, h_M, c_L, c_M)$ (see Definition 3.4) has the following form:
  $$\begin{aligned} V(h_L, h_M, c_L, c_M) \cong \frac{U(\widehat{\mathfrak {b}})}{U(\widehat{\mathfrak {b}})\cdot (P_0 - 2h_M, C_M - c_M)}. \end{aligned}$$
4. The BCCA module $U(\widehat{\mathfrak {b}})|\textbf{M}, \textbf{s}\rangle$ (see Definition 3.6) is irreducible if and only if $\textbf{M} + \frac{n^2 - 1}{24}c_M \ne 0$ for any positive integer *n*.
5. The $\mathcal {O}$-module $\Omega (\lambda ,a)$ (see Sect. 3.4) is irreducible if and only if $\lambda \ne \pm 1$ and a≠0.

Taking Theorem 1.1(4) a bit further, we showed that the massive module $\widetilde{V}(\textbf{M},\textbf{s},c_L,c_M)$ is decomposable as a $\widehat{\mathfrak {b}}$-module when $U(\widehat{\mathfrak {b}}) |\textbf{M}, \textbf{s}\rangle$ is irreducible (i.e. $\textbf{M} + \frac{n^2 - 1}{24}c_M \ne 0$ for any positive integer *n*).

### Theorem 1.2

(Propositions 3.13 and 3.15, Corollary 3.16) Let $\textbf{M} + \frac{n^2 - 1}{24}c_M \ne 0$ for any positive integer *n*. Then, the massive module $\widetilde{V}(\textbf{M},\textbf{s},c_L,c_M)$ decomposes into a direct sum of two $\widehat{\mathfrak {b}}$-submodules:

$$\begin{aligned} \widetilde{V}(\textbf{M},\textbf{s},c_L ,c_M) \cong \widehat{V} \oplus \widehat{W}, \end{aligned}$$

where $\widehat{V}$ and $\widehat{W}$ are given by Notation 3.12 and Definition 3.14.

Theorems 1.1 and 1.2 show that many standard examples of BMS_3_ modules are no longer irreducible upon restriction to the BCCA. In fact, we even see that the tensor density modules and the massive $\mathfrak {bms}$-modules become decomposable when we restrict to the BCCA. On the other hand, Theorem 1.1(4) shows that the massive modules do contain irreducible BCCA-submodules.

We then present the following conjecture as an avenue for further study.

### Conjecture 1.3

(Conjecture 3.17) The $\widehat{\mathfrak {b}}$-modules $\widehat{V}$ and $\widehat{W}$ are indecomposable.

On the way to proving Theorem 1.1(4), we also characterise when the massive modules over the BMS_3_ algebra^1^ are irreducible, which to our knowledge was not known before.

### Theorem 1.4

(Theorem 3.7) Let $\textbf{M},\textbf{s},c_L,c_M \in \Bbbk$. Then, the massive module $\widetilde{V}(\textbf{M},\textbf{s},c_L,c_M)$ over the BMS_3_ algebra (see Definition 3.6) is irreducible if and only if $\textbf{M} + \frac{n^2 - 1}{24}c_M \ne 0$ for any positive integer *n*.

This is in line with what is expected based on the gravitational interpretation of the coadjoint orbits of the BMS_3_ group (see [23] for more details). However, it is surprising that the irreducibility conditions for the massive modules over $\mathfrak {bms}$ and the $\widehat{\mathfrak {b}}$-module $U(\widehat{\mathfrak {b}})|\textbf{M}, \textbf{s}\rangle$ are the same.

Theorem 1.1 shows that many of the well-known modules over the Virasoro and BMS_3_ algebras become free, or “almost free” when restricted to the BCCA. From the perspective of representation theory, these are not the most interesting BCCA modules, so we later shift our focus to studying the representation theory of the BCCA intrinsically, by way of Whittaker modules (see Definition 2.9). The main issue is that the presentation of the BCCA given in (1.1) makes it difficult to even define what a Whittaker module over the BCCA should be. For this reason, we spend some time constructing a different basis of the (centreless) BCCA, which better lends itself to the construction of Whittaker modules.

The change of basis is achieved by analysing the subalgebra $\mathcal {O}$, which is a subalgebra of the Witt algebra $\mathcal {W}:={{\,\textrm{Der}\,}}(\Bbbk [t,t^{-1}])$, using techniques from [7, 29]. In fact, we work in a more general setting: for $\lambda \in \Bbbk ^*$ and $n \in \mathbb {Z}$, let $\mathcal {O}^{(\lambda )}_n :=L_n - \lambda ^n L_{-n}$, and define $\mathcal {O}(\lambda ) :={{\,\textrm{span}\,}}\{\mathcal {O}^{(\lambda )}_n \mid n \ge 1\}$. It is easy to see that $\mathcal {O}(\lambda )$ is a Lie algebra with bracket given by

$$\begin{aligned} {[}\mathcal {O}^{(\lambda )}_n,\mathcal {O}^{(\lambda )}_m] = (n - m)\mathcal {O}^{(\lambda )}_{n + m} - \lambda ^m (n + m) \mathcal {O}^{(\lambda )}_{n - m} \end{aligned}$$

for all $n,m \in \mathbb {Z}_+$ (see Lemma 4.14).

### Theorem 1.5

(Theorem 4.16, Proposition 4.22, and Corollary 4.23) Let $\lambda , \mu \in \Bbbk ^*$. Then, $\mathcal {O}(\lambda )$ has another basis $\{u^{(\lambda )}_n \mid n \ge 1\}$ with Lie bracket

$$\begin{aligned} {[}u^{(\lambda )}_n, u^{(\lambda )}_m] = (n - m)(u^{(\lambda )}_{n + m} - 4\lambda u^{(\lambda )}_{n + m - 2}). \end{aligned}$$

Consequently, $\mathcal {O}(\lambda ) \cong \mathcal {O}(\mu )$ if and only if $\sqrt{\frac{\lambda }{\mu }} \in \Bbbk$. In particular, if k is algebraically closed (for example, if $\Bbbk = \mathbb {C}$), then $\mathcal {O}(\lambda ) \cong \mathcal {O}$ for all $\lambda \in \Bbbk$. If $\Bbbk = \mathbb {R}$, then

$$\mathcal {O}(\lambda ) \cong {\left\{ \begin{array}{ll} \mathcal {O}, & {\text {if }} \lambda > 0, \\ \mathcal {O}(-1), & {\text {if }} \lambda < 0, \end{array}\right. }$$

but $$\mathcal {O}(-1) \not \cong \mathcal {O}$$.

We further present some consequences of Theorem 1.5 in Sect. 4. For example, it becomes easy to show that $\mathcal {O}$ is not a simple Lie algebra (in fact, it is not even perfect)—see Corollary 4.19. Certainly, the same is true for both $\mathfrak {b}$ and $\widehat{\mathfrak {b}}$.

Theorem 1.5 tells us that the Lie subalgebras $\mathcal {O}$ and $\mathcal {O}(-1)$ of the Witt algebra are isomorphic as complex Lie algebras but not as real ones. This leads to an interesting physical implication. In [12], the authors start with the BMS_3_ algebra realised as vector fields which generate the infinitesimal transformations that leave the Carrollian structure on a cylinder (given by a symmetric (0, 2)-tensor field *h* that is corank-1 everywhere on the cylinder and a nowhere-vanishing vector field ξ such that kerh is generated by ξ) invariant up to an overall conformal factor. Using this, the Lie algebra $\mathcal {O}$ was constructed as the subalgebra of the Witt algebra consisting of the vector fields that preserve a choice of boundaries on the cylinder at 0 and π. Likewise, the Lie algebra $\mathcal {O}(-1)$ can be viewed as the analogous Lie algebra when the boundaries are instead placed at $\tfrac{\pi }{2}$ and $\tfrac{3\pi }{2}$. But one can always recover the original boundary conditions by translating the periodic coordinate by $\tfrac{\pi }{2}$, which puts back in the setting where $\mathcal {O}$ is the distinguished boundary-preserving subalgebra. This equivalence must be reflected algebraically too, which indicates that $\mathcal {O}(-1)$ and $\mathcal {O}$ must be isomorphic Lie algebras. Thus, by Theorem 1.5, we confirm that the authors [12] must be working over $\mathbb {C}$ and not $\mathbb {R}$. We provide details of this observation in Remark 4.24.

Having constructed the alternate basis of $\mathcal {O}$ in Theorem 1.5, we then extend it to a new basis of the full centreless BCCA.

### Theorem 1.6

(Corollary 4.34) There is another basis of $\mathfrak {b}$ denoted $\{u_n, v_m \mid n \ge 1, m \ge 0\}$. The bracket of the centreless BCCA with this basis is given by

$$\begin{aligned} {[}u_n,u_m]&= (n - m)(u_{n + m} - 4u_{n + m - 2})  &   (n,m \ge 1), \\ {[}u_n,v_m]&= (n - m)v_{n + m} - 4(n - m - 1)v_{n + m - 2}  &   (n \ge 1, m \ge 0), \\ {[}v_n,v_m]&= 0  &   (n,m \ge 0). \end{aligned}$$

Using the new basis for $\mathfrak {b}$ from Theorem 1.6, we can easily construct a filtration for the centreless BCCA, allowing us to define and study Whittaker modules for $\mathfrak {b}$, since filtered Lie algebras always have Whittaker modules (see Lemma 2.11). For convenience, we shift the basis as follows: let $\mathcal {U}_n :=u_{n + 2}$ and $\mathcal {V}_n :=v_{n + 1}$ for n≥-1.

### Proposition 1.7

(Proposition 5.2) For n≥-1, define

$$\begin{aligned} \mathcal {F}_n\mathcal {O}:={{\,\textrm{span}\,}}\{\mathcal {U}_k \mid k \ge n\}, \quad \mathcal {F}_n\mathfrak {b}:={{\,\textrm{span}\,}}\{\mathcal {U}_k, \mathcal {V}_k \mid k \ge n\}. \end{aligned}$$

Then, $\mathcal {F}$ is a filtration of $\mathcal {O}$ and $\mathfrak {b}$.

Consequently, we define *Whittaker functions* on $\mathfrak {b}$ to be Lie algebra homomorphisms $\psi _n :\mathcal {F}_n \mathfrak {b}\rightarrow \Bbbk$ for some $n \in \mathbb {N}$. Given a Whittaker function $\psi _n :\mathcal {F}_n \mathfrak {b}\rightarrow \Bbbk$, we let $\Bbbk _{\psi _n} = \Bbbk 1_{\psi _n}$ be the one-dimensional $\mathcal {F}_n \mathfrak {b}$-module defined by $x \cdot 1_{\psi _n} = \psi _n(x) 1_{\psi _n}$ for $x \in \mathcal {F}_n \mathfrak {b}$. The *universal Whittaker module of type* $\psi _n$ is defined to be $M_{\psi _n} :={{\,\textrm{Ind}\,}}_{\mathcal {F}_n \mathfrak {b}}^\mathfrak {b}\Bbbk _{\psi _n}$. Similar definitions can be made for the subalgebra $\mathcal {O}$.

### Theorem 1.8

(Theorems 6.6 and 6.28) Let $n \in \mathbb {N}$, and let $\varphi _n :\mathcal {F}_n \mathcal {O}\rightarrow \Bbbk$ and $\psi _n :\mathcal {F}_n \mathfrak {b}\rightarrow \Bbbk$ be Whittaker functions. Then the following hold.

1. If n=0, then the $\mathcal {O}$-module $M_{\varphi _0}$ is irreducible if and only if $\varphi _0(\mathcal {U}_{2}) \ne 4 \varphi _0(\mathcal {U}_{0})$.
2. If n≥1, then the $\mathcal {O}$-module $M_{\varphi _n}$ is irreducible if and only if $\varphi _n(\mathcal {U}_{2n + 1}) \ne 4 \varphi _n(\mathcal {U}_{2n - 1})$ or $\varphi _n(\mathcal {U}_{2n + 2}) \ne 4\varphi _n(\mathcal {U}_{2n})$.
3. If n=0, then the $\mathfrak {b}$-module $M_{\psi _0}$ is irreducible if and only if $\psi _0(\mathcal {V}_0) \ne 0$.
4. If n≥1, then the $\mathfrak {b}$-module $M_{\psi _n}$ is irreducible if and only if $\psi _n(\mathcal {V}_{2n}) \ne 0$ or $\psi _n(\mathcal {V}_{2n - 1}) \ne 0$.

The proof of Theorem 1.8(1) and (2) uses a version of the classical Kirillov orbit method [58] for the Virasoro algebra developed in [71, 81]. For parts (3) and (4), our proof is more direct.

Unfortunately, there is no way to extend the filtration to $\widehat{\mathfrak {b}}$, at least not while keeping the nice properties of the filtration (particularly *weak convergence*—see Definition 2.10). We elaborate on this in Remark 4.37. This means that we are currently unable to define Whittaker modules for the BCCA on which the centre acts nontrivially. It would be interesting to further explore representations of the BCCA on which the centre acts nontrivially.

Certainly, there exists strong physical and mathematical motivation for this paper. The former arises from the fact that an infinite-dimensional Lie algebra which is not integer-graded has made an appearance in a modern theoretical physics setting, meaning that we need to develop the tools needed to study such objects thoroughly, starting with the BCCA as the inaugural case study. The latter comes from the desire to understand infinite-dimensional subalgebras of infinite-dimensional Lie algebras more thoroughly.

This paper is organised as follows: In Sect. 2, we set up notation, present key definitions along with minor or known results, and introduce the main objects of study of this paper. Section 3 constructs modules of the BCCA and one of its subalgebras by restricting well-known modules of the BMS_3_ and Virasoro algebras. This would be the naive approach to the studying the representations of the BCCA based on its physical origins in [12]. The emergence of free and “almost-free” modules motivates the need to study modules of the BCCA intrinsically. This requires the change of basis presented in Sect. 4, from which we can construct a decreasing filtration of the BCCA and compute derived subalgebras, as done in Sect. 5. All this effort pays off in Sect. 6, in which we construct the universal Whittaker modules over the BCCA and its subalgebra $\mathcal {O}$ and determine criteria for their irreducibility. Finally, in Sect. 7, we present a myriad of future directions of work that can be undertaken on the representation theory of the BCCA, from both physical and mathematical perspectives.

## Preliminaries

Throughout this paper, we use the following notation:

- k denotes an arbitrary field of characteristic zero.
- $\Bbbk ^*$ denotes the set of nonzero elements of the field k.
- $\mathbb {N}$ denotes the set of non-negative integers (including zero).
- $\mathbb {Z}_+$ and $\mathbb {Z}_-$ denote the set of positive and negative integers, respectively.
- We reserve upper case *C* (and variations such as $C_L$ and $C_M$) for central elements of a Lie algebra, while lower case *c* (and variations such as $c_L$ and $c_M$) represents the scalars by which the central elements act on a given representation of the Lie algebra.

All Lie algebras and vector spaces are considered over k unless otherwise specified. We will also abbreviate $\otimes _\Bbbk$ to ⊗.

### The boundary Carrollian conformal algebra

We define the fundamental objects of interest in this paper and present the construction of the boundary Carrollian conformal algebra (BCCA).

#### Definition 2.1

The *Witt algebra* is defined as $\mathcal {W}:={{\,\textrm{Der}\,}}(\Bbbk [t,t^{-1}]) = \Bbbk [t,t^{-1}]\partial$, where $\partial :=\frac{d}{dt}$. The standard basis of the Witt algebra is $L_n :=-t^{n + 1}\partial$ ($n \in \mathbb {Z})$, and its Lie bracket is given by:

$$\begin{aligned} {[}f\partial , g\partial ] = (fg' - f'g)\partial , \qquad [L_n,L_m] = (n - m)L_{n + m}, \end{aligned}$$

for all $f,g \in \Bbbk [t,t^{-1}]$ and $n,m \in \mathbb {Z}$. The Witt algebra $\mathcal {W}$ can be seen as the Lie algebra of algebraic vector fields on $\Bbbk ^*$.

The *Virasoro algebra* ${{\,\textrm{Vir}\,}}$ is the universal central extension of the Witt algebra. As a vector space, ${{\,\textrm{Vir}\,}}= \mathcal {W}\oplus \Bbbk C$ with bracket given by

$$\begin{aligned}  &   {[}f\partial ,g\partial ] = (fg' - f'g)\partial + \frac{1}{12} \operatorname {Res}_0(f'g'')C, \\  &   {[}L_n,L_m] = (n - m)L_{n + m} + \frac{1}{12} n(n^2 - 1) \delta _{m,-n} C, \end{aligned}$$

for all $f,g \in \Bbbk [t,t^{-1}]$, where *C* is central, and $\operatorname {Res}_0(h)$ is the residue of $h \in \Bbbk [t,t^{-1}]$ at zero, or in other words, the coefficient of $t^{-1}$ in *h*.

The *one-sided Witt algebra* is defined as $\mathbb {W}_1 :={{\,\textrm{Der}\,}}(\Bbbk [t]) = \Bbbk [t]\partial$. Equivalently, it can be viewed as the subalgebra of $\mathcal {W}$ spanned by $\{L_n \mid n \ge -1\}$ and it is the Lie algebra of algebraic vector fields on k.

We often switch between the *basis-free* notation f∂ for elements of $\mathcal {W}$ and the basis notation $L_n$.

#### Definition 2.2

For $a,b \in \Bbbk$, define a $\mathcal {W}$-module $I(a,b) = {{\,\textrm{span}\,}}\{I_n \mid n \in \mathbb {Z}\}$ as follows:

$$\begin{aligned} L_n \cdot I_m = - (a + bn + m)I_{n + m}. \end{aligned}$$

The modules *I*(*a*, *b*) are known as *tensor density modules*. In basis-free notation, we define $I(a,b) :=t^{a - b}\Bbbk [t,t^{-1}] \ dt^b$ with $\mathcal {W}$-action given by

$$\begin{aligned} f\partial \cdot (g \ dt^b) = (fg' + bf'g)dt^b. \end{aligned}$$

With this perspective, we have $I_n :=- t^{n + a - b} \ dt^b$. Note that I(0,-1) is the adjoint module of $\mathcal {W}$.

We may then construct the two-parameter family of Lie algebras $\mathcal {W}(a,b) :=\mathcal {W}\ltimes I(a,b)$. The special case $\mathcal {W}(0,-1)$ is the centreless version of the BMS_3_ algebra, presented in the next definition. The BMS_3_ algebra first appeared in physics in [2, 10], and in mathematics, it was introduced as the *W*(2, 2) algebra^2^ by Zhang and Dong but with $C_L = C_M$ [84].

#### Definition 2.3

The *BMS*_3_ *algebra*, denoted $\mathfrak {bms}$, is the universal central extension of $\mathcal {W}(0,-1)$. It is the infinite-dimensional Lie algebra spanned by $\{L_n,M_n \mid n \in \mathbb {Z}\}$ and two central elements $C_L, C_M$, with Lie bracket

2.1 $$\begin{aligned} \begin{gathered} {[}L_n,L_m] = (n - m) L_{m + n} + \frac{1}{12} n(n^2 - 1) \delta _{m,-n} C_L, \\ {[}L_n,M_m] = (n - m) M_{m + n} + \frac{1}{12} n(n^2 - 1)\delta _{m,-n} C_M, \\ {[}M_n,M_m] = 0. \end{gathered} \end{aligned}$$

Here, $\{L_n \mid n\in \mathbb {Z}\}$ and $C_L$ form a basis of a Virasoro subalgebra and $\{M_n \mid n \in \mathbb {Z}\}$ is the basis of I(0,-1) presented in Definition 2.2.

We now define the BCCA in its original form given by [12, Equation (7)]. This will be the main object of study of our paper.

#### Definition 2.4

Let $\mathcal {O}_n:=L_n - L_{-n}$ and $P_n :=M_n + M_{-n}$ for all $n \in \mathbb {Z}$, which means that $\mathcal {O}_n = -\mathcal {O}_{-n}$ and $\mathcal {P}_n = \mathcal {P}_{-n}$ for all n≥1. The *boundary Carrollian conformal algebra (BCCA)*, denoted $\widehat{\mathfrak {b}}$, is the Lie subalgebra of $\mathfrak {bms}$ with basis $\{\mathcal {O}_n, P_m, C_M \mid n \ge 1,\ m \ge 0\}$ and Lie bracket

2.2 $$\begin{aligned} \begin{gathered} {[}\mathcal {O}_n,\mathcal {O}_m] = (n - m)\mathcal {O}_{n + m} - (n + m)\mathcal {O}_{n - m}, \\ {[}\mathcal {O}_n, P_m] = (n - m)P_{n + m} + (n + m)P_{n - m} + \frac{1}{6}n(n^2 - 1) \delta _{m,n} C_M, \\ {[}P_n,P_m] = 0. \end{gathered} \end{aligned}$$

The *centreless BCCA* (in other words, setting $C_M = 0$) is denoted $\mathfrak {b}$.

The latter half of this paper mostly focuses on the centreless BCCA. This is because it is not clear how one could define Whittaker modules over the BCCA with centre. We elaborate on this in Remark 4.37.

#### Remark 2.5

The Lie algebra $\mathfrak {b}$ is the fixed subalgebra of $\mathcal {W}(0,-1)$ under the automorphism $L_n \mapsto -L_{-n},\, M_n \mapsto M_{-n}$.

First, we seek to write $\mathfrak {b}$ as a semi-direct sum in a similar manner to how the centreless BMS_3_ algebra can be written as the semi-direct sum $\mathcal {W}(0,-1) :\!\!-\mathcal {W}\ltimes I(0,-1)$. To do this, we define the following subspaces of $\mathcal {W}$ and *I*(*a*, *b*).

#### Definition 2.6

The subalgebra $\mathcal {O}\subseteq \mathcal {W}$ is spanned by $\{\mathcal {O}_n \mid n \ge 1\}$, where $\mathcal {O}_n :=L_n - L_{-n}$. Its Lie bracket is given by

2.3 $$\begin{aligned} {[}\mathcal {O}_n, \mathcal {O}_m] = (n - m)\mathcal {O}_{n + m} - (n + m)\mathcal {O}_{n - m}. \end{aligned}$$

Let $I(0,b) = {{\,\textrm{span}\,}}\{I_n \mid n \in \mathbb {Z}\}$ be the tensor density modules from Definition 2.2. Let $P^{(b)}_n :=I_n + I_{-n}$ and construct $\mathcal {P}_b :={{\,\textrm{span}\,}}\{P^{(b)}_n \mid n \in \mathbb {N}\}$. Likewise, let $\widetilde{P}^{(b)}_n :=I_n - I_{-n}$ and construct $\widetilde{\mathcal {P}}_b :={{\,\textrm{span}\,}}\{\widetilde{P}^{(b)}_n \mid n \in \mathbb {N}^+\}$. The vector spaces $\mathcal {P}_b$ and $\widetilde{\mathcal {P}}_b$ are $\mathcal {O}$-modules under the following actions of $\mathcal {O}$ inherited from the action of $\mathcal {W}$ on *I*(0, *b*):

2.4 $$\begin{aligned}&\mathcal {O}_n \cdot P_m^{(b)} = -(bn + m) P_{n + m}^{(b)} - (bn - m) P_{n-m}^{(b)} \quad (n \ge 1, m \ge 0) \end{aligned}$$

2.5 $$\begin{aligned}&\mathcal {O}_n \cdot \widetilde{P}_m^{(b)} = -(bn + m) \widetilde{P}_{n + m}^{(b)} - (bn - m) \widetilde{P}_{n-m}^{(b)} \quad (n,m \ge 1). \end{aligned}$$

For b=-1, we write $\mathcal {P}:=\mathcal {P}_{-1}$. Note that $\widetilde{\mathcal {P}}_{-1}$ is the adjoint module of $\mathcal {O}$.

It is easy to see that *I*(0, *b*) decomposes as a direct sum of $\mathcal {P}_b$ and $\widetilde{\mathcal {P}}_b$ as an $\mathcal {O}$-module.

#### Lemma 2.7

As $$\mathcal {O}$$-modules, $$I(0,b)\cong \mathcal {P}_b \oplus \widetilde{\mathcal {P}}_b$$.

#### Proof

There exists an $\mathcal {O}$-module homomorphism $\rho :I(0,b)\rightarrow \mathcal {P}_b$ given by $\rho (I_n) = P_n$ for all $n\in \mathbb {Z}$. We then have a short exact sequence of $\mathcal {O}$-modules2.6The inclusion $\mathcal {P}_b \hookrightarrow I(0,b)$ is a section of the short exact sequence, and thus, the sequence is split. It follows that $I(0,b)\cong \mathcal {P}_b \oplus \widetilde{\mathcal {P}}_b$ for all $b\in \Bbbk$. □

We therefore see that Definition 2.4 can be succinctly summarised as $\mathfrak {b}:=\mathcal {O}\ltimes \mathcal {P}_{-1} = \mathcal {O}\ltimes \mathcal {P}$, analogous to how the centreless BMS_3_ algebra is defined as $\mathcal {W}(0,-1) :=\mathcal {W}\ltimes I(0,-1)$. The *higher spin (centreless) BCCAs* can be similarly defined as $\mathcal {O}\ltimes \mathcal {P}_b$ for $b \in \Bbbk$. See the commentary around [12, Equation (26)] for more details. We only focus on the case b=-1 in this paper, but our techniques could also be applied to these higher spin algebras.

### Whittaker modules

Whittaker modules were first discovered for $\mathfrak {sl}_2$ by Arnal and Pinczon in [5], following which Kostant defined them for finite-dimensional complex semisimple Lie algebras in [59]. This was then generalised to arbitrary Lie algebras with triangular decomposition. Today, Whittaker modules are defined more generally via the notion of “Whittaker pairs” [21, 69] and play an important role in the representation theory of various infinite-dimensional Lie algebras [4, 33, 35–37, 39, 51, 60, 63, 70, 82, 85].

In this paper, we construct Whittaker modules of $\mathcal {O}$ and $\mathfrak {b}$ based on the definition given in [21, Section 3.2] and [69, Section 5.1], thereby presenting explicit examples of their constructions for infinite-dimensional Lie algebras that are not integer-graded.

#### Definition 2.8

Let $\mathfrak {g}$ be a Lie algebra.

1. Define $\mathfrak {g}^0 :\!\!-\mathfrak {g}$ and $\mathfrak {g}^{k+1}:\!\!-[\mathfrak {g}^k, \mathfrak {g}]$ for all $k\in \mathbb {N}$. We say that $\mathfrak {g}$ is *quasi-nilpotent* if $\bigcap _{k=0}^\infty \mathfrak {g}^k = 0$.
2. A $\mathfrak {g}$-module *V* is said to be *locally nilpotent* if for every v∈V, there exists $s(v)\in \mathbb {N}$ such that $x_1 x_2 \dots x_{s(v)}\cdot v = 0$ for all $x_1,\dots , x_{s(v)} \in \mathfrak {g}$.

Equipped with the above definition, we can now define Whittaker modules.

#### Definition 2.9

Let $\mathfrak {g}$ be a Lie algebra with a quasi-nilpotent subalgebra $\mathfrak {n} \subseteq \mathfrak {g}$ such that $\mathfrak {g}/\mathfrak {n}$ is a locally nilpotent $\mathfrak {n}$-module under the adjoint action, and let *V* be a $\mathfrak {g}$-module.

1. The $\mathfrak {g}$-module *V* is a *Whittaker module* if $U(\mathfrak {n}) \cdot v$ is finite-dimensional for all v∈V (in other words, *V* is a *locally finite* $\mathfrak {n}$-module).
2. A Lie algebra homomorphism $\varphi :\mathfrak {n} \rightarrow \Bbbk$ is called a *Whittaker function* (so $\varphi ([\mathfrak {n}, \mathfrak {n}]) = 0$). A vector v∈V is called a *Whittaker vector of type* φ if $x \cdot v = \varphi (x) v$ for all $x \in \mathfrak {n}$. The $\mathfrak {g}$-module *V* is called a *Whittaker module of type* φ if it is generated by a Whittaker vector of type φ.
3. Let $\Bbbk _{\varphi } :\!\!-\Bbbk 1_\varphi$ be the one-dimensional $\mathfrak {n}$-module defined by φ; in other words, $x \cdot 1_\varphi = \varphi (x)1_\varphi$ for all $x \in \mathfrak {n}$. Then, the induced module
  $$\begin{aligned} M_\varphi :\!\!-{{\,\textrm{Ind}\,}}_\mathfrak {n}^\mathfrak {g}\Bbbk _\varphi \end{aligned}$$
  is the *universal Whittaker module of type* φ .

We proceed to briefly show how Definition 2.9 can be readily applied to filtered Lie algebras.

#### Definition 2.10

A *filtered Lie algebra* is a Lie algebra $\mathfrak {g}$ with a decreasing sequence of subalgebras of $\mathfrak {g}$

$$\begin{aligned} \dots \supseteq \mathfrak {g}_{-1} \supseteq \mathfrak {g}_0 \supseteq \mathfrak {g}_1 \supseteq \mathfrak {g}_2 \supseteq \cdots \end{aligned}$$

such that $[\mathfrak {g}_i, \mathfrak {g}_j] \subseteq \mathfrak {g}_{i + j}$. We further demand that the following two conditions are satisfied:

1. The filtration is *weakly convergent*. That is, $\bigcap _{k = 0}^\infty \mathfrak {g}_k = 0$.
2. The filtration is *bounded from below*. That is, there exists $N \in \mathbb {Z}$ such that $\mathfrak {g}_{k} = \mathfrak {g}$ for all k<N.

Using this definition, it is easy to show that filtered Lie algebras always have Whittaker modules.

#### Lemma 2.11

Let $\mathfrak {g}$ be a filtered Lie algebra as per Definition 2.10. If n≥1, then $\mathfrak {g}_n$ is a quasi-nilpotent Lie algebra, and $\mathfrak {g}/\mathfrak {g}_n$ is a locally nilpotent $\mathfrak {g}_n$-module.

In particular, one can always construct Whittaker modules for $\mathfrak {g}$ by choosing $\mathfrak {n} :=\mathfrak {g}_n$ in Definition 2.9 for any n≥1.

#### Proof

Use conditions (1) and (2) from Definition 2.10. □

We introduce a related but slightly different class of modules known as *quasi-Whittaker modules* that were recently studied in [32].

#### Definition 2.12

Let $\mathfrak {h}$ be a non-perfect ideal of a non-semisimple Lie algebra $\mathfrak {g}$, let $\varphi :\mathfrak {h}\rightarrow \Bbbk$ be a Lie algebra homomorphism, and let *V* be a $\mathfrak {g}$-module.

1. A vector v∈V is called a *quasi-Whittaker vector of type* φ if $h\cdot v = \varphi (h) v$ for all $h\in \mathfrak {h}$. The $\mathfrak {g}$-module *V* is called a *quasi-Whittaker module of type* φ if it is generated by a Whittaker vector of type φ.
2. Let $\Bbbk _{\varphi } :\!\!-\Bbbk 1_\varphi$ be the one-dimensional $\mathfrak {h}$-module defined by φ. Then, the induced module
  $$\begin{aligned} W_\varphi :\!\!-{{\,\textrm{Ind}\,}}_\mathfrak {h}^\mathfrak {g}\Bbbk _\varphi \end{aligned}$$
  is the *universal quasi-Whittaker module of type* φ .

#### Remark 2.13

Definition 2.12 applies to $\mathfrak {h}$, which need not be quasi-nilpotent, as opposed to $\mathfrak {n}$ in Definition 2.9. However, it requires that $\mathfrak {h}$ is an ideal of $\mathfrak {g}$, while $\mathfrak {n}$ need not be.

Next, we define the notion of the *Whittaker annihilator* corresponding to a Whittaker function, a key concept introduced in [32].

#### Definition 2.14

Let $\mathfrak {h}$ be a non-perfect ideal of a non-semisimple Lie algebra $\mathfrak {g}$, and let $\varphi :\mathfrak {h}\rightarrow \Bbbk$ be a Lie algebra homomorphism. The *Whittaker annihilator of* φ *over* $\mathfrak {g}$ is defined to be

$$\begin{aligned} \mathfrak {g}^\varphi :=\{x \in \mathfrak {g}\mid \varphi ([x,y]) = 0 {\text { for all }} y \in \mathfrak {h}\}. \end{aligned}$$

We conclude this section by presenting two key results from [32], which emphasise the importance of the Whittaker annihilator.

#### Theorem 2.15

([32, Theorem 3.3 and Corollary 3.12]) Let $\mathfrak {g}$ be non-semisimple Lie algebra, $\mathfrak {h}$ a non-perfect ideal of $\mathfrak {g}$, and $\varphi :\mathfrak {h}\rightarrow \Bbbk$ a Lie algebra homomorphism. Then, the following hold.

1. The universal quasi-Whittaker module $W_\varphi = {{\,\textrm{Ind}\,}}_\mathfrak {h}^\mathfrak {g}\Bbbk _\varphi$ is irreducible if and only if $\mathfrak {g}^\varphi = \mathfrak {h}$.
2. If $\dim (\mathfrak {g}^\varphi /\mathfrak {h}) = 1$, choose $y \in \mathfrak {g}^\varphi \setminus \mathfrak {h}$, so that $\mathfrak {g}^\varphi = \mathfrak {h}\oplus \Bbbk y$. Then all maximal submodules of $W_\varphi$ are of the form
  $$\begin{aligned} J_\xi :=U(\mathfrak {g})(y - \xi ) \cdot 1_\varphi \end{aligned}$$
  for $$\xi \in \Bbbk $$.

## Representations of $\mathcal {O}$ and $\widehat{\mathfrak {b}}$ by restriction

Recall that in its original form presented in [12], the BCCA emerged as the subalgebra

$$\begin{aligned} \widehat{\mathfrak {b}}:={{\,\textrm{span}\,}}\{\mathcal {O}_n:=L_n - L_{-n}, P_m :=M_m + M_{-m}, C_M \mid n \ge 1,\ m \ge 0\} \end{aligned}$$

of the BMS_3_ algebra. Hence, the most natural course of action is to study the restrictions of representations of ${{\,\textrm{Vir}\,}}$ and $\mathfrak {bms}$ to $\mathcal {O}$ and $\widehat{\mathfrak {b}}$, respectively. In doing so, we will also motivate the need for the alternative, intrinsic approaches of constructing representations of $\mathfrak {b}$ presented in this paper. Since the representation theory of ${{\,\textrm{Vir}\,}}$ and $\mathfrak {bms}$ is fairly extensive, we restrict our attention to the most popular choices of modules over these algebras, namely the Verma modules (over both) and the so-called massive modules (over $\mathfrak {bms}$).

### $\mathcal {O}$-modules via restriction of Verma modules

We first recall the definition of a Verma module of the Virasoro algebra. Recalling Definition 2.1, the Virasoro algebra admits a triangular decomposition

$$\begin{aligned} {{\,\textrm{Vir}\,}}= {{\,\textrm{Vir}\,}}_+ \oplus {{\,\textrm{Vir}\,}}_0 \oplus {{\,\textrm{Vir}\,}}_{-}, {\text { where }} {{\,\textrm{Vir}\,}}_{\pm } = \bigoplus _{n \in \mathbb {Z}_\pm } \Bbbk L_{n} {\text { and }} {{\,\textrm{Vir}\,}}_0 = \Bbbk L_0 \oplus \Bbbk C. \end{aligned}$$

#### Definition 3.1

Let $h,c \in \Bbbk$. Define the one-dimensional ${{\,\textrm{Vir}\,}}_0\oplus {{\,\textrm{Vir}\,}}_+$-module $\Bbbk _{h,c} :=\Bbbk |h,c\rangle$, where

$$\begin{aligned} L_0|h,c\rangle = h|h,c\rangle , \quad C|h,c\rangle = c|h,c\rangle , \quad {\text {and }} {{\,\textrm{Vir}\,}}_+|h,c\rangle =0. \end{aligned}$$

The *Verma module of the Virasoro algebra* *V*(*h*, *c*) is the ${{\,\textrm{Vir}\,}}$-module induced from $\Bbbk _{h,c}$. That is,

$$\begin{aligned} V(h,c) :={{\,\textrm{Ind}\,}}_{{{\,\textrm{Vir}\,}}_0\oplus {{\,\textrm{Vir}\,}}_+}^{{{\,\textrm{Vir}\,}}} \Bbbk _{h,c}. \end{aligned}$$

#### Remark 3.2

For all $h,c \in \Bbbk$, the module *V*(*h*, *c*) admits a Poincaré–Birkhoff–Witt (PBW) basis of monomials

3.1 $$\begin{aligned} L_{-n_k} \dots L_{-n_1} |h,c\rangle , \end{aligned}$$

where $n_k \ge n_{k-1} \ge \dots \ge n_1\ge 1$. Therefore, *V*(*h*, *c*) is cyclically generated by the action of ${{\,\textrm{Vir}\,}}_-$ on |h,c⟩. As left $U({{\,\textrm{Vir}\,}})$-modules,

$$\begin{aligned} V(h,c) \cong U({{\,\textrm{Vir}\,}}) / \big (U({{\,\textrm{Vir}\,}}) \cdot (L_{n>0}, L_0 - h, C - c)\big ). \end{aligned}$$

The following proposition describes *V*(*h*, *c*) as an $\mathcal {O}$-module.

#### Proposition 3.3

The Verma module *V*(*h*, *c*) is a free $U(\mathcal {O})$-module of rank 1 for all $h,c \in \Bbbk$.

#### Proof

Consider the basis $\{\mathcal {O}_n, L_m, C \mid n \ge 1, m \ge 0\}$ of ${{\,\textrm{Vir}\,}}$. This then induces a different PBW basis on $U({{\,\textrm{Vir}\,}})$:

$$\begin{aligned} \mathcal {O}_{n_k} \dots \mathcal {O}_{n_1} L_{m_\ell } \dots L_{m_1} C^r, \end{aligned}$$

where $1 \le n_1 \le \dots \le n_k$, $0 \le m_1 \le \dots \le m_\ell$, and $r \in \mathbb {N}$. In this PBW basis, the description of *V*(*h*, *c*) given in Remark 3.2 as a quotient of $U({{\,\textrm{Vir}\,}})$ by a left ideal readily translates to the fact that

$$\begin{aligned} \{\mathcal {O}_{n_k} \dots \mathcal {O}_{n_1} |h,c\rangle \mid 1 \le n_1 \le \dots \le n_k\} \end{aligned}$$

is a basis for *V*(*h*, *c*) with no relations on the $\mathcal {O}_n$s. This proves the proposition. □

Free modules are not the most interesting from a representation-theoretic point of view as they are not irreducible. This motivates our approach in the later sections, where we change basis and construct $\mathcal {O}$-modules as modules of a subalgebra of $\mathbb {W}_1$ that is isomorphic to $\mathcal {O}$. This will give us explicit examples of classes of irreducible $\mathcal {O}$-modules.

### $\widehat{\mathfrak {b}}$-modules via restriction of Verma modules

Just like the Virasoro algebra, the BMS_3_ algebra is also a graded Lie algebra: we have $\mathfrak {bms}= \bigoplus _{n\in \mathbb {Z}} \mathfrak {bms}_n$, where $\mathfrak {bms}_{n} :=\Bbbk L_n \oplus \Bbbk M_n$ for n≠0, and $\mathfrak {bms}_0:=\Bbbk L_0 \oplus \Bbbk M_0 \oplus \Bbbk C_L \oplus \Bbbk C_M$. Its triangular decomposition is given by $\mathfrak {bms}= \mathfrak {bms}_+ \oplus \mathfrak {bms}_0 \oplus \mathfrak {bms}_{-}$, where $\mathfrak {bms}_{\pm } = \bigoplus _{n \in \mathbb {Z}_\pm } \Bbbk L_n \oplus \Bbbk M_n$.

#### Definition 3.4

Let $h_L, h_M, c_L, c_M \in \Bbbk$. Define the one-dimensional $\mathfrak {bms}_0 \oplus \mathfrak {bms}_+$-module

$$\begin{aligned} \Bbbk _{h_L,h_M,c_L,c_M} :=\Bbbk |h_L, h_M, c_L, c_M\rangle , \end{aligned}$$

where

$$\begin{aligned} \begin{aligned}&L_0|h_L, h_M, c_L, c_M\rangle = h_L|h_L, h_M, c_L, c_M\rangle , \quad  &   M_0|h_L, h_M, c_L, c_M\rangle = h_M|h_L, h_M, c_L, c_M\rangle , \\&C_L|h_L, h_M, c_L, c_M\rangle = c_L|h_L, h_M, c_L, c_M\rangle , \quad  &   C_M|h_L, h_M, c_L, c_M\rangle = c_M|h_L, h_M, c_L, c_M\rangle , \end{aligned} \end{aligned}$$

and $L_{n>0} |h_L, h_M, c_L, c_M\rangle = M_{n>0} |h_L, h_M, c_L, c_M\rangle = 0$. The *Verma module of the BMS*_3_ *algebra* $V(h_L,h_M,c_L,c_M)$ is the $\mathfrak {bms}$-module induced from $\Bbbk _{h_L,h_M,c_L,c_M}$. That is,

$$\begin{aligned} V(h_L,h_M,c_L,c_M) :={{\,\textrm{Ind}\,}}_{\mathfrak {bms}_0\oplus \mathfrak {bms}_+}^{\mathfrak {bms}}\Bbbk _{h_L,h_M,c_L,c_M}. \end{aligned}$$

The Verma module $V(h_L,h_M,c_L,c_M)$ admits a Poincaré–Birkhoff–Witt basis of monomials

$$\begin{aligned} L_{-n_k} \dots L_{-n_1}M_{-m_\ell } \dots M_{-m_1}|h_L, h_M, c_L, c_M\rangle , \end{aligned}$$

where $n_k \ge n_{k-1} \ge \dots \ge n_1\ge 1$, $m_\ell \ge m_{\ell -1} \ge \dots m_1 \ge 1$. $V(h_L,h_M,c_L,c_M)$ is therefore cyclically generated by the action of $\mathfrak {bms}_-$ on $|h_L, h_M, c_L, c_M\rangle$. As left $U(\mathfrak {bms})$-modules,

$$\begin{aligned} V(h_L,h_M,c_L,c_M) \cong U(\mathfrak {bms}) / \big (U(\mathfrak {bms}) \cdot (L_{n>0},M_{n>0}, L_0-h_L, M_0-h_M, C_L-c_L, C_M-c_M)\big ). \end{aligned}$$

The following proposition describes $V(h_L,h_M,c_L,c_M)$ as a $\widehat{\mathfrak {b}}$-module.

#### Proposition 3.5

As $$\widehat{\mathfrak {b}}$$-modules,

$$\begin{aligned} V(h_L, h_M, c_L, c_M) \cong \frac{U(\widehat{\mathfrak {b}})}{U(\widehat{\mathfrak {b}})\cdot (P_0-2h_M, C_M - c_M)}. \end{aligned}$$

#### Proof

Similar to the proof of Proposition 3.3, with the only relations given by the ideal by which we quotient coming from $P_0, C_M \in \widehat{\mathfrak {b}}$. □

Certainly, one could analyse the structure of the irreducible quotient of a reducible $\mathfrak {bms}$-Verma module upon restriction to $\widehat{\mathfrak {b}}$. However, as shown in [55–57, 73], the structure of such irreducible quotients is rather complicated, so an explicit description of this $\widehat{\mathfrak {b}}$-module would require careful analysis. That being said, we do not expect such $\widehat{\mathfrak {b}}$-modules to be particularly interesting from a representation-theoretic perspective, similarly to the free or “almost-free” modules appearing in Propositions 3.3 and 3.5.

### $\widehat{\mathfrak {b}}$-modules via restriction of massive modules

We consider a class of BMS_3_ modules which we call massive modules,^3^ [22, 23, 34], defined as follows.

#### Definition 3.6

Let $\mathfrak {h}$ denote the subalgebra of $\mathfrak {bms}$ spanned by $\{M_n \mid n\in \mathbb {Z}\}$ and $L_0$. In other words, $\mathfrak {h}$ is the *extension-by-derivation* of the abelian ideal I(0,-1) of $\mathfrak {bms}$ spanned by $\{M_n \mid n \in \mathbb {Z}\}$, where ${{\,\textrm{ad}\,}}_{L_0}$ is the derivation by which it is extended. This is summarised by the short exact sequenceLet $\widehat{\mathfrak {h}}$ be the subalgebra $\mathfrak {h}\oplus \Bbbk C_L \oplus \Bbbk C_M$. Letting $\textbf{M}, \textbf{s}, c_L, c_M \in \Bbbk$, consider the one-dimensional $\widehat{\mathfrak {h}}$-module

$$\begin{aligned} \widetilde{\Bbbk }_{\textbf{M}, \textbf{s}, c_L, c_M} = \Bbbk |\textbf{M}, \textbf{s}\rangle , \end{aligned}$$

where

$$\begin{aligned} \begin{aligned}&L_0|\textbf{M}, \textbf{s}\rangle = \textbf{s}|\textbf{M}, \textbf{s}\rangle , \quad  &   M_0|\textbf{M}, \textbf{s}\rangle = \textbf{M}|\textbf{M}, \textbf{s}\rangle , \\&C_L|\textbf{M}, \textbf{s}\rangle = c_L|\textbf{M}, \textbf{s}\rangle , \quad  &   C_M|\textbf{M}, \textbf{s}\rangle = c_M|\textbf{M}, \textbf{s}\rangle , \end{aligned} \end{aligned}$$

and $M_{n}|\textbf{M}, \textbf{s}\rangle = 0$ for n≠0. The $\mathfrak {bms}$-modules $\widetilde{V}(\textbf{M}, \textbf{s}, c_L, c_M)$ induced from $\widetilde{\Bbbk }_{\textbf{M}, \textbf{s},c_L,c_M}$ are called *massive modules* [8, 22, 23, 34]. That is,

$$\begin{aligned} \widetilde{V}(\textbf{M}, \textbf{s}, c_L, c_M) :={{\,\textrm{Ind}\,}}_{\widehat{\mathfrak {h}}}^{\mathfrak {bms}} \widetilde{\Bbbk }_{\textbf{M},\textbf{s},c_L,c_M}. \end{aligned}$$

The massive module $\widetilde{V}(\textbf{M}, \textbf{s}, c_L, c_M)$ admits a PBW basis of monomials

$$\begin{aligned} L_{n_k} \dots L_{n_1} |\textbf{M}, \textbf{s}\rangle , \end{aligned}$$

where $$n_1,\dots , n_k \in \mathbb {Z}\setminus \{0\}$$ with $$n_k \ge \dots \ge n_1$$.

The following result characterises the reducibility of the massive modules in terms of the parameters $\textbf{M},\textbf{s},c_L,c_M$. To the best of our knowledge, this result has not been explicitly stated and proven in the literature before.

#### Theorem 3.7

Let $\textbf{M},\textbf{s},c_L,c_M \in \Bbbk$. The massive $\mathfrak {bms}$-module $\widetilde{V}(\textbf{M},\textbf{s},c_L,c_M)$ is irreducible if and only if $\textbf{M}+\frac{n^2-1}{24}c_M\ne 0$ for any positive integer *n*.

#### Proof

We would like to apply Theorem 2.15. Hence, we first aim to relate $\widetilde{V}(\textbf{M},\textbf{s},c_L,c_M)$ to a universal quasi-Whittaker module of $\mathfrak {bms}$ as per Definition 2.12. Consider the ideal $\mathfrak {a}:={{\,\textrm{span}\,}}\{C_M, C_L, M_k \mid k \in \mathbb {Z}\}$ of $\mathfrak {bms}$ and define the Whittaker function $\phi :\mathfrak {a}\rightarrow \Bbbk$ given by

$$\begin{aligned} \phi (M_i) = \delta _{i,0} \textbf{M}, \quad \phi (C_L) = c_L, \quad \phi (C_M) = c_M. \end{aligned}$$

Letting $W_\phi :={{\,\textrm{Ind}\,}}_\mathfrak {a}^\mathfrak {bms}\Bbbk _\phi$ be the associated universal quasi-Whittaker module, it is easy to see that

$$\begin{aligned} \widetilde{V}(\textbf{M}, \textbf{s},c_L,c_M) \cong W_\phi /J_{\textbf{s}}, \end{aligned}$$

where $$J_{\textbf{s}} :=U(\mathfrak {bms})(L_0 - \textbf{s}) \cdot 1_\phi $$.

Suppose first that $\textbf{M} + \frac{n^2-1}{24}c_M \ne 0$ for any positive integer *n*. By setting n=1, we see that $\textbf{M} \ne 0$. From Definition 2.14, we may quickly deduce that the Whittaker annihilator of ϕ is $\mathfrak {a}\oplus \Bbbk L_0$. Now, Theorem 2.15 implies that $J_{\textbf{s}}$ is a maximal $\mathfrak {bms}$-submodule of $W_\phi$. Therefore, the quotient $\widetilde{V}(\textbf{M}, \textbf{s},c_L,c_M) \cong W_\phi /J_{\textbf{s}}$ is irreducible.

Conversely, assume that $\textbf{M} + \frac{n^2 - 1}{24} c_M = 0$ for a positive integer *n*. It is easy to compute that the Whittaker annihilator of ϕ contains $\mathfrak {a}\oplus \Bbbk L_0 \oplus \Bbbk L_n \oplus \Bbbk L_{-n}$. Therefore, we see that $L_{n}|\textbf{M}, \textbf{s}\rangle$ generates a nonzero proper submodule of $\widetilde{V}(\textbf{M}, \textbf{s},c_L,c_M)$. Thus, in this case, $\widetilde{V}(\textbf{M}, \textbf{s},c_L,c_M)$ is reducible. □

#### Remark 3.8

By comparing [57, Theorem 2.3] with Theorem 3.7, we see that the massive modules $\widetilde{V}(\textbf{M}, \textbf{s}, c_L, c_M)$ are irreducible under the same conditions as the Verma modules of $\mathfrak {bms}$ from Definition 3.4. This is an unexpected, intriguing similarity between two very different classes of $\mathfrak {bms}$-modules that could potentially be explained by structural features of these modules that are yet to be discovered.

Using the findings from the proof of Theorem 3.7 and another result from [32], it is not difficult to show that $L_n |\textbf{M}, \textbf{s}\rangle$ and $L_{-n} |\textbf{M}, \textbf{s}\rangle$ together generate a maximal submodule of the massive module $\widetilde{V}(\textbf{M}, \textbf{s}, c_L, c_M)$ when $\textbf{M} + \frac{n^2 - 1}{24}c_M = \textbf{s} + \frac{n^2 - 1}{24}c_L = 0$ and $c_M \ne 0$.

#### Corollary 3.9

Let $\textbf{M}, \textbf{s}, c_L, c_M \in \Bbbk$ with $c_M \ne 0$, and suppose $\textbf{M} + \frac{n^2 - 1}{24}c_M = \textbf{s} + \frac{n^2 - 1}{24} c_L = 0$ for some positive integer *n*. Then, the $\mathfrak {bms}$-submodule of the massive module $\widetilde{V}(\textbf{M}, \textbf{s}, c_L, c_M)$ generated by $L_n|\textbf{M}, \textbf{s}\rangle$ and $L_{-n}|\textbf{M}, \textbf{s}\rangle$ is a maximal submodule of $\widetilde{V}(\textbf{M}, \textbf{s}, c_L, c_M)$.

#### Proof

Let $\phi :\mathfrak {a}\rightarrow \Bbbk$ be as in the proof of Theorem 3.7. Let

$$\begin{aligned} \widetilde{\mathfrak {a}} :={{\,\textrm{span}\,}}\{L_0, L_n, L_{-n}, M_k, C_M, C_L \mid k \in \mathbb {Z}\} = \mathfrak {a}\oplus \Bbbk L_0 \oplus \Bbbk L_n \oplus \Bbbk L_{-n}, \end{aligned}$$

and define $\Phi :\widetilde{\mathfrak {a}} \rightarrow \Bbbk$ by ${ \left. \hspace{0.0pt}\Phi \phantom {\big |} \right| _{\mathfrak {a}} } = \phi$, and

$$\begin{aligned} \Phi (L_0) = \textbf{s}, \quad \Phi (L_n) = \Phi (L_{-n}) = 0. \end{aligned}$$

One can compute that $\mathfrak {bms}^\Phi = \widetilde{\mathfrak {a}}$. The condition that $\textbf{s} + \frac{n^2 - 1}{24}c_L = 0$ guarantees that Φ is a well-defined Lie algebra homomorphism, since

$$\begin{aligned} \Phi ([L_n, L_{-n}]) = \Phi \left( 2n L_0 + \frac{n^3 - n}{12} C_L\right) = 2n \textbf{s} + \frac{n^3 - n}{12} c_L = 0. \end{aligned}$$

Therefore, Φ extends the quasi-Whittaker function ϕ to the entirety of its Whittaker annihilator. By [32, Lemma 3.7], it follows that ${{\,\textrm{Ind}\,}}_{\widetilde{\mathfrak {a}}}^\mathfrak {bms}\Bbbk _{\Phi }$ is an irreducible $\mathfrak {bms}$-module. It is easy to see that this module is isomorphic to the quotient of $\widetilde{V}(\textbf{M}, \textbf{s}, c_L, c_M)$ by its submodule generated by $L_n|\textbf{M}, \textbf{s}\rangle$ and $L_{-n}|\textbf{M}, \textbf{s}\rangle$, yielding the result. □

#### Remark 3.10

We were informed by Blagoje Oblak that Theorem 3.7 and Corollary 3.9 match the intuitive expectation stemming from his work [23] with Glenn Barnich, in which the authors study coadjoint orbits of the BMS_3_ group and provide classical and quantum mechanical interpretations of these findings in the context of three-dimensional asymptotically flat gravity. It is reassuring that we are able to make this physical intuition mathematically precise.

We now consider the reducibility of the $\widehat{\mathfrak {b}}$-submodule of $\widetilde{V}(\textbf{M}, \textbf{s}, c_L, c_M)$ generated by the element $|\textbf{M}, \textbf{s}\rangle$. The condition governing its reducibility turns out to be exactly the same as the one in Theorem 3.7, and the proof is easier, since $U(\widehat{\mathfrak {b}})|\textbf{M}, \textbf{s}\rangle$ is a quasi-Whittaker module over $\widehat{\mathfrak {b}}$ on the nose.

#### Proposition 3.11

Let $\textbf{M}, \textbf{s},c_L,c_M \in \Bbbk$. The $\widehat{\mathfrak {b}}$-module $U(\widehat{\mathfrak {b}})|\textbf{M}, \textbf{s}\rangle$ is irreducible if and only if $\textbf{M} + \frac{n^2 - 1}{24}c_M \ne 0$ for any positive integer *n*.

#### Proof

Suppose first that $\textbf{M} +\frac{n^2-1}{24}c_M\ne 0$ for any positive integer *n*. Again, setting n=1 lets us infer that $\textbf{M} \ne 0$. Take $\mathfrak {a}:={{\,\textrm{span}\,}}\{C_M, P_k:k\ge 0\}$, which is an ideal of $\widehat{\mathfrak {b}}$, and define a linear map $\phi :\mathfrak {a}\rightarrow \Bbbk$ by

$$\begin{aligned} \phi (P_i) = 2\delta _{i,0}\textbf{M}, \quad \phi (C_M) = c_M. \end{aligned}$$

The Whittaker annihilator of ϕ, denoted $\widehat{\mathfrak {b}}^\phi$, is easily computed to be $\widehat{\mathfrak {b}}^\phi = \mathfrak {a}$. It now follows immediately from Theorem 2.15 that the $\widehat{\mathfrak {b}}$-module $U(\widehat{\mathfrak {b}})|\textbf{M}, \textbf{s}\rangle \cong W_\phi = {{\,\textrm{Ind}\,}}_\mathfrak {a}^{\widehat{\mathfrak {b}}} \Bbbk _\phi$ is irreducible.

Conversely, assume that $\textbf{M} +\frac{n^2 - 1}{24}c_M = 0$ for a positive integer *n*. In this case, $\widehat{\mathfrak {b}}^\phi \supseteq \mathfrak {a}\oplus \Bbbk \mathcal {O}_n$. Then, from Theorem 2.15, we see that $\mathcal {O}_{n}|\textbf{M}, \textbf{s}\rangle$ generates a nonzero proper submodule of $U(\widehat{\mathfrak {b}})|\textbf{M}, \textbf{s}\rangle$. Thus, $U(\widehat{\mathfrak {b}})|\textbf{M}, \textbf{s}\rangle$ is reducible in this case. □

Thus, the massive modules of $\mathfrak {bms}$ seem to give rise to irreducible $\widehat{\mathfrak {b}}$-modules more readily than the Verma modules.

We finish our analysis of massive modules by studying the full space $\widetilde{V}(\textbf{M},\textbf{s},c_L,c_M)$ as a module over $\widehat{\mathfrak {b}}$. In determining the structure $\widetilde{V}(\textbf{M},\textbf{s},c_L,c_M)$ as a $\widehat{\mathfrak {b}}$-module, we assume that $\textbf{M} + \frac{n^2 - 1}{24}c_M \ne 0$ for any positive integer *n* for the rest of this section, meaning that $U(\widehat{\mathfrak {b}})|\textbf{M}, \textbf{s}\rangle$ is irreducible. We first introduce two new symbols.

#### Notation 3.12

For $$n \in \mathbb {Z}$$, define

$$\begin{aligned} \widehat{P}_n :=M_n - M_{-n}, \quad \widehat{\mathcal {O}}_n :=L_n + L_{-n}, \end{aligned}$$

as elements of $\mathfrak {bms}$. Note that $\widehat{P}_n$ and $\widehat{\mathcal {O}}_n$ are not elements of $\widehat{\mathfrak {b}}$.

By (2.1), a simple computation shows that for any $n,m \in \mathbb {N}$ we have

3.2 $$\begin{aligned} \begin{aligned}&{[}P_n, \widehat{\mathcal {O}}_m] = (n - m)\widehat{P}_{n + m} + (n + m)\widehat{P}_{n - m}, \\&{[}\widehat{P}_n, \widehat{\mathcal {O}}_m] = (n - m){P}_{n + m} + (n + m){P}_{n - m} + \frac{1}{6} n (n^2 - 1)\delta _{n,m} C_M. \end{aligned} \end{aligned}$$

Notice that $\{\mathcal {O}_n, \widehat{\mathcal {O}}_m, P_m, \widehat{P}_n \mid n \in \mathbb {Z}_+, m \in \mathbb {N}\}$ is another basis of $\mathfrak {bms}$. Hence, by the PBW theorem, $\widetilde{V}(\textbf{M}, \textbf{s}, c_L, c_M)$ has a basis given by monomials of the form

3.3 $$\begin{aligned} \mathcal {O}_{p_k}^{q_k} \dots \mathcal {O}_{p_1}^{q_1} \widehat{\mathcal {O}}^{s_\ell }_{r_\ell } \dots {\widehat{\mathcal {O}}}^{s_1}_{r_1} |\textbf{M}, \textbf{s}\rangle , \end{aligned}$$

where $$p_k> \dots > p_1 \ge 1$$, $$r_\ell> \dots > r_1 \ge 1$$ and $$q_1, \dots , q_k, s_1, \dots , s_\ell \in \mathbb {N}$$.

We now prove that the element $\widehat{\mathcal {O}}_n |\textbf{M}, \textbf{s}\rangle$ of $\widetilde{V}(\textbf{M}, \textbf{s}, c_L, c_M)$ generates an irreducible $\widehat{\mathfrak {b}}$-submodule, for all $n \in \mathbb {Z}_+$.

#### Proposition 3.13

For any $n \in \mathbb {Z}_+$, the $\widehat{\mathfrak {b}}$-module $U(\widehat{\mathfrak {b}}) \widehat{\mathcal {O}}_n |\textbf{M}, \textbf{s}\rangle$ is isomorphic to $U(\widehat{\mathfrak {b}}) |\textbf{M}, \textbf{s}\rangle$. Consequently, $U(\widehat{\mathfrak {b}}) \widehat{\mathcal {O}}_n |\textbf{M}, \textbf{s}\rangle$ is an irreducible $\widehat{\mathfrak {b}}$-module.

#### Proof

It follows from (3.2) that for any $r\in \mathbb {N}$,

$$\begin{aligned} P_r \widehat{\mathcal {O}}_n |\textbf{M}, \textbf{s}\rangle = \delta _{0,r} 2M |\textbf{M}, \textbf{s}\rangle . \end{aligned}$$

As above, the PBW theorem implies that $U(\widehat{\mathfrak {b}}) \widehat{\mathcal {O}}_n |\textbf{M}, \textbf{s}\rangle$ has a basis given by

$$\begin{aligned} \mathcal {O}_{p_k}^{q_k} \dots \mathcal {O}_{p_1}^{q_1} \widehat{\mathcal {O}}_n|\textbf{M}, \textbf{s}\rangle , \end{aligned}$$

where $p_k> \dots > p_1 \ge 1$, and $q_1, \dots , q_k\in \mathbb {N}$. Therefore, we see that the map

$$\begin{aligned} U(\widehat{\mathfrak {b}}) \widehat{\mathcal {O}}_n |\textbf{M}, \textbf{s}\rangle&\rightarrow U(\widehat{\mathfrak {b}}) |\textbf{M}, \textbf{s}\rangle \\ \mathcal {O}_{p_k}^{q_k} \dots \mathcal {O}_{p_1}^{q_1} \widehat{\mathcal {O}}_n |\textbf{M}, \textbf{s}\rangle&\mapsto \mathcal {O}_{p_k}^{q_k} \dots \mathcal {O}_{p_1}^{q_1} |\textbf{M}, \textbf{s}\rangle , \end{aligned}$$

where $p_k> \dots > p_1 \ge 1$ and $q_1, \dots , q_k \in \mathbb {N}$, is an isomorphism of $\widehat{\mathfrak {b}}$-modules.

The final sentence follows from Proposition 3.11 upon recalling that we are assuming that $\textbf{M} + \frac{n^2 - 1}{24}c_M \ne 0$. □

It is not surprising that the massive $\mathfrak {bms}$-module $\widetilde{V}(\textbf{M}, \textbf{s}, c_L, c_M)$ does not remain irreducible when viewed as a $\widehat{\mathfrak {b}}$-module. What is perhaps more surprising is that $\widetilde{V}(\textbf{M}, \textbf{s}, c_L, c_M)$ decomposes into a direct sum of two $\widehat{\mathfrak {b}}$-submodules, as we will soon see. We define these submodules below.

#### Definition 3.14

Let $\widehat{V}$ be the $\widehat{\mathfrak {b}}$-submodule of $\widetilde{V}(\textbf{M}, \textbf{s}, c_L, c_M)$ generated by

$$\begin{aligned} \left\{ \widehat{\mathcal {O}}^{s_\ell }_{r_\ell } \dots \widehat{\mathcal {O}}^{s_1}_{r_1} |\textbf{M}, \textbf{s}\rangle \mid \ell \in \mathbb {N}, r_\ell> \dots > r_1 \ge 1, s_1 + \dots + s_\ell = 0 \pmod 2\right\} , \end{aligned}$$

and let $\widehat{W}$ be the $\widehat{\mathfrak {b}}$-submodule of $\widetilde{V}(\textbf{M}, \textbf{s}, c_L, c_M)$ generated by

$$\begin{aligned} \left\{ \widehat{\mathcal {O}}^{s_\ell }_{r_\ell } \dots \widehat{\mathcal {O}}^{s_1}_{r_1} |\textbf{M}, \textbf{s}\rangle \mid \ell \in \mathbb {N}, r_\ell> \dots > r_1 \ge 1, s_1 + \dots + s_\ell = 1 \pmod 2\right\} . \end{aligned}$$

The following result shows that the action of $P_r$ on $\widehat{\mathcal {O}}^{s_\ell }_{r_\ell } \dots \widehat{\mathcal {O}}^{s_1}_{r_1} |\textbf{M}, \textbf{s}\rangle$ preserves the parity of $\sum s_i$.

#### Proposition 3.15

For any $r, s_1,\ldots , s_{\ell }\in \mathbb {N}$ and $r_\ell> \dots > r_1 \ge 1$, the abelian part of the BMS_3_ algebra has the following action on $\widehat{\mathcal {O}}^{s_\ell }_{r_\ell } \dots \widehat{\mathcal {O}}^{s_1}_{r_1} |\textbf{M}, \textbf{s}\rangle$:

$$\begin{aligned} P_r \cdot \widehat{\mathcal {O}}^{s_\ell }_{r_\ell } \dots \widehat{\mathcal {O}}^{s_1}_{r_1} |\textbf{M}, \textbf{s}\rangle&= \delta _{0,r} 2\textbf{M} \widehat{\mathcal {O}}^{s_\ell }_{r_\ell } \dots \widehat{\mathcal {O}}^{s_1}_{r_1} |\textbf{M}, \textbf{s}\rangle&\pmod {\bigoplus _{\begin{array}{c} 0 \le u_i \le s_i, \sum u_i < \sum s_i, \\ \sum (s_i - u_i) = 0 \pmod {2} \end{array}} \Bbbk \widehat{\mathcal {O}}^{u_\ell }_{r_\ell } \dots \widehat{\mathcal {O}}^{u_1}_{r_1} |\textbf{M}, \textbf{s}\rangle }, \\ \widehat{P}_r \cdot \widehat{\mathcal {O}}^{s_\ell }_{r_\ell } \dots \widehat{\mathcal {O}}^{s_1}_{r_1} |\textbf{M}, \textbf{s}\rangle&= 0&\pmod {\bigoplus _{\begin{array}{c} 0 \le u_i \le s_i, \\ \sum (s_i - u_i) = 1 \pmod {2} \end{array}} \Bbbk \widehat{\mathcal {O}}^{u_\ell }_{r_\ell } \dots \widehat{\mathcal {O}}^{u_1}_{r_1} |\textbf{M}, \textbf{s}\rangle }. \end{aligned}$$

#### Proof

We prove the result by induction on $$d :=\sum _{i = 1}^{\ell } s_i$$. It is not difficult to check that the two equalities hold for d=0,1. Now assume that d>1 and the two equalities hold for all $0\leq d^{\prime }<d$. Consider the first equality for the case $$d = \sum _{i = 1}^{\ell } s_i$$. We have

$$\begin{aligned}&P_r \cdot \widehat{\mathcal {O}}^{s_\ell }_{r_\ell } \dots \widehat{\mathcal {O}}^{s_1}_{r_1} |\textbf{M}, \textbf{s}\rangle = [P_r, \widehat{\mathcal {O}}_{r_\ell }] \widehat{\mathcal {O}}^{s_\ell -1}_{r_\ell } \dots \widehat{\mathcal {O}}^{s_1}_{r_1} |\textbf{M}, \textbf{s}\rangle + \widehat{\mathcal {O}}_{r_\ell } P_r\widehat{\mathcal {O}}^{s_\ell - 1}_{r_\ell } \dots \widehat{\mathcal {O}}^{s_1}_{r_1} |\textbf{M}, \textbf{s}\rangle \\&\quad = \left( (r - r_{\ell }) \widehat{P}_{r + r_{\ell }} + (r + r_{\ell }) \widehat{P}_{r - r_{\ell }} + \widehat{\mathcal {O}}_{r_\ell } P_r\right) \widehat{\mathcal {O}}^{s_\ell - 1}_{r_\ell } \dots \widehat{\mathcal {O}}^{s_1}_{r_1} |\textbf{M}, \textbf{s}\rangle . \end{aligned}$$

The induction hypothesis implies that $\left( (r - r_{\ell }) \widehat{P}_{r + r_{\ell }} + (r + r_{\ell }) \widehat{P}_{r - r_{\ell }}\right) \widehat{\mathcal {O}}^{s_\ell - 1}_{r_\ell } \dots \widehat{\mathcal {O}}^{s_1}_{r_1} |\textbf{M}, \textbf{s}\rangle$ belongs to

$$\bigoplus _{\begin{array}{c} 0 \le u_i \le s_i - \delta _{i,\ell }, \\ \sum (s_i - u_i) - 1 = 1 \pmod {2} \end{array}} \Bbbk \widehat{\mathcal {O}}^{u_\ell }_{r_\ell } \dots \widehat{\mathcal {O}}^{u_1}_{r_1} |\textbf{M}, \textbf{s}\rangle \subseteq \bigoplus _{\begin{array}{c} 0 \le u_i \le s_i, \sum u_i < \sum s_i, \\ \sum (s_i - u_i) = 0 \pmod {2} \end{array}} \Bbbk \widehat{\mathcal {O}}^{u_\ell }_{r_\ell } \dots \widehat{\mathcal {O}}^{u_1}_{r_1} |\textbf{M}, \textbf{s}\rangle ,$$

and that $P_r\widehat{\mathcal {O}}^{s_\ell -1}_{r_\ell }\dots \widehat{\mathcal {O}}^{s_1}_{r_1}|\textbf{M}, \textbf{s}\rangle - \delta _{0,r} 2\textbf{M} \widehat{\mathcal {O}}^{s_\ell -1}_{r_\ell } \dots \widehat{\mathcal {O}}^{s_1}_{r_1} |\textbf{M}, \textbf{s}\rangle$ belongs to

$$\bigoplus _ {\begin{array}{c} 0 \le u_i \le s_i-\delta _{i,\ell }, \sum u_i < \sum s_i - 1, \\ \sum (s_i - u_i) - 1= 0 \pmod {2} \end{array}} \Bbbk \widehat{\mathcal {O}}^{u_\ell }_{r_\ell } \dots \widehat{\mathcal {O}}^{u_1}_{r_1} |\textbf{M}, \textbf{s}\rangle .$$

The first equality follows. The second equality can be proved similarly. □

This naturally leads to the decomposition of $\widetilde{V}(\textbf{M},\textbf{s},c_L,c_M)$ given by the following corollary.

#### Corollary 3.16

As a $\widehat{\mathfrak {b}}$-module, the massive module $\widetilde{V}(\textbf{M},\textbf{s},c_L,c_M)$ is decomposable. More precisely, we have

$$\begin{aligned} \widetilde{V}(\textbf{M},\textbf{s},c_L,c_M) = \widehat{V} \oplus \widehat{W}. \end{aligned}$$

#### Proof

From Proposition 3.15 and the PBW theorem, we may deduce that $\widehat{V}$ and $\widehat{W}$ have bases given by

$$\begin{aligned}&\left. \bigg \{\mathcal {O}_{p_k}^{q_k} \dots \mathcal {O}_{p_1}^{q_1} \widehat{\mathcal {O}}^{s_\ell }_{r_\ell } \dots {\widehat{\mathcal {O}}}^{s_1}_{r_1} |\textbf{M}, \textbf{s}\rangle \right| p_k> \dots> p_1 \ge 1, r_\ell> \dots> r_1 \ge 1, \sum _{i = 1}^\ell s_i = 0 \pmod 2\bigg \}, \\&\left. \bigg \{\mathcal {O}_{p_k}^{q_k} \dots \mathcal {O}_{p_1}^{q_1} \widehat{\mathcal {O}}^{s_\ell }_{r_\ell } \dots {\widehat{\mathcal {O}}}^{s_1}_{r_1} |\textbf{M}, \textbf{s}\rangle \right| p_k> \dots> p_1 \ge 1, r_\ell> \dots > r_1 \ge 1, \sum _{i = 1}^\ell s_i = 1 \pmod 2\bigg \}, \end{aligned}$$

respectively. Comparing this with the PBW basis of $\widetilde{V}(\textbf{M}, \textbf{s}, c_L, c_M)$ given by (3.3) leads to the result. □

It is interesting to note that the massive module $\widetilde{V}(\textbf{M},\textbf{s},c_L,c_M)$ with $\textbf{M}+\frac{n^2-1}{24} \ne 0$ for all $n \in \mathbb {Z}_+$ is irreducible as a $\mathfrak {bms}$-module but decomposable as a $\widehat{\mathfrak {b}}$-module.

We finish our discussion of $\widetilde{V}(\textbf{M},\textbf{s},c_L,c_M)$ as a $\widehat{\mathfrak {b}}$-module with the following conjecture.

#### Conjecture 3.17

The $\widehat{\mathfrak {b}}$-modules $\widehat{V}$ and $\widehat{W}$ are indecomposable.

### The $\mathcal {O}$-module $\Omega (\lambda ,a)$

We finish this section by restricting one final class of Virasoro modules to $\mathcal {O}$, first defined in [66]. For any pair $(a,\lambda )\in \Bbbk \times \Bbbk ^*$, one can define a Virasoro algebra module structure on k[X] by

$$\begin{aligned} C \cdot f(X) = 0, \quad L_n \cdot f(X) = \lambda ^n (X + na) f(X + n), \quad n \in \mathbb {Z}. \end{aligned}$$

The module is irreducible if and only if a≠0, and $\Omega (\lambda ,0)$ has a unique irreducible submodule which is isomorphic to $\Omega (\lambda ,1)$ [65, Section 4.3]. Restricting the above action to the subalgebra $\mathcal {O}$ of ${{\,\textrm{Vir}\,}}$, the $\mathcal {O}$-module structure on k[X] is given by

3.4 $$\begin{aligned} \mathcal {O}_n \cdot f(X) = \lambda ^{n}(X + na)f(X + n) - \lambda ^{-n} (X - na) f(X - n), \quad n \ge 1. \end{aligned}$$

We still denote the corresponding $\mathcal {O}$-module by $\Omega (\lambda , a)$.

#### Proposition 3.18

Suppose $\lambda \in \Bbbk ^*$ and $a\in \Bbbk$. If $\lambda \ne \pm 1$ and a≠0, then the $\mathcal {O}$-module $\Omega (\lambda ,a)$ is irreducible.

To prove Proposition 3.18, we need the following lemma from [80].

#### Lemma 3.19

([80, Lemma 2]) Let $\lambda _1, \lambda _2, \dots ,\lambda _m \in \Bbbk$, $s_1,s_2, \dots , s_m \in \mathbb {Z}_+$ with $s_1 + \dots + s_m = s$. Define a sequence of functions on $\mathbb {Z}$ as follows: $f_1(n) = \lambda _1^n, f_2(n) = n\lambda _1^n,\dots , f_{s_1}(n) = n^{s_1 - 1} \lambda _1^n, f_{s_1+1}(n) = \lambda _2^n, \dots , f_{s_1 + s_2}(n) = n^{s_2 - 1} \lambda _2^n, \dots , f_s(n) = n^{s_m - 1}\lambda _m^n$. Let $\mathcal {M}=(y_{pq})$ be the s×s matrix with $y_{pq} = f_{q}(p-1)$, q=1,2,⋯,s, p=u+1,u+2,⋯,u+s where $u \in \mathbb {N}$. Then

$$\det (\mathcal {M}) = \prod _{j = 1}^m(s_j - 1)!!\lambda _j^{s_j(s_j + 2u - 1)/2}\prod _{1 \le i < j \le m} (\lambda _j - \lambda _i)^{s_i s_j},$$

where $s_{j}!! = s_j!\times (s_j-1)! \times \dots \times 2! \times 1!$ with 0!!=1, for convenience.

In particular, we have the following.

#### Lemma 3.20

Suppose $\mu \in \Bbbk$ and $\mu \ne 0, 1$. For $r \in \mathbb {Z}_+$ and a fixed $u \in \mathbb {N}$ let $k_j = u + j, j = 1,2, \dots , 2r$. Letting

$$\tau (\mu ,r,u) :=\begin{vmatrix} 1&\quad k_1&\quad k_1^2&\quad \cdots&\quad k_1^{r-1}&\quad \mu ^{k_1}&\quad \mu ^{k_1}k_1&\quad \mu ^{k_1}k_1^2&\quad \cdots&\quad \mu ^{k_1}k_1^{r-1}\\ 1&\quad k_2&\quad k_2^2&\quad \cdots&\quad k_2^{r-1}&\quad \mu ^{k_2}&\quad \mu ^{k_2}k_2&\quad \mu ^{k_2}k_2^2&\quad \cdots&\quad \mu ^{k_2}k_2^{r-1}\\ 1&\quad k_3&\quad k_3^2&\quad \cdots&\quad k_3^{r-1}&\quad \mu ^{k_3}&\quad \mu ^{k_3}k_3&\quad \mu ^{k_3}k_3^2&\quad \cdots&\quad \mu ^{k_3}k_3^{r-1}\\ \vdots&\quad \vdots&\quad \vdots&\quad \ddots&\quad \vdots&\quad \vdots&\quad \vdots&\quad \vdots&\quad \ddots&\quad \vdots \\ 1&\quad k_{2r}&\quad k_{2r}^2&\quad \cdots&\quad k_{2r}^{r-1}&\quad \mu ^{k_{2r}}&\quad \mu ^{k_{2r}}k_{2r}&\quad \mu ^{k_{2r}}k_{2r}^2&\quad \cdots&\quad \mu ^{k_{2r}}k_{2r}^{r-1}\\ \end{vmatrix},$$

then $$\tau (\mu ,r,u)\ne 0$$.

#### Proof

Taking m=2, $\lambda _1=1,\lambda _2=\mu$ and $s_1=s_2=r$ in Lemma 3.19 we see $\tau (\mu ,r,u)=\det \big ((y_{pq})_{2r\times 2r }\big )$ with $y_{pq}=f_{q}(p-1)$ and $q=1, 2, \dots , 2r, p=u+1,u+2,\dots , u+2r$. So

$$\tau (\mu ,r,u)=\big ((r-1)!!\big )^2\mu ^{r(r+2u-1)/2}(\mu -1)^{r^2}\ne 0, $$

as desired. This completes the proof. □

As the following result shows, the key difference between the cases where $\lambda = \pm 1$ or a=0 and the other cases is whether $\Omega (\lambda , a)$ is generated by 1.

#### Lemma 3.21

If $\lambda \ne \pm 1$ and a≠0, then the $\mathcal {O}$-module $\Omega (\lambda ,a)$ is generated by 1.

#### Proof

It is sufficient to show that $$X^n\in U(\mathcal {O}) \cdot 1$$ for any $$n \in \mathbb {N}.$$ We prove this by induction on *n*, the base case n=0 being trivial. For the induction step, let n>0 and assume $$X^{n'} \in U(\mathcal {O}) \cdot 1$$ for any $0\leq n^{\prime }<n$. We aim to show that $$X^n \in U(\mathcal {O}) \cdot 1.$$ Since $$X^{n - 1} \in U(\mathcal {O}) \cdot 1$$, it follows from (3.4) that

$$\begin{aligned} \lambda ^k \mathcal {O}_k \cdot X^{n-1}&= \lambda ^{2k}(X + ka)(X + k)^{n - 1} - (X - ka)(X - k)^{n - 1} \\&= (\lambda ^{2k} - 1)X^n + g(X) \in U(\mathcal {O}) \cdot 1 \end{aligned}$$

for some $g(X) \in \Bbbk [X]$. Note that $g(X) \in U(\mathcal {O}) \cdot 1$ by the induction hypothesis, since $\deg (g(X)) \le n - 1$. Therefore,

$$\begin{aligned} (\lambda ^{2k} - 1) X^n = \lambda ^k \mathcal {O}_k \cdot X^{n - 1} - g(X) \in U(\mathcal {O}) \cdot 1. \end{aligned}$$

Since $\lambda \ne \pm 1$, there always exists k≥1 such that $(\lambda ^{2k} - 1) \ne 0$, which implies that $X^n \in U(\mathcal {O}) \cdot 1$. Thus, $U(\mathcal {O}) \cdot 1 = \Omega (\lambda ,a)$. This completes the proof. □

Now we can prove Proposition 3.18.

#### Proof of Proposition 3.18

To show that $\Omega (\lambda , a)$ is simple is enough to show that any nonzero submodule of $\Omega (\lambda , a)$ is the module $\Omega (\lambda , a)$ itself. Let *M* be a nonzero submodule of $\Omega (\lambda , a)$. To show $M=\Omega (\lambda , a)$ is, by Lemma 3.21, to show 1∈M. To the contrary, assume that 1∉M. Let f(X)∈M be a nonzero element with minimal degree r>0. Denote $f(X) = \sum _{i=0}^r c_i X^i$ with $c_i \in \Bbbk$ and $c_r \ne 0$. Then by (3.4), we have

3.5 $$\begin{aligned} \begin{aligned} \lambda ^k \mathcal {O}_k \cdot f(X)&= \lambda ^{2k}(X + ka) f(X + k) - (X - ka) f(X - k)\\&= \sum _{i = 0}^{r + 1} \varphi _{i}(X) \lambda ^{2k} k^i + \sum _{i = 0}^{r + 1} \psi _i(X)k^i \in M\\ \end{aligned} \end{aligned}$$

for all $k \in \mathbb {Z}_+$, where $\varphi _i(X), \psi _i(X) \in \Bbbk [X]$ and $\varphi _{r + 1}(X) = a c_r$ and $\psi _{r + 1}(X) = (-1)^r a c_r$ are nonzero constants. Let $\mu = \lambda ^2$, $k_j = j$, where $1 \le j \le 2(r + 2)$ and $\mu \ne 0, 1$. By Lemma 3.20, we know that $\tau (\mu , r+2, 0)\ne 0$, which implies that $\varphi _i(X),\psi _i(X)\in M$ for all $0 \le i \le r + 1$. In particular, $\varphi _{r + 1}(X), \psi _{r + 1}(X)\in M$, contrary to the choice of *f*(*X*). Therefore, 1∈M. So $M = \Omega (\lambda ,a)$ and $\Omega (\lambda ,a)$ is a simple $\mathcal {O}$-module. This completes the proof. □

Finally, we consider the case $\lambda = \pm 1.$

#### Proposition 3.22

If $\lambda = \pm 1$ and a≠0, then the $\mathcal {O}$-module $\Omega (\lambda ,a)$ carries a filtration of submodules of $\Omega (\lambda ,a)$ as follows:

$$\begin{aligned} \Omega (\lambda ,a)_n \subseteq \Omega (\lambda ,a)_{n + 1}, \quad n \in \mathbb {N}, \end{aligned}$$

where

$$\begin{aligned} \Omega (\lambda ,a)_n :=\{f(X) \in \Omega (\lambda ,a) \mid \deg f(X) \le n\} = \left\{ \sum _{i=0}^n c_i X^i \mid c_i \in \Bbbk \right\} . \end{aligned}$$

#### Proof

We only need to show that each $\Omega (\lambda ,a)_n$ is a submodule of $\Omega (\lambda ,a)$, which follows from the fact that for any k≥1 and any nonzero $f(X) \in \Bbbk [X]$,

$$\begin{aligned} \mathcal {O}_k\cdot f(X) = \lambda ^k \Big (X(f(X + k) - f(X - k)) + k a (f(X + k) + f(X - k))\Big ) \end{aligned}$$

has degree $\le \deg (f(X))$ for $\deg (f(X+k)-f(X-k))=\deg (f(X))-1$ if $\deg (f(X))\ge 1$. □

#### Remark 3.23

Since $\Omega (\lambda ,0)$ as a ${{\,\textrm{Vir}\,}}$-module has a submodule Xk[X], which is isomorphic to $\Omega (\lambda , 1)$, we see that $\Omega (\lambda ,0)$ as a $\mathcal {O}$-module also has a submodule $X\Bbbk [X]\cong \Omega (\lambda ,1)$. If $\lambda \ne \pm 1$, then, by Proposition 3.18, Xk[X] is a simple $\mathcal {O}$-module; and if $\lambda =\pm 1$, then, by Proposition 3.22, $\Omega (\lambda ,0)$ as an $\mathcal {O}$-module has a filtration of submodules:

$$\begin{aligned} X\Omega (\lambda ,0)_0 \subseteq X\Omega (\lambda ,0)_1 \subseteq \dots \subseteq X\Omega (\lambda ,0)_n \subseteq \dots \subseteq X\Omega (\lambda ,0) \subseteq \Omega (\lambda ,0). \end{aligned}$$

## Alternate basis of the BCCA

Our next goal is to construct a decreasing filtration on $\mathcal {O}$ and $\mathfrak {b}$, which will allow us to define and study Whittaker modules for these Lie algebras as per Lemma 2.11. Unfortunately, the basis of $\mathfrak {b}$ from Sect. 2 makes it difficult to construct the desired filtration. Therefore, in this section we present an alternate basis for $\mathcal {O}$ and $\mathfrak {b}$, from which the decreasing filtration becomes manifest.

### Subalgebras of the Witt algebra

Since $\mathfrak {b}= \mathcal {O}\ltimes \mathcal {P}$, where $\mathcal {O}$ is a subalgebra of $\mathcal {W}$, we now spend some time outlining some general facts about subalgebras of the Witt algebra. Note that $\mathcal {W}$ is naturally a $\Bbbk [t,t^{-1}]$-module, and thus, the most natural subalgebras of $\mathcal {W}$ are those which are also $\Bbbk [t,t^{-1}]$-submodules.

#### Definition 4.1

Given $$f \in \Bbbk [t,t^{-1}]$$, define

$$\begin{aligned} f\mathcal {W}:=\{hf\partial \mid h \in \Bbbk [t,t^{-1}]\}, \end{aligned}$$

so that $f\mathcal {W}$ consists of all derivations of $\Bbbk [t,t^{-1}]$ which are divisible by *f*. Given $f \in \Bbbk [t]$, we define $f\mathbb {W}_1$ in a similar way. The Lie subalgebras $f\mathcal {W}$ and $f\mathbb {W}_1$ are known as *submodule-subalgebras* of $\mathcal {W}$ and $\mathbb {W}_1$.

Note that the bracket in $f\mathcal {W}$ is given by:

4.1 $$\begin{aligned} {[}gf\partial ,hf\partial ] = (gh' - g'h)f^2\partial \end{aligned}$$

for all $g,h \in \Bbbk [t,t^{-1}]$. The same formula holds in $f\mathbb {W}_1$, except we require that $f,g,h \in \Bbbk [t]$.

#### Example 4.2

Letting $f :=t^2 - 4$, we define $\mathfrak {f} :=f\mathbb {W}_1$. Then, $\mathfrak {f}$ is spanned by

$$\begin{aligned} f_n :=-t^{n - 1}f\partial = -(t^{n + 1} - 4t^{n - 1})\partial = L_n - 4L_{n - 2}, \end{aligned}$$

where n≥1. The bracket in $\mathfrak {f}$ is

$$\begin{aligned} {[}f_n,f_m]&= [L_n - 4L_{n - 2},L_m - 4L_{m - 2}] \\&= (n - m)(L_{n + m} - 8L_{n + m - 2} + 16L_{n + m - 4}) \\&= (n - m)(f_{n + m} - 4f_{n + m - 2}). \end{aligned}$$

In fact, all subalgebras of $\mathcal {W}$ and $\mathbb {W}_1$ of finite codimension are “essentially” submodule-subalgebras, in a sense that is made precise in the next result.

#### Proposition 4.3

([72, Proposition 3.2.7]) Let $\mathfrak {g}$ be a subalgebra of $\mathcal {W}$ of finite codimension. Then, there exist $f \in \Bbbk [t,t^{-1}] \setminus \{0\}$ and $n \in \mathbb {N}$ such that

$$\begin{aligned} f^n\mathcal {W}\subseteq \mathfrak {g}\subseteq f\mathcal {W}. \end{aligned}$$

The analogous result for finite codimension subalgebras of $\mathbb {W}_1$ is also true with a nearly identical proof, although this is not explicitly proved in [72].

However, the Lie algebra $\mathcal {O}$ is a subalgebra of $\mathcal {W}$ of infinite codimension, so Proposition 4.3 does not apply. Thankfully, there is an analogous classification of subalgebras of $\mathbb {W}_1$ of infinite codimension [7, Theorem 2.8]. Although the result classifies subalgebras of $\mathbb {W}_1$, not of $\mathcal {W}$, the same ideas and techniques can be applied to study subalgebras of $\mathcal {W}$. To state the result, we must first introduce the subalgebras of $\mathbb {W}_1$ which play the role of the submodule-subalgebras in the infinite codimension setting. Following [7, 29], we introduce the notions of the *set* and *field of ratios* associated with a subalgebra of $\mathcal {W}$.

#### Definition 4.4

Let $\mathfrak {g}$ be a Lie subalgebra of $\mathcal {W}$. The *set of ratios* of $\mathfrak {g}$ is

$$\begin{aligned} R(\mathfrak {g}) = \left. \left\{ \frac{g}{h} \in \Bbbk (t) \right| g\partial ,h\partial \in \mathfrak {g}, h \ne 0\right\} . \end{aligned}$$

The *field of ratios* $F(\mathfrak {g})$ of $\mathfrak {g}$ is the subfield of k(t) generated by $R(\mathfrak {g})$.

The next result gives one of the most important reasons why $F(\mathfrak {g})$ is a useful tool to study $\mathfrak {g}$.

#### Lemma 4.5

Let $\mathfrak {g}$ be a subalgebra of $\mathcal {W}$. Then, $\mathfrak {g}\subseteq {{\,\textrm{Der}\,}}(F(\mathfrak {g}))$.

#### Proof

Let $u\partial ,v\partial ,w\partial \in \mathfrak {g}$ with v≠0, where $u,v,w \in \Bbbk [t,t^{-1}]$, so that $\frac{u}{v}, \frac{w}{v} \in R(\mathfrak {g})$. We claim that $w\partial (\frac{u}{v}) \in F(\mathfrak {g})$, which means that elements of $\mathfrak {g}$ map $R(\mathfrak {g})$ to $F(\mathfrak {g})$. Since $F(\mathfrak {g})$ is generated by $R(\mathfrak {g})$ as a field, it will then follow that all derivations in $\mathfrak {g}$ preserve $F(\mathfrak {g})$.

We have

$$\begin{aligned} w\partial \left( \frac{u}{v}\right) = w \cdot \frac{v\partial (u) - u\partial (v)}{v^2} = \frac{w}{v} \cdot \frac{v\partial (u) - u\partial (v)}{v}. \end{aligned}$$

Certainly, $\frac{w}{v} \in R(\mathfrak {g})$ by definition. Note that $[v\partial ,u\partial ] = (v\partial (u) - u\partial (v))\partial \in \mathfrak {g}$, so we also have $\frac{v\partial (u) - u\partial (v)}{v} \in R(\mathfrak {g})$. It follows that $w\partial (\frac{u}{v}) \in F(\mathfrak {g})$, as claimed. □

As the next example illustrates, it is often useful to consider $\mathfrak {g}$ as a subalgebra of ${{\,\textrm{Der}\,}}(F(\mathfrak {g}))$ rather than as a subalgebra of $\mathcal {W}$, at least when $\mathfrak {g}$ has infinite codimension in $\mathcal {W}$.

#### Example 4.6

Consider the subalgebra $\mathfrak {g}= {{\,\textrm{span}\,}}\{L_{2n} \mid n \in \mathbb {Z}\}$. It is easy to see that $\mathfrak {g}$ is a subalgebra of $\mathcal {W}$ of infinite codimension and that $F(\mathfrak {g}) = R(\mathfrak {g}) = \Bbbk (t^2)$, since $\mathfrak {g}= \Bbbk [t^2,t^{-2}]t\partial$. Thus, we see that $\mathfrak {g}$ is the subalgebra ${{\,\textrm{Der}\,}}(\Bbbk [t^2,t^{-2}])$ of ${{\,\textrm{Der}\,}}(\Bbbk (t^2))$. Therefore, when we view $\mathfrak {g}$ as a subalgebra of ${{\,\textrm{Der}\,}}(F(\mathfrak {g}))$, we forget about the basis elements $L_{2n + 1}$ for all $n \in \mathbb {Z}$, which are not relevant when studying Lie algebraic properties of $\mathfrak {g}$.

Since $F(\mathfrak {g}) \subseteq \Bbbk (t)$, Lüroth’s theorem implies that $F(\mathfrak {g}) = \Bbbk (s)$ for some $s \in \Bbbk (t)$, so Lemma 4.5 implies that $\mathfrak {g}\subseteq {{\,\textrm{Der}\,}}(\Bbbk (s))$. In [29, Theorem 5.3], it was shown that *s* can be chosen to be a polynomial when $\mathfrak {g}\subseteq \mathbb {W}_1$. In fact, even more is true: $\mathfrak {g}$ is contained in ${{\,\textrm{Der}\,}}(\Bbbk [s])$ with finite codimension.

#### Theorem 4.7

([7, Theorem 2.8]) Let $\mathfrak {g}$ be an infinite-dimensional subalgebra of $\mathbb {W}_1$. Then, there exists $s \in \Bbbk [t]$ such that $F(\mathfrak {g}) = \Bbbk (s)$, and $\mathfrak {g}$ has finite codimension in ${{\,\textrm{Der}\,}}(\Bbbk [s])$. In particular, there exists $g(s) \in \Bbbk [s]$ such that

$$\begin{aligned} g(s)\Bbbk [s]\partial _s \subseteq \mathfrak {g}\subseteq \Bbbk [s]\partial _s, \end{aligned}$$

where $\partial _s \in {{\,\textrm{Der}\,}}(\Bbbk [s])$ is the unique derivation of k[s] such that $\partial _s(s) = 1$, so that ${{\,\textrm{Der}\,}}(\Bbbk [s]) = \Bbbk [s]\partial _s$.

Let $$\mathfrak {g}\subseteq \mathbb {W}_1$$, $$s \in \Bbbk [t]$$, and $$\partial _s$$ be as in Theorem 4.7. Certainly, the Lie algebra $${{\,\textrm{Der}\,}}(\Bbbk [s])$$ is isomorphic to $$\mathbb {W}_1$$, so Theorem 4.7 says that $$\mathfrak {g}$$ is isomorphic to a subalgebra of $$\mathbb {W}_1$$ of finite codimension. The derivation $$\partial _s \in {{\,\textrm{Der}\,}}(\Bbbk [s])$$ can be uniquely extended to a derivation of k(t): we have $$\partial _s = \frac{1}{s'}\partial $$, where $s^{\prime }:=\partial \left(s\right)$ is the derivative of *s* with respect to *t*. This allows us to embed $${{\,\textrm{Der}\,}}(\Bbbk [s])$$ into $${{\,\textrm{Der}\,}}(\Bbbk (t))$$ as follows:

$$\begin{aligned} {{\,\textrm{Der}\,}}(\Bbbk [s]) = \Bbbk [s]\partial _s = \frac{1}{s'}\Bbbk [s]\partial \subseteq \Bbbk (t)\partial = {{\,\textrm{Der}\,}}(\Bbbk (t)). \end{aligned}$$

Of course, $\mathbb {W}_1 = \Bbbk [t]\partial$ is also contained in ${{\,\textrm{Der}\,}}(\Bbbk (t))$. Therefore, Theorem 4.7 implies that $\mathfrak {g}\subseteq \mathbb {W}_1 \cap {{\,\textrm{Der}\,}}(\Bbbk [s])$, where the intersection is taken in ${{\,\textrm{Der}\,}}(\Bbbk (t))$.

Although Theorem 4.7 only considers subalgebras of $\mathbb {W}_1$, the same ideas can be applied to subalgebras of $\mathcal {W}$. To that end, we introduce the following definition.

#### Notation 4.8

If $s \in \Bbbk [t,t^{-1}]$, we define $L(s) :=\mathcal {W}\cap {{\,\textrm{Der}\,}}(\Bbbk [s])$.

The Lie algebra *L*(*s*) can be described as follows.

#### Lemma 4.9

([7, Lemma 2.7]) Let $s \in \Bbbk [t,t^{-1}]$, and let $g_s \in \Bbbk [t]$ be the unique monic polynomial of minimal degree such that $s'g_s \in \Bbbk [s]$. Letting $f_s \in \Bbbk [t]$ such that $s'g_s = f_s(s)$, we have $L(s) = \Bbbk [s]g_s\partial$.

#### Remark 4.10

Although the condition $s'g_s \in \Bbbk [s]$ might seem unusual, it is precisely the necessary condition for $g_s\partial$ to be a derivation of k[s]. Furthermore, it is not obvious that such a polynomial $g_s$ exists for every $s \in \Bbbk [t,t^{-1}]$; this is proved in [29, Proposition 4.13].

We can also describe *L*(*s*) as a subalgebra of ${{\,\textrm{Der}\,}}(\Bbbk [s]) = \Bbbk [s]\partial _s$ (in other words, viewing it as a Lie algebra of derivations of k[s] instead of $\Bbbk [t,t^{-1}]$). Since $\partial = s'\partial _s$, it follows that

$$\begin{aligned} L(s) = \Bbbk [s]g_s\partial = \Bbbk [s]s'g_s\partial _s = \Bbbk [s]f_s(s)\partial _s. \end{aligned}$$

Therefore, *L*(*s*) is the subalgebra $\Bbbk [s]f_s(s)\partial _s$ of ${{\,\textrm{Der}\,}}(\Bbbk [s])$. By changing variables s↦t, we see that $L(s) \cong \Bbbk [t]f_s\partial = f_s\mathbb {W}_1$, so *L*(*s*) is isomorphic to a subalgebra of $\mathbb {W}_1$ of finite codimension. We now give an example to illustrate the construction.

#### Example 4.11

([29, Example 4.16]) If we let $s = t^3 + 3t$ then $g_s = (t^2 + 1)(t^2 + 4)$ and $f_s = 3(t^2 + 4)$, since $s'g_s = 3(s^2 + 4) \in \Bbbk [s]$. Therefore, $L(s) = \Bbbk [t^3 + 3t](t^2 + 1)(t^2 + 4)\partial$ is a Lie subalgebra of $\mathcal {W}$. As a subalgebra of ${{\,\textrm{Der}\,}}(\Bbbk [s])$, we have $L(s) = \Bbbk [s](s^2 + 4)\partial _s$. Changing variables s↦t, we see that

$$\begin{aligned} L(s) \cong \Bbbk [t](t^2 + 4)\partial = (t^2 + 4)\mathbb {W}_1, \end{aligned}$$

so *L*(*s*) is isomorphic to the subalgebra $(t^2 + 4)\mathbb {W}_1$ of $\mathbb {W}_1$ of codimension 2.

We summarise this discussion below.

#### Proposition 4.12

([29, Lemma 4.12, Proposition 4.13]) Let $s \in \Bbbk [t,t^{-1}]$, and let $f_s, g_s \in \Bbbk [t]$ be as in Lemma 4.9. Then,

$$\begin{aligned} L(s) = \Bbbk [s]g_s\partial = \Bbbk [s]f_s(s)\partial _s \cong f_s\mathbb {W}_1, \end{aligned}$$

where the isomorphism comes from the change of variables s↦t.

### Change of basis for $\mathcal {O}$

We now apply the ideas of Sect. 4.1 to the Lie subalgebra $\mathcal {O}$ of $\mathcal {W}$ to construct a new basis of $\mathcal {O}$, which will allow us to define a descending filtration for the BCCA. In fact, we work in a slightly more general setting: $\mathcal {O}$ is part of a family of Lie subalgebras of $\mathcal {W}$. It is not much more difficult to consider the entire family for the proofs in this section, so we work in this level of generality.

#### Definition 4.13

For $\lambda \in \Bbbk ^*$ and $n \in \mathbb {Z}$, define $\mathcal {O}^{(\lambda )}_n :=L_n - \lambda ^n L_{-n}$. Notice that $\mathcal {O}^{(\lambda )}_0 = 0$ and $\mathcal {O}^{(\lambda )}_{-n} = -\lambda ^{-n}\mathcal {O}^{(\lambda )}_n$ for n≥1. Define $\mathcal {O}(\lambda ) :={{\,\textrm{span}\,}}\{\mathcal {O}^{(\lambda )}_n \mid n \ge 1\}$.

Of course $\mathcal {O}(1) = \mathcal {O}$. On the other hand, it is not immediately clear that $\mathcal {O}(\lambda )$ is closed under the Lie bracket of $\mathcal {W}$ for $\lambda \ne 1$. We prove this next.

#### Lemma 4.14

Let $\lambda \in \Bbbk ^*$. Then, $\mathcal {O}(\lambda )$ is a Lie algebra with the following Lie bracket:

$$\begin{aligned} {[}\mathcal {O}^{(\lambda )}_n,\mathcal {O}^{(\lambda )}_m] = (n - m)\mathcal {O}^{(\lambda )}_{n + m} - \lambda ^m (n + m) \mathcal {O}^{(\lambda )}_{n - m} \end{aligned}$$

for all n,m≥1. In particular, $\mathcal {O}(\lambda )$ is generated by $\mathcal {O}^{(\lambda )}_1$ and $\mathcal {O}^{(\lambda )}_2$.

#### Proof

We compute the bracket $[\mathcal {O}^{(\lambda )}_n,\mathcal {O}^{(\lambda )}_m]$ as follows:

$$\begin{aligned} {[}\mathcal {O}^{(\lambda )}_n,\mathcal {O}^{(\lambda )}_m]&= [L_n - \lambda ^n L_{-n}, L_m - \lambda ^m L_{-m}] \\&= (n - m)L_{n + m} - \lambda ^m (n + m) L_{n - m} \\&\quad + \lambda ^n (n + m) L_{m - n} - \lambda ^{n + m}(n - m)L_{-(n + m)} \\&= (n - m)\mathcal {O}^{(\lambda )}_{n + m} - \lambda ^m (n + m)\mathcal {O}^{(\lambda )}_{n - m}. \end{aligned}$$

The final sentence follows by an easy induction. □

#### Remark 4.15

One might ask if we can get different subalgebras of the Witt algebra by combining $L_n$ and $L_{-n}$ differently to the way it is done in $\mathcal {O}(\lambda )$. In other words, can we generate different Lie subalgebras of $\mathcal {W}$ with elements $L_1 - \lambda L_{-1}$ and $L_2 - \mu L_{-2}$, where $\lambda , \mu \in \Bbbk ^*$? This question is answered in Appendix A: if $\mu \ne \lambda ^2$, then these two elements generate the entire Witt algebra. As a result, the subalgebras $\mathcal {O}(\lambda )$ are the unique family of proper subalgebras of $\mathcal {W}$ generated by elements of the form $L_1 - \lambda L_{-1}$ and $L_2 - \mu L_{-2}$ for some $\lambda , \mu \in \Bbbk ^*$.

We now aim to describe an alternative basis $u_n^{(\lambda )}$ for $\mathcal {O}(\lambda )$. To achieve this, we analyse the structure of $\mathcal {O}(\lambda )$ following the methods of Sect. 4.1. In terms of polynomials, we have $\mathcal {O}^{(\lambda )}_1 = -(t^2 - \lambda )\partial$, and

$$\begin{aligned} \mathcal {O}^{(\lambda )}_2 = -(t^3 - \lambda ^2 t^{-1})\partial = -t^{-1}(t^4 - \lambda ^2)\partial = -t^{-1}(t^2 - \lambda )(t^2 + \lambda )\partial . \end{aligned}$$

Dividing $\mathcal {O}^{(\lambda )}_2$ by $\mathcal {O}^{(\lambda )}_1$, it follows that

$$\begin{aligned} \frac{t^{-1}(t^2 - \lambda )(t^2 + \lambda )}{t^2 - \lambda } = t + \lambda t^{-1} \in R(\mathcal {O}(\lambda )). \end{aligned}$$

We therefore define $s_\lambda :=t + \lambda t^{-1}$, so that $\Bbbk (s_\lambda ) \subseteq F(\mathcal {O}(\lambda ))$. In fact, as we show next, we have $R(\mathcal {O}(\lambda )) = F(\mathcal {O}(\lambda )) = \Bbbk (s_\lambda )$ and $\mathcal {O}(\lambda ) = L(s_\lambda )$, where this notation is defined in Definition 4.4 and Notation 4.8.

#### Theorem 4.16

Let $\lambda \in \Bbbk ^*$, and define $\mathfrak {f}_\lambda :=f_{s_\lambda } \mathbb {W}_1$, where $f_{s_\lambda } = t^2 - 4\lambda$. Then,

$$\begin{aligned} \mathcal {O}(\lambda ) = L(s_\lambda ) = \Bbbk [t + \lambda t^{-1}](t^2 - \lambda )\partial = \Bbbk [s_\lambda ](s_\lambda ^2 - 4\lambda )\partial _{s_\lambda } \cong \mathfrak {f}_\lambda , \end{aligned}$$

where the isomorphism comes from the change of variables $s_\lambda \mapsto t$. Consequently, $\mathcal {O}(\lambda )$ has another basis $\{u^{(\lambda )}_n \mid n \ge 1\}$ defined as follows: for n≥1, let

$$\begin{aligned} u^{(\lambda )}_n :=-s_\lambda ^{n - 1}(s_\lambda ^2 - 4\lambda )\partial _{s_\lambda } = -(t + \lambda t^{-1})^{n - 1}(t^2 - \lambda )\partial . \end{aligned}$$

The Lie bracket of $\mathcal {O}(\lambda )$ with this basis is given by

$$\begin{aligned} {[}u^{(\lambda )}_n,u^{(\lambda )}_m] = (n - m)(u^{(\lambda )}_{n + m} - 4\lambda u^{(\lambda )}_{n + m - 2}) \end{aligned}$$

for all n,m≥1.

#### Proof

We adopt the notation $g_{s_\lambda }$ and $f_{s_\lambda }$ from Lemma 4.9. It is not difficult to see that $g_{s_\lambda } = t^2 - \lambda$, since

$$\begin{aligned} s_\lambda '(t^2 - \lambda ) = (1 - \lambda t^{-2})(t^2 - \lambda ) = t^2 - 2\lambda + \lambda ^2 t^{-2} = (t + \lambda t^{-1})^2 - 4\lambda = s_\lambda ^2 - 4\lambda \in \Bbbk [s_\lambda ]. \end{aligned}$$

Consequently, $f_{s_\lambda } = t^2 - 4\lambda$. It follows from Proposition 4.12 that

$$\begin{aligned} L(s_\lambda ) = \Bbbk [s_\lambda ](s_\lambda ^2 - 4\lambda )\partial _{s_\lambda } = \Bbbk [s_\lambda ]g_{s_\lambda }\partial = \Bbbk [t + \lambda t^{-1}](t^2 - \lambda )\partial . \end{aligned}$$

Note that $\mathcal {O}^{(\lambda )}_1 = -(t^2 - \lambda )\partial \in L(s_\lambda )$ and

$$\begin{aligned} \mathcal {O}^{(\lambda )}_2 = -(t^3 - \lambda ^2 t^{-1})\partial = -(t + \lambda t^{-1})(t^2 - \lambda )\partial \in L(s_\lambda ). \end{aligned}$$

Since $\mathcal {O}^{(\lambda )}_1$ and $\mathcal {O}^{(\lambda )}_2$ generate $\mathcal {O}(\lambda )$ by Lemma 4.14, this implies that $\mathcal {O}(\lambda ) \subseteq L(s_\lambda )$.

For the other inclusion $L(s_\lambda ) \subseteq \mathcal {O}(\lambda )$, we will show that $L(s_\lambda )$ is also generated by $\mathcal {O}^{(\lambda )}_1$ and $\mathcal {O}^{(\lambda )}_2$. To that end, noting that $\{u^{(\lambda )}_n \mid n \ge 1\}$ is a basis for $L(s_\lambda )$, we compute the bracket $[u^{(\lambda )}_n,u^{(\lambda )}_m]$. Letting $\mathfrak {f}_\lambda :=f_{s_\lambda }\mathbb {W}_1 = (t^2 - 4\lambda )\mathbb {W}_1$, the change of variables $s_\lambda \mapsto t$ yields an isomorphism $L(s_\lambda ) \cong \mathfrak {f}_\lambda$, as in Proposition 4.12. Noting that

$$\begin{aligned} u^{(\lambda )}_n = -(t + \lambda t^{-1})^{n - 1}(t^2 - \lambda )\partial = -s_\lambda ^{n - 1}(s_\lambda ^2 - 4\lambda )\partial _{s_\lambda }, \end{aligned}$$

we see that

$$\begin{aligned} u^{(\lambda )}_n \mapsto f^{(\lambda )}_n :=-t^{n - 1} f_\lambda \partial = L_n - 4\lambda L_{n - 2} \end{aligned}$$

under the change of variables $s_\lambda \mapsto t$. We have

$$\begin{aligned} {[}f^{(\lambda )}_n,f^{(\lambda )}_m]&= [L_n - 4\lambda L_{n - 2},L_m - 4\lambda L_{m - 2}] \\&= (n - m)(L_{n + m} - 8\lambda L_{n + m - 2} + 16\lambda ^2 L_{n + m - 4}) \\&= (n - m)(f^{(\lambda )}_{n + m} - 4 \lambda f^{(\lambda )}_{n + m -2}), \end{aligned}$$

and therefore,

$$\begin{aligned} {[}u^{(\lambda )}_n,u^{(\lambda )}_m] = (n - m)(u^{(\lambda )}_{n + m} - 4\lambda u^{(\lambda )}_{n + m - 2}). \end{aligned}$$

An easy induction shows that $\mathcal {O}^{(\lambda )}_1 = u^{(\lambda )}_1$ and $\mathcal {O}^{(\lambda )}_2 = u^{(\lambda )}_2$ generate $L(s_\lambda )$, which concludes the proof. □

#### Remark 4.17

Theorem 4.16 implies that $R(\mathcal {O}(\lambda )) = F(\mathcal {O}(\lambda )) = \Bbbk (s_\lambda )$, although a priori it is not obvious that $R(\mathcal {O}(\lambda ))$ is a field.

#### Example 4.18

The Lie algebra $\mathcal {O}(-1)$ is spanned by $L_n + (-1)^{n + 1}L_{-n}$ and is isomorphic to $(t^2 + 4)\mathbb {W}_1$.

Using Theorem 4.16, it is now easy to see that $\mathcal {O}(\lambda )$ is never simple.

#### Corollary 4.19

For all $\lambda \in \Bbbk ^*$, the Lie algebra $\mathcal {O}(\lambda )$ is not perfect and therefore is not simple.

#### Proof

The Lie algebra $\mathfrak {f}_\lambda$ is not perfect: this is because the derived subalgebra of $f^{(\lambda )}$ is

$$\begin{aligned} {[}\mathfrak {f}_\lambda ,\mathfrak {f}_\lambda ] = [f_{s_\lambda }\mathbb {W}_1,f_{s_\lambda }\mathbb {W}_1] = f_{s_\lambda }^2\mathbb {W}_1, \end{aligned}$$

by (4.1). The result now follows by the isomorphism $\mathcal {O}(\lambda ) \cong \mathfrak {f}_\lambda$ from Theorem 4.16. □

#### Remark 4.20

Corollary 4.19 implies that $\mathfrak {b}$ is not perfect, meaning it does not have a universal central extension [83, Theorem 7.9.2]. Nonetheless, it would be interesting to compute the *centrally closed* version of $\mathfrak {b}$, which would be the central extension of $\mathfrak {b}$ with trivial second cohomology group (i.e. does not admit any more non-trivial central extensions). From a physics perspective, this would tell us the possible “central charges” that a quantum field theory with $\mathfrak {b}$ symmetry could admit. It remains to be seen whether this centrally closed Lie algebra is indeed $\widehat{\mathfrak {b}}$.

We highlight that the computation of central extensions of $\mathfrak {b}$ is mathematically nontrivial, mainly due to the fact that $\mathfrak {b}$ is not graded. There are many results that greatly simplify the computation of central extensions of graded Lie algebras, such as [49, Theorem 1.5.2]. On the other hand, the centreless BCCA is a semi-direct sum Lie algebra, which can be studied using techniques developed in [52, Proposition 1] and [31, Proposition 3.1]. While these results would certainly help when computing the central extensions of $\mathfrak {b}$, we expect this to be a challenging task. See [77] for an example of computing central extensions of non-graded Lie algebras.

As we aim to show next, the Lie algebras $\mathcal {O}(\lambda )$ are often isomorphic to each other for different values of $\lambda \in \Bbbk ^*$. Recalling that $\mathcal {O}(\lambda ) \cong \mathfrak {f}_\lambda = (t^2 - 4\lambda )\mathbb {W}_1$, it suffices to consider when two subalgebras of $\mathbb {W}_1$ of finite codimension are isomorphic. In fact, this can only happen if the isomorphism is induced by an automorphism of $\mathbb {W}_1$ [30]. The automorphisms of $\mathbb {W}_1$ are well-known [76].

#### Theorem 4.21

([30, Theorem 4.1 and Corollary 4.12]) Let $\mathfrak {g}$ and $\mathfrak {h}$ be subalgebras of $\mathbb {W}_1$ of finite codimension and suppose there is an isomorphism $\varphi : \mathfrak {g}\rightarrow \mathfrak {h}$. Then φ extends to an automorphism of $\mathbb {W}_1$. In particular, $g\mathbb {W}_1 \cong h\mathbb {W}_1$ if and only if there exist $\alpha , \gamma \in \Bbbk ^*$ and $\beta \in \Bbbk$ such that g(z)=γh(t), where $z = \alpha t + \beta$ and $g,h \in \Bbbk [t]$.

We are now ready to characterise when $\mathcal {O}(\lambda ) \cong \mathcal {O}(\mu )$.

#### Proposition 4.22

Let $\lambda , \mu \in \Bbbk ^*$. Then $\mathcal {O}(\lambda ) \cong \mathcal {O}(\mu )$ if and only if $\sqrt{\frac{\lambda }{\mu }} \in \Bbbk$.

#### Proof

By Theorem 4.16, we have $\mathcal {O}(\lambda ) \cong \mathfrak {f}_\lambda = (t^2 - 4\lambda )\mathbb {W}_1$ and $\mathcal {O}(\mu ) \cong \mathfrak {f}_\mu = (t^2 - 4\mu )\mathbb {W}_1$. Therefore, we will prove the result by showing that $\mathfrak {f}_\lambda \cong \mathfrak {f}_\mu$ if and only if $\sqrt{\frac{\mu }{\lambda }} \in \Bbbk$.

First, assume that $\alpha :=\sqrt{\frac{\lambda }{\mu }} \in \Bbbk$. Letting z:=αt, we have

$$\begin{aligned} f_{s_\lambda }(z) = z^2 - 4\lambda = \alpha ^2 t^2 - 4\lambda = \frac{\lambda }{\mu } t^2 - 4\lambda = \frac{\lambda }{\mu }(t^2 - 4\mu ) = \frac{\lambda }{\mu } f_{s_\mu }(t). \end{aligned}$$

By Theorem 4.21, the change of variables $t \mapsto \alpha t$ induces an isomorphism $\mathfrak {f}_\lambda \cong \mathfrak {f}_\mu$.

Conversely, assume that $\mathfrak {f}_\lambda \cong \mathfrak {f}_\mu$. By Theorem 4.21, there exist $\alpha , \gamma \in \Bbbk ^*$ and $\beta \in \Bbbk$ such that $f_{s_\lambda }(z) = \gamma f_{s_\mu }(t)$, where $z = \alpha t + \beta$. Therefore,

$$\begin{aligned} t^2 - 4\mu&= f_{s_\mu }(t) = \frac{1}{\gamma } f_{s_\lambda }(z) = \frac{1}{\gamma }(z^2 - 4\lambda ) = \frac{1}{\gamma }\Big ((\alpha t + \beta )^2 - 4\lambda \Big ) \\&= \frac{1}{\gamma }(\alpha ^2 t^2 + 2 \alpha \beta t + \beta ^2 - 4\lambda ). \end{aligned}$$

It follows that $\gamma = \alpha ^2$, β=0, and $\lambda = \gamma \mu = \alpha ^2 \mu$. Therefore, $\alpha = \sqrt{\frac{\lambda }{\mu }}$ must be an element of k. □

With $\mathcal {O}(1) = \mathcal {O}$, the next result follows immediately from Proposition 4.22.

#### Corollary 4.23

If k is algebraically closed (for example, if $\Bbbk = \mathbb {C}$), then $\mathcal {O}(\lambda ) \cong \mathcal {O}$ for all $\lambda \in \Bbbk$. If $\Bbbk = \mathbb {R}$, then

$$\mathcal {O}(\lambda ) \cong {\left\{ \begin{array}{ll} \mathcal {O}, & {\text {if }} \lambda > 0, \\ \mathcal {O}(-1), & {\text {if }} \lambda < 0, \end{array}\right. }$$

but $$\mathcal {O}(-1) \not \cong \mathcal {O}$$. □

#### Remark 4.24

Corollary 4.23 is intriguing in the context in which the BCCA was discovered [12]. Specifically, we have shown that the subalgebras $\mathcal {O}= {{\,\textrm{span}\,}}\{L_n - L_{-n} \mid n \ge 1\}$ and $\mathcal {O}(-1) = {{\,\textrm{span}\,}}\{L_n + (-1)^{n + 1} L_{-n}\}$ are isomorphic as complex Lie algebras but not as real ones. In the realisation of the BMS_3_ algebra given by [12, Equation (4)] in terms of vector fields which preserve the Carrollian structure on cylinder (see [38, Section 2.1]), we have

$$\begin{aligned} L_n = e^{in\sigma }(\partial _\sigma + in \tau \partial _\tau ), \end{aligned}$$

where σ and τ are the coordinates of a cylinder. The Lie algebra $\mathcal {O}$ was constructed as the subalgebra of the Witt algebra spanned by $\{L_n \mid n \in \mathbb {Z}\}$, which leaves the choice of boundary conditions $\sigma = 0, \pi$ invariant [12, Equation (5)]

$$\begin{aligned} \mathcal {O}_n = L_n - L_{-n} = 2i \sin (n\sigma )\partial _\sigma + 2in \tau \cos (n\sigma )\partial _\tau . \end{aligned}$$

At $\sigma = 0,\pi$, the vector fields $\mathcal {O}_n = \pm 2in \tau \partial _\tau$ do not contain any nonzero $\partial _\sigma$ terms and thus satisfy the boundary conditions.

In terms of vector fields on the cylinder, the algebra $\mathcal {O}(-1)$ preserves a different choice of boundaries, in this case at $\sigma = \frac{\pi }{2}, \frac{3\pi }{2}$. This can be seen as follows:

$$\begin{aligned} \mathcal {O}_{2n}^{(-1)}&= L_{2n} - L_{-2n} = 2i \sin (2n\sigma )\partial _\sigma + 4in \tau \cos (2n\sigma ) \partial _\tau , \\ \mathcal {O}_{2n + 1}^{(-1)}&= L_{2n + 1} + L_{-2n - 1} = 2\cos ((2n + 1)\sigma )\partial _\sigma - (4n + 2) \tau \sin ((2n + 1)\sigma )\partial _\tau . \end{aligned}$$

Therefore, at $\sigma = \frac{\pi }{2}, \frac{3\pi }{2}$ we have $\mathcal {O}_{2n}^{(-1)} = \pm 4in \tau \partial _\tau$ and $\mathcal {O}_{2n + 1}^{(-1)} = \pm (4n + 2) \tau \partial _\tau$, which satisfy the boundary conditions.

When working over $$\mathbb {C}$$, this can be undone by a simple change of coordinates to $$\sigma ' = \sigma - \frac{\pi }{2}$$, with the resulting boundary conditions in terms of $\sigma^{\prime }$ being equivalent to the original one. Consequently, one would expect to have recovered $$\mathcal {O}$$. Indeed, this change of coordinates is just a rescaling of $$\mathcal {O}$$ by *i*, which reiterates the fact that the authors of the original paper were working over $$\mathbb {C}$$. However, Corollary 4.23 implies that the boundary conditions at $$\sigma = \frac{\pi }{2}, \frac{3\pi }{2}$$ result in a non-isomorphic Lie algebra when working over $$\mathbb {R}$$.

We now focus on the case of $\mathcal {O}= \mathcal {O}(1)$.

#### Notation 4.25

We make the following definitions:

$$\begin{aligned} s&:=s_1 = t + t^{-1}, \\ u_n&:=u_n^{(1)} = -(t + t^{-1})^{n - 1}(t^2 - \lambda )\partial = -s^{n - 1}(s^2 - 4)\partial _s, \\ f&:=f_{s_1} = t^2 - 4, \\ f_n&:=f_n^{(1)} = -t^{n - 1}f\partial = L_n - 4L_{n - 2}, \\ \mathfrak {f}&:=\mathfrak {f}_1 = f\mathbb {W}_1 = (t^2 - 4)\mathbb {W}_1, \end{aligned}$$

where $$n \in \mathbb {Z}_+$$.

Of course, $\{\mathcal {O}_n \mid n \ge 1\}$ and $\{u_n \mid n \ge 1\}$ are two bases for the same Lie algebra $\mathcal {O}$, so it is natural to ask how they are related. The following result answers this.

#### Lemma 4.26

We have

$$\begin{aligned} u_n = \mathcal {O}_n + \sum _{k = 1}^{\left\lfloor \frac{n}{2} \right\rfloor } \left( {\begin{array}{c}n\\ k\end{array}}\right) \left( 1 - \frac{2k}{n}\right) \mathcal {O}_{n - 2k} \end{aligned}$$

for all n≥1.

#### Proof

First, note that $u_1 = -(t^2 - 1)\partial = L_1 - L_{-1} = \mathcal {O}_1$. Furthermore,

4.2 $$\begin{aligned} \begin{aligned} -(t^n + t^{-n})(t^2 - 1)\partial&= -\Big (t^{n + 2} - t^n + t^{-n + 2} - t^{-n}\Big )\partial \\&= L_{n + 1} - L_{n - 1} + L_{-n + 1} - L_{-n - 1} \\&= (L_{n + 1} - L_{-(n + 1)}) - (L_{n - 1} - L_{-(n - 1)}) \\&= \mathcal {O}_{n + 1} - \mathcal {O}_{n - 1}. \end{aligned} \end{aligned}$$

We now compute for n≥2:

$$\begin{aligned} u_{n} = -(t + t^{-1})^{n - 1}(t^2 - 1)\partial = -\left( \sum _{k = 0}^{n - 1} \left( {\begin{array}{c}n - 1\\ k\end{array}}\right) t^{n - 2k-1}\right) (t^2 - 1)\partial . \end{aligned}$$

Pairing up $t^m$ with $t^{-m}$ and using (4.2), we get

$$\begin{aligned} u_n = \mathcal {O}_n + \sum _{k = 1}^{\left\lfloor \frac{n}{2} \right\rfloor } \left( \left( {\begin{array}{c}n - 1\\ k\end{array}}\right) - \left( {\begin{array}{c}n - 1\\ k - 1\end{array}}\right) \right) \mathcal {O}_{n - 2k}. \end{aligned}$$

Applying the identity

$$\begin{aligned} \left( {\begin{array}{c}n - 1\\ k\end{array}}\right) - \left( {\begin{array}{c}n - 1\\ k - 1\end{array}}\right) = \left( {\begin{array}{c}n\\ k\end{array}}\right) \left( 1 - \frac{2k}{n}\right) , \end{aligned}$$

the result now follows. □

### Change of basis for $\mathcal {P}_b$

In Theorem 4.16, it was shown that the change of variables s↦t yields the isomorphism $\mathcal {O}\cong \mathfrak {f}= \Bbbk [t](t^2 - 4)\partial$. Therefore, it is natural to ask if this change of variables gives a similar description of $\mathcal {P}$. In this section, we show that this is indeed the case: the change of variables will allow us to define a new basis for $\mathcal {P}$. We will work in a slightly more general setting: we consider the change of variables on the entire family of $\mathcal {O}$-modules $\mathcal {P}_b$ for arbitrary $b \in \Bbbk$ (see Definition 2.6). Just like for $\mathcal {O}$, it will be useful to have a generating set for the $\mathcal {O}$-module $\mathcal {P}_b$.

#### Lemma 4.27

For all $b \in \Bbbk ^*$, the $\mathcal {O}$-module $\mathcal {P}_b$ is generated by $P^{(b)}_0$. For b=0, the $\mathcal {O}$-module $\mathcal {P}_0$ is generated by $P_0^{(0)}$ and $P_1^{(0)}$.

#### Proof

If b≠0, this follows immediately (2.4), since

$$\begin{aligned} \mathcal {O}_n \cdot P^{(b)}_0 = -2bn P^{(b)}_n \end{aligned}$$

for all n≥1. If b=0, then (2.4) gives

$$\begin{aligned} \mathcal {O}_1 \cdot P_n^{(0)} = n (P_{n + 1}^{(0)} - P_{n - 1}^{(0)}) \end{aligned}$$

for all $n \in \mathbb {N}$. The result now follows by an easy induction. □

To describe how the change of variables s↦t affects $\mathcal {P}_b$, we first introduce some notation. As above, we make the identification $\partial _s = \frac{1}{s'}\partial$, so that $\partial _s(s) = 1$. Letting $r :=t - t^{-1}$, we have

4.3 $$\begin{aligned} \partial _s = \frac{1}{s'}\partial = \frac{1}{1 - t^{-2}}\partial = \frac{t}{t - t^{-1}}\partial = \frac{t}{r}\partial . \end{aligned}$$

Note that

4.4 $$\begin{aligned} r^2 = (t - t^{-1})^2 = t^2 - 2 + t^{-2} = (t + t^{-1})^2 - 4 = s^2 - 4. \end{aligned}$$

In other words, *r* is a square root of $s^2 - 4$.

Recall that $\mathcal {P}_b$ is the $\mathcal {O}$-submodule of *I*(0, *b*) spanned by $P^{(b)}_n = I_n + I_{-n}$. As mentioned in Definition 2.2, we view *I*(0, *b*) as $I(0,b) = t^{-b}\Bbbk [t,t^{-1}] dt^b$ with $I_n = t^{n - b} \ dt^b$. With this perspective, we have

4.5 $$\begin{aligned} P^{(b)}_n = I_n + I_{-n} = -(t^n + t^{-n})t^{-b} \ dt^b. \end{aligned}$$

Now, we want to express $$\mathcal {P}_b$$ in terms of polynomials in *s*, just like we did with $$\mathcal {O}$$. By definition of the Kähler differential, we have $ds=s^{\prime }\;dt$. It follows that $$ds^b = (s' \ dt)^b = (s')^b \ dt^b$$, and therefore,

4.6 $$\begin{aligned} dt^b = (s')^{-b} \ ds^b = (1 - t^{-2})^{-b} \ ds^b = \frac{t^b}{r^b} \ ds^b, \end{aligned}$$

so $t^{-b} \ dt^b = r^{-b} \ ds^b$. The next result shows what happens to the generators of $\mathcal {P}_b$ when written in terms of $ds^b$.

#### Lemma 4.28

For $b \in \Bbbk$, we have $P^{(b)}_0 = -2r^{-b} \ ds^b$ and $P^{(b)}_1 = -sr^{-b} \ ds^b$.

#### Proof

Follows immediately from (4.5) and (4.6) upon recalling that $s = t + t^{-1}$. □

Inspired by Lemma 4.28, we now introduce the following notation.

#### Notation 4.29

For $b \in \Bbbk$ and $n \in \mathbb {N}$, we define $v_n^{(b)} :=-s^n r^{-b} \ ds^b = -(t + t^{-1})^n t^{-b} \ dt^b \in I(0,b)$.

By Lemma 4.28, we have $v_0^{(b)} = \frac{1}{2}P^{(b)}_0$ and $v_1^{(b)} = P^{(b)}_1$. In fact, as we show next, $\{v_n^{(b)} \mid n \in \mathbb {N}\}$ is another basis for $\mathcal {P}_b$.

#### Theorem 4.30

For all $b \in \Bbbk$, we have

$$\begin{aligned} \mathcal {P}_b = {{\,\textrm{span}\,}}\{v_n^{(b)} \mid n \in \mathbb {N}\} = \Bbbk [s]r^{-b} \ ds^b = \Bbbk [t + t^{-1}] t^{-b} \ dt^b. \end{aligned}$$

The $\mathcal {O}$-action on $\mathcal {P}_b$ with this basis is given by

$$\begin{aligned} u_n \cdot v_m^{(b)} = -(bn + m)v_{n + m}^{(b)} + 4(b(n - 1) + m)v_{n + m - 2}^{(b)} \end{aligned}$$

for all n≥1 and m≥0. In basis-free notation, the $\mathcal {O}$-action is

4.7 $$\begin{aligned} (s^2 - 4)g\partial _s \cdot (hr^{-b} \ ds^b) = \Big ((g\partial _s(h) + b \partial _s(g)h)(s^2 - 4) + b ghs\Big )r^{-b} \ ds^b \end{aligned}$$

for all $$g,h \in \Bbbk [s]$$.

#### Proof

We start by computing $u_n \cdot v_m^{(b)}$. To that end, note that

$$\begin{aligned} \partial _s(r) = \frac{r'}{s'} = \frac{(t - t^{-1})'}{(t + t^{-1})'} = \frac{1 + t^{-2}}{1 - t^{-2}} = \frac{t + t^{-1}}{t - t^{-1}} = \frac{s}{r}, \end{aligned}$$

and thus

$$\begin{aligned} \partial _s(r^{-b}) = -b r^{-b - 1} \partial _s(r) = \frac{-b s}{r^{b + 2}} = \frac{-bsr^{-b}}{s^2 - 4}, \end{aligned}$$

where we used that $r^2 = s^2 - 4$ from (4.4) in the last equality. Therefore, we have

$$\begin{aligned} (s^2 - 4)g\partial _s&\cdot (hr^{-b} \ ds^b) = \Big ((s^2 - 4) g \partial _s(hr^{-b}) + b\partial _s((s^2 - 4)g)hr^{-b}\Big )ds^b \\&= \Big ((s^2 - 4) g \big (\partial _s(h)r^{-b} + h\partial _s(r^{-b})\big ) + b\big (\partial _s(s^2 - 4)g + (s^2 - 4)\partial _s(g)\big )hr^{-b}\Big )ds^b \\&= \left( (s^2 - 4) g \left( \partial _s(h)r^{-b} - \frac{bhsr^{-b}}{s^2 - 4}\right) + b\big (2sg + (s^2 - 4)\partial _s(g)\big )hr^{-b}\right) ds^b \\&= \Big (\big (g \partial _s(h) + b\partial _s(g)h\big )(s^2 - 4) + b ghs\Big )r^{-b} \ ds^b, \end{aligned}$$

as required. It follows that

$$\begin{aligned} u_n \cdot v_m^{(b)}&= [s^{n - 1}(s^2 - 4)\partial _s,s^m r^{-b} \ ds^b] \\&= \Big (\big (s^{n - 1}\partial _s(s^m) + b\partial _s(s^{n - 1})s^m\big )(s^2 - 4) + b s^{n + m}\Big )r^{-b} \ ds^b \\&= \Big ((m + b(n - 1)) s^{n + m - 2} (s^2 - 4) + b s^{n + m}\Big )r^{-b} \ ds^b \\&= \Big ((m + b n)s^{n + m} - 4(b(n - 1) - m)s^{n + m - 2}\Big )r^{-b} \ ds^b \\&= (n - m)v_{n + m}^{(b)} + 4(b(n - 1) - m)v_{n + m - 2}^{(b)}. \end{aligned}$$

Thus, letting $V :={{\,\textrm{span}\,}}\{v_n^{(b)} \mid n \in \mathbb {N}\} = \Bbbk [s]r^{-b} \ ds^b$, we see that *V* is an $\mathcal {O}$-module. Since $P^{(b)}_0 = 2v_0^{(b)}$ and $P^{(b)}_1 = v_1^{(b)}$ are elements of *V*, Lemma 4.27 implies that $\mathcal {P}_b \subseteq V$.

For the other inclusion $V \subseteq \mathcal {P}_b$, it suffices to show that $v_0^{(b)}$ and $v_1^{(b)}$ generate *V*. We have

$$\begin{aligned} u_n \cdot v_0^{(b)}&= -bn v_n^{(b)} + 4b(n - 1) v_{n - 2}^{(b)} \\ u_1 \cdot v_n^{(b)}&= -(m + b)v_{n + 1}^{(b)} + 4m v_{n - 1}^{(b)} \end{aligned}$$

for all *n*. The result now follows by an easy induction. □

The next result, which shows that $\mathcal {P}_b$ is a reducible $\mathcal {O}$-module for all $b \in \Bbbk$, follows immediately from Theorem 4.30.

#### Corollary 4.31

Let $b \in \Bbbk$. Then $(s + 2)\mathcal {P}_b$ and $(s - 2)\mathcal {P}_b$ are proper $\mathcal {O}$-submodules of $\mathcal {P}_b$. Consequently, $\mathcal {P}_b$ is a reducible $\mathcal {O}$-module for all $b \in \Bbbk$.

#### Proof

If *h* is a multiple of s±2 in (4.7), then $[(s^2 - 4)g\partial _s, hr \ ds^{-1}]$ is also a multiple of s±2 for all $g \in \Bbbk [s]$, so we see that $(s \pm 2)\mathcal {P}_b$ is an $\mathcal {O}$-submodule of $\mathcal {P}_b$. □

On the other hand, $\mathcal {P}_b$ is indecomposable as an $\mathcal {O}$-module, provided b≠0.

#### Lemma 4.32

If $b \in \Bbbk \setminus \{0\}$, then $\mathcal {P}_b$ is an indecomposable $\mathcal {O}$-module. Furthermore, $\mathcal {P}_0$ decomposes as

$$\begin{aligned} \mathcal {P}_0 = \Bbbk P_0^{(0)} \oplus (s + 2)\mathcal {P}_0 = \Bbbk P_0^{(0)} \oplus (s - 2)\mathcal {P}_0. \end{aligned}$$

#### Proof

The first part of the result follows from the following easy claim: if *M* is a nonzero submodule of $\mathcal {P}_b$ with b≠0, then *M* has finite codimension in $\mathcal {P}_b$. The second part is immediate from (2.4) and Corollary 4.31. □

We finish by considering some consequences of Theorem 4.30 for $\mathcal {P}= \mathcal {P}_{-1}$. First, we simplify the notation for the new basis of $\mathcal {P}$, since this is the main $\mathcal {O}$-module of interest in this paper.

#### Notation 4.33

We define $v_n :=v_n^{(-1)} = -s^n r \ ds^{-1} = -(t + t^{-1})^n t \ dt^{-1} \in \mathcal {P}$ for $n \in \mathbb {N}$.

The next result computes the bracket of the centreless BCCA $\mathfrak {b}$ with the new bases for $\mathcal {O}$ and $\mathcal {P}$.

#### Corollary 4.34

We have $\mathfrak {b}= {{\,\textrm{span}\,}}\{u_n, v_m \mid n \ge 1, m \in \mathbb {N}\}$. The bracket of $\mathfrak {b}$ with this basis is given by:

$$\begin{aligned} {[}u_n,u_m]&= (n - m)(u_{n + m} - 4u_{n + m - 2})  &   (n,m \ge 1), \\ {[}u_n,v_m]&= (n - m)v_{n + m} - 4(n - m - 1)v_{n + m - 2}  &   (n \ge 1, m \ge 0), \\ {[}v_n,v_m]&= 0  &   (n,m \ge 0). \end{aligned}$$

#### Proof

Follows immediately from Theorems 4.16 and 4.30. □

As was done in Lemma 4.26, we consider the relationship between the two bases $P_n$ and $v_n$ of $\mathcal {P}$. Since $P_0 = 2v_0$, we need to be a bit careful, but otherwise the proof is nearly identical to that of Lemma 4.26.

#### Lemma 4.35

For all $n \in \mathbb {N}$, we have

$$\begin{aligned} v_n = Q_n + \sum _{k = 1}^{\lfloor \frac{n}{2} \rfloor } \left( {\begin{array}{c}n\\ k\end{array}}\right) Q_{n - 2k}, \end{aligned}$$

where $$Q_n :={\left\{ \begin{array}{ll} P_n, & {\text {if }} n \ne 0, \\ \frac{1}{2}P_0, & {\text {if }} n = 0. \end{array}\right. }$$

#### Proof

Similar to Lemma 4.26, except we apply the identity

$$\begin{aligned} \left( {\begin{array}{c}n - 1\\ k\end{array}}\right) + \left( {\begin{array}{c}n - 1\\ k - 1\end{array}}\right) = \left( {\begin{array}{c}n\\ k\end{array}}\right) \end{aligned}$$

in the last part of the proof. □

Thanks to Lemmas 4.26 and 4.35, we can easily deduce what the central extension of $\mathfrak {b}$ giving rise to $\widehat{\mathfrak {b}}$ looks like using the new basis $\{u_n, v_m \mid n \ge 1, m \ge 0\}$ of $\mathfrak {b}$. As the next result shows, the central extension becomes rather unwieldy with this choice of basis.

#### Corollary 4.36

View $u_n$ and $v_n$ as elements of the (centrally extended) BCCA $\widehat{\mathfrak {b}}$. Then

$$\begin{aligned} {[}u_{2n},v_{2m + 1}]&= (2(n - m) - 1)v_{2(n + m) + 1} - 8(n - m - 1)v_{2(n + m) - 1} \\ {[}u_{2n + 1},v_{2m}]&= (2(n - m) + 1)v_{2(n + m) + 1} - 8(n - m)v_{2(n + m) - 1} \\ {[}u_{2n},v_{2m}]&= 2(n - m)v_{2(n + m)} - 4(2(n - m) - 1)v_{2(n + m - 1)} \\&\quad + \left( \sum _{k = 0}^{\min (n,m)} \left( {\begin{array}{c}2n\\ n - k\end{array}}\right) \left( {\begin{array}{c}2m\\ m - k\end{array}}\right) \frac{k^2 (4k^2 - 1)}{3n}\right) C_M \\ {[}u_{2n + 1},v_{2m + 1}]&= 2(n - m)v_{2(n + m + 1)} - 4(2(n - m) - 1)v_{2(n + m)} \\&\quad + \left( \sum _{k = 0}^{\min (n,m)} \left( {\begin{array}{c}2n + 1\\ n - k\end{array}}\right) \left( {\begin{array}{c}2m + 1\\ m - k\end{array}}\right) \frac{2 k (2k + 1)^2 (k + 1)}{3(2n + 1)}\right) C_M \end{aligned}$$

for all *n*, *m*. □

#### Remark 4.37

The centreless BCCA is strongly almost-graded according to [78, Definition 4.1]: setting $\mathfrak {b}_n :={{\,\textrm{span}\,}}\{u_n, v_n\}$ for $n \in \mathbb {N}$ (where $u_0 = 0$), we see that

$$\begin{aligned} {[}\mathfrak {b}_n, \mathfrak {b}_m] \subseteq \bigoplus _{k = n + m - 2}^{n + m} \mathfrak {b}_k \end{aligned}$$

for all $n,m \in \mathbb {N}$, by Corollary 4.34. However, Corollary 4.36 shows that cocycle on $\mathfrak {b}$ giving rise to $\widehat{\mathfrak {b}}$ is not compatible with the almost-grading, in the sense that is impossible to extend the almost-grading of $\mathfrak {b}$ to an almost-grading of $\widehat{\mathfrak {b}}$.

This motivates our approach in later sections, where we focus on the centreless BCCA, since we would like to use the new basis $\{u_n, v_m \mid n \ge 1, m \ge 0\}$ to study the representation theory of the BCCA. This becomes very difficult if we have to deal with the central extension as described in Corollary 4.36. Indeed, when one has an almost-graded Lie algebra, the *local* central extensions are generally preferred and are better understood (see [77, Definition 4.1]). The central extension of $\mathfrak {b}$ giving rise to $\widehat{\mathfrak {b}}$ is not local with respect to the almost-grading defined above, making it difficult to work with.

Furthermore, recall that our goal is to construct a decreasing filtration on the BCCA. However, the observations above imply that this central extension of the BCCA is not compatible with the construction of a filtration for the BCCA as per Definition 2.10. This is because any filtration on $\widehat{\mathfrak {b}}$ would necessarily fail to be weakly convergent. Because of this, we will restrict our attention to the centreless BCCA in Sects. 5 and 6.

Finally, we consider what happens to $\mathcal {P}$ under the change of variables s↦t. Since *r* is a square root of $s^2 - 4$ by (4.4), we get that $r \mapsto \sqrt{t^2 - 4} = \sqrt{f}$ under this change of variables.

#### Corollary 4.38

Under the change of variables s↦t, the $\mathcal {O}$-module $\mathcal {P}$ corresponds to the $\mathfrak {f}$-module $\mathfrak {m} :=\Bbbk [t] \sqrt{f} \ dt^{-1}$. In other words, $\mathfrak {b}\cong \mathfrak {f}\ltimes \mathfrak {m}$.

#### Proof

Follows from Theorems 4.16 and 4.30, and the observation that $r = \sqrt{s^2 - 4}$ from (4.4). □

We finish by summarising the results of this section for the centreless BCCA.

- We have defined two different bases for the centreless BCCA, denoted by $\{\mathcal {O}_n, \mathcal {P}_m \mid n \ge 1, m \ge 0\}$ and $\{u_n, v_m \mid n \ge 1, m \ge 0\}$. The Lie bracket of $\mathfrak {b}$ with these two bases is given by
  $$\begin{aligned} {[}\mathcal {O}_n,\mathcal {O}_m]&= (n - m)\mathcal {O}_{n + m} - (n + m)\mathcal {O}_{n - m}  &   (n,m \ge 1), \\ {[}\mathcal {O}_n, P_m]&= (n - m)P_{n + m} + (n + m)P_{n - m}  &   (n \ge 1, m \ge 0), \\ {[}P_n,P_m]&= 0  &   (n,m \ge 0), \end{aligned}$$
  $$\begin{aligned} {[}u_n,u_m]&= (n - m)(u_{n + m} - 4u_{n + m - 2})  &   (n,m \ge 1), \\ {[}u_n,v_m]&= (n - m)v_{n + m} - 4(n - m - 1)v_{n + m - 2}  &   (n \ge 1, m \ge 0), \\ {[}v_n,v_m]&= 0  &   (n,m \ge 0). \end{aligned}$$
- In basis-free notation, we have $\mathcal {O}= \Bbbk [t + t^{-1}](t^2 - 1)\partial$ and $\mathcal {P}= \Bbbk [t + t^{-1}] t \ dt^{-1}$. Letting $s = t + t^{-1}$, $r = t - t^{-1}$, and $\partial _s = \frac{1}{s'}\partial = \frac{t^2}{t^2 - 1}\partial$, we can also write $\mathcal {O}$ and $\mathcal {P}$ as
  $$\begin{aligned} \mathcal {O}= \Bbbk [s](s^2 - 4)\partial _s, \quad \mathcal {P}= \Bbbk [s] r \ ds^{-1}. \end{aligned}$$
- There is an isomorphism $\Phi :\mathcal {O}\ltimes \mathcal {P}\rightarrow \mathfrak {f}\ltimes \mathfrak {m}$ given by the change of variables s↦t, where $\mathfrak {f}= f\mathbb {W}_1 = (t^2 - 4)\mathbb {W}_1$ and $\mathfrak {m} = \Bbbk [t]\sqrt{f} \ dt^{-1}$. Explicitly, $\Phi (u_n) = f_n$ and $\Phi (v_n) = -t^n\sqrt{f} \ dt^{-1}$ for all *n*, where $f_n = L_n - 4L_{n - 2}$. In basis-free notation,
  $$\begin{aligned} \Phi (p(s)(s^2 - 4)\partial _s) = p(t)(t^2 - 4)\partial , \quad \Phi (p(s)r \ ds^{-1}) = p(t)\sqrt{f} \ dt^{-1} \end{aligned}$$
  for all $p \in \Bbbk [t]$. In particular, $\mathcal {O}$ is isomorphic to a subalgebra of $\mathbb {W}_1$ of finite codimension.

## Filtering the BCCA

### Defining the filtration

Using the alternate basis for the BCCA from Corollary 4.34, we construct a descending filtration for $\mathfrak {b}$, thus allowing us to define and study Whittaker modules for the BCCA. We first filter the subalgebra $\mathcal {O}$ of the BCCA. It is convenient to introduce a shift in the basis $u_n$ of $\mathcal {O}$: define

$$\begin{aligned} \mathcal {U}_n :=u_{n + 2} = -(t + t^{-1})^{n + 1}(t^2 - 1)\partial = -s^{n + 1}(s^2 - 4)\partial _s \end{aligned}$$

for n≥-1. We can now construct a filtration of $\mathcal {O}$.

#### Lemma 5.1

For $$n \in \mathbb {Z}$$, define

$$\begin{aligned} \mathcal {F}_n\mathcal {O}:=s^{n + 1}\mathcal {O}= {{\,\textrm{span}\,}}\{\mathcal {U}_k \mid k \ge n\}. \end{aligned}$$

Then, $\mathcal {F}$ is a filtration of $\mathcal {O}$.

#### Proof

We have

5.1 $$\begin{aligned} \begin{aligned} {[}\mathcal {U}_n,\mathcal {U}_m]&= [u_{n + 2},u_{m + 2}] = (n - m)(u_{n + m + 4} - 4u_{n + m + 2}) \\&= (n - m)(\mathcal {U}_{n + m + 2} - 4 \, \mathcal {U}_{n + m}), \end{aligned} \end{aligned}$$

so we see that $[\mathcal {F}_n\mathcal {O}, \mathcal {F}_m\mathcal {O}] \subseteq \mathcal {F}_{n + m}\mathcal {O}$ for all *n*, *m*. □

We now extend the filtration $\mathcal {F}$ to the full BCCA. As before, we must shift the basis $v_n$: define

$$\begin{aligned} \mathcal {V}_n :=v_{n + 1} = -(t + t^{-1})^{n + 1} t \ dt^{-1} = -s^{n + 1} r \ ds^{-1} \end{aligned}$$

for n≥-1.

#### Proposition 5.2

For $$n \in \mathbb {Z}$$, define

$$\begin{aligned} \mathcal {F}_n\mathcal {P}:=s^{n + 1}\mathcal {P}= {{\,\textrm{span}\,}}\{\mathcal {V}_k \mid k \ge n\}. \end{aligned}$$

Then $[\mathcal {F}_n\mathcal {O},\mathcal {F}_m\mathcal {P}] \subseteq \mathcal {F}_{n + m}\mathcal {P}$ for all $n,m \in \mathbb {Z}$. Consequently, we can filter the full centreless BCCA as follows:

$$\begin{aligned} \mathcal {F}_n \mathfrak {b}= \mathcal {F}_n (\mathcal {O}\ltimes \mathcal {P}) :=\mathcal {F}_n \mathcal {O}\ltimes \mathcal {F}_n \mathcal {P}= {{\,\textrm{span}\,}}\{\mathcal {U}_k, \mathcal {V}_k \mid k \ge n\}. \end{aligned}$$

#### Proof

For all n,m≥-1, we have

5.2 $$\begin{aligned} \begin{aligned} {[}\mathcal {U}_n,\mathcal {V}_m]&= [u_{n + 2},v_{m + 1}] = (n - m + 1)v_{n + m + 3} - 4(n - m)v_{n + m + 1} \\&= (n - m + 1)\mathcal {V}_{n + m + 2} - 4(n - m)\mathcal {V}_{n + m}, \end{aligned} \end{aligned}$$

and thus $[\mathcal {F}_n\mathcal {O}, \mathcal {F}_m \mathcal {P}] \subseteq \mathcal {F}_{n + m} \mathcal {P}$. It is now clear that $\mathcal {F}$ is a filtration of the entirety of $\mathfrak {b}$, by Lemma 5.1. In other words, $[\mathcal {F}_n\mathfrak {b},\mathcal {F}_m\mathfrak {b}] \subseteq \mathcal {F}_{n + m}\mathfrak {b}$, and

$$\begin{aligned} \mathfrak {b}= \mathcal {F}_{-1}\mathfrak {b}\supseteq \mathcal {F}_{0}\mathfrak {b}\supseteq \mathcal {F}_{1}\mathfrak {b}\supseteq \dots . \end{aligned}$$

□

We finish this section by considering the associated graded algebra to the filtration $\mathcal {F}$.

#### Lemma 5.3

The associated graded Lie algebras to the filtration $\mathcal {F}$ are:

1. $${{\,\textrm{gr}\,}}_\mathcal {F}\mathcal {O}\cong \mathbb {W}_1$$, and
2. ${{\,\textrm{gr}\,}}_\mathcal {F}\mathfrak {b}\cong \mathbb {W}_1 \ltimes {{\,\textrm{ad}\,}}(\mathbb {W}_1)$, where ${{\,\textrm{ad}\,}}(\mathbb {W}_1)$ is another copy of $\mathbb {W}_1$, viewed as the adjoint module of $\mathbb {W}_1$ with abelian Lie bracket.

#### Proof

We have

$$\begin{aligned} {[}\mathcal {U}_n + \mathcal {F}_{n+1}\mathcal {O}, \mathcal {U}_m + \mathcal {F}_{m + 1}\mathcal {O}]&= [\mathcal {U}_n,\mathcal {U}_m]+\mathcal {F}_{m+n+1}\mathcal {O}\\&= -4(n - m)\mathcal {U}_{n + m} + \mathcal {F}_{n + m + 1}\mathcal {O}. \end{aligned}$$

Thus, the Lie algebra homomorphism $\mathcal {U}_n + \mathcal {F}_{n + 1}\mathcal {O}\mapsto -4L_n$ establishes the isomorphism ${{\,\textrm{gr}\,}}_\mathcal {F}\mathcal {O}\cong \mathbb {W}_1$. Similarly, we have

$$\begin{aligned} {[}\mathcal {U}_n + \mathcal {F}_{n + 1}\mathfrak {b}, \mathcal {V}_m + \mathcal {F}_{m + 1}\mathfrak {b}]&= [\mathcal {U}_n,\mathcal {V}_m]+\mathcal {F}_{n+m+1}\mathfrak {b}\\&= -4(n - m)\mathcal {V}_{n + m} + \mathcal {F}_{n + m + 1}\mathfrak {b}. \end{aligned}$$

It is now clear that ${{\,\textrm{gr}\,}}_\mathcal {F}\mathfrak {b}\cong \mathbb {W}_1 \ltimes {{\,\textrm{ad}\,}}(\mathbb {W}_1)$ using a similar isomorphism to the above, as required. □

### The derived subalgebras of the filtered subspaces

To study Whittaker functions on $\mathcal {O}$ and $\mathfrak {b}$, we must study their Whittaker functions. By definition, these are Lie algebra homomorphisms $\mathcal {F}_n \mathcal {O}\rightarrow \Bbbk$ or $\mathcal {F}_n \mathfrak {b}\rightarrow \Bbbk$, for some $n \in \mathbb {N}$. Since k is an abelian Lie algebra, these homomorphisms must necessarily vanish on the derived subalgebras $[\mathcal {F}_n \mathcal {O}, \mathcal {F}_n \mathcal {O}]$ or $[\mathcal {F}_n \mathfrak {b}, \mathcal {F}_n \mathfrak {b}]$. We compute these in this section.

First, note that $[\mathcal {F}_n\mathcal {O},\mathcal {F}_n\mathcal {O}] \subseteq \mathcal {F}_{2n + 1}\mathcal {O}$, by (5.1). We now prove that $[\mathcal {F}_n\mathcal {O},\mathcal {F}_n\mathcal {O}]$ is spanned by $\mathcal {U}_{2n + 2 + k} - 4 \, \mathcal {U}_{2n + k}$ for k≥1.

#### Lemma 5.4

For all n≥-1, the derived subalgebra $[\mathcal {F}_n\mathcal {O},\mathcal {F}_n\mathcal {O}]$ has the following form:

$$\begin{aligned} {[}\mathcal {F}_n\mathcal {O},\mathcal {F}_n\mathcal {O}] = (s^{2n + 4} - 4\,s^{2n + 2})\mathcal {O}= (s^{2n + 4} - 4\,s^{2n + 2})(s^2 - 4)\Bbbk [s]\partial _s. \end{aligned}$$

Consequently, the set $\{\mathcal {U}_{2n + 3 + k} - 4 \, \mathcal {U}_{2n + 1 + k} \mid k \in \mathbb {N}\}$ is a basis for $[\mathcal {F}_n\mathcal {O},\mathcal {F}_n\mathcal {O}]$.

#### Proof

Recall from Theorem 4.16 that $\mathcal {O}= (s^2 - 4)\Bbbk [s]\partial _s$, and that $\mathcal {F}_n\mathcal {O}= s^{n + 1}\mathcal {O}$ by definition. Therefore,

$$\begin{aligned} {[}\mathcal {F}_n\mathcal {O},\mathcal {F}_n\mathcal {O}] = [s^{n + 1}(s^2 - 4)\Bbbk [s]\partial _s, s^{n + 1}(s^2 - 4)\Bbbk [s]\partial _s] = \Big (s^{n + 1}(s^2 - 4)\Big )^2\Bbbk [s]\partial _s, \end{aligned}$$

by (4.1). Simplifying, we get

$$\begin{aligned} {[}\mathcal {F}_n\mathcal {O},\mathcal {F}_n\mathcal {O}]= &   \Big (s^{n + 1}(s^2 - 4)\Big )^2\Bbbk [s]\partial _s = s^{2n + 2}(s^2 - 4)^2\Bbbk [s]\partial _s \\= &   (s^{2n + 4} - 4s^{2n + 2})(s^2 - 4)\Bbbk [s]\partial _s. \end{aligned}$$

Noting that

$$\begin{aligned} \mathcal {U}_{2n + 3 + k} - 4 \, \mathcal {U}_{2n + 1 + k} = (s^{2n + 4 + k} - 4\,s^{2n + 2 + k})(s^2 - 4)\partial _s, \end{aligned}$$

it is now clear that the result follows. □

We now proceed as in Lemma 5.4 to compute the derived subalgebra $[\mathcal {F}_n\mathfrak {b},\mathcal {F}_n\mathfrak {b}]$ for the filtration of the full BCCA. Similarly to the above, (5.2) implies that $\mathcal {F}_n\mathcal {O}\cdot \mathcal {F}_n\mathcal {P}\subseteq \mathcal {F}_{2n + 1}\mathcal {P}$. In fact, this is an equality: we have $\mathcal {F}_n\mathcal {O}\cdot \mathcal {F}_n\mathcal {P}= \mathcal {F}_{2n + 1} \mathcal {P}$, as we prove next.

#### Lemma 5.5

For all n≥-1, we have $[\mathcal {F}_n\mathcal {O},\mathcal {F}_n\mathcal {P}] = \mathcal {F}_{2n + 1}\mathcal {P}$. Consequently,

$$\begin{aligned} {[}\mathcal {F}_n\mathfrak {b},\mathcal {F}_n\mathfrak {b}] = [\mathcal {F}_n\mathcal {O},\mathcal {F}_n\mathcal {O}] \ltimes \mathcal {F}_{2n + 1}\mathcal {P}, \end{aligned}$$

and therefore the set $\{\mathcal {U}_{2n + 3 + k} - 4 \, \mathcal {U}_{2n + 1 + k}, \mathcal {V}_{2n + 1 + k} \mid k \in \mathbb {N}\}$ is a basis for $[\mathcal {F}_n\mathfrak {b},\mathcal {F}_n\mathfrak {b}]$.

#### Proof

For all m≥n, we have

$$\begin{aligned} \mathcal {V}_{2m + 1}&= \frac{1}{4}[\mathcal {U}_m,\mathcal {V}_{m + 1}] \in [\mathcal {F}_n\mathcal {O},\mathcal {F}_n\mathcal {P}], \\ \mathcal {V}_{2m + 2}&= [\mathcal {U}_m,\mathcal {V}_m] \in [\mathcal {F}_n\mathcal {O},\mathcal {F}_n\mathcal {P}], \end{aligned}$$

by (5.2). The result follows. □

### The abelianisations of the filtered subspaces

We finish this section by computing the dimensions of the abelianisations of $\mathcal {F}_n \mathcal {O}$ and $\mathcal {F}_n \mathfrak {b}$. This computation will tell us exactly how many degrees of freedom one has when defining a Whittaker function on $\mathcal {O}$ or on $\mathfrak {b}$. The following easy observation will be useful for this purpose.

#### Lemma 5.6

For any $g \in \Bbbk [t]$, the codimension of $g\mathbb {W}_1$ in $\mathbb {W}_1$ is deg(g). □

Of course, Lemma 5.6 also implies that $g(s)\Bbbk [s]\partial _s$ has codimension deg(g) in $\Bbbk [s]\partial _s$, via the change of variables s↦t.

#### Proposition 5.7

We have

$$\begin{aligned} \dim \left( \frac{\mathcal {F}_n\mathcal {O}}{[\mathcal {F}_n\mathcal {O},\mathcal {F}_n\mathcal {O}]}\right) = n + 3, \quad \dim \left( \frac{\mathcal {F}_n\mathfrak {b}}{[\mathcal {F}_n\mathfrak {b},\mathcal {F}_n\mathfrak {b}]}\right) = 2n + 4, \end{aligned}$$

for all n≥-1.

#### Proof

By Theorem 4.16, we have

$$\begin{aligned} \mathcal {F}_n\mathcal {O}= s^{n + 1}(s^2 - 4)\Bbbk [s]\partial _s. \end{aligned}$$

This is a subalgebra of $\Bbbk [s]\partial _s$ of codimension n+3, by Lemma 5.6. On the other hand, Lemma 5.6 also implies that

$$\begin{aligned} {[}\mathcal {F}_n\mathcal {O},\mathcal {F}_n\mathcal {O}] = \Big (s^{n + 1}(s^2 - 4)\Big )^2\Bbbk [s]\partial _s \end{aligned}$$

is a subalgebra of $\Bbbk [s]\partial _s$ of codimension 2n+6. Therefore, $[\mathcal {F}_n\mathcal {O},\mathcal {F}_n\mathcal {O}]$ has codimension n+3 in $\mathcal {F}_n\mathcal {O}$, as required.

For the second part of the result, we observe that the codimension of $\mathcal {F}_m\mathcal {P}$ in $\mathcal {F}_n\mathcal {P}$ is m-n, for all $m \ge n \ge -1$. Now, Lemmas 5.5 and 5.6 imply that

$$\begin{aligned} \dim \left( \frac{\mathcal {F}_n\mathfrak {b}}{[\mathcal {F}_n\mathfrak {b},\mathcal {F}_n\mathfrak {b}]}\right) = \dim \left( \frac{\mathcal {F}_n\mathcal {O}}{[\mathcal {F}_n\mathcal {O},\mathcal {F}_n\mathcal {O}]}\right) + \dim (\mathcal {F}_n\mathcal {P}/\mathcal {F}_{2n + 1}\mathcal {P}) = n + 3 + n + 1 = 2n + 4, \end{aligned}$$

which concludes the proof. □

More precisely, write $\overline{x}$ for the image of *x* in the abelianisation $\mathcal {F}_n \mathfrak {b}/[\mathcal {F}_n \mathfrak {b}, \mathcal {F}_n \mathfrak {b}]$ or $\mathcal {F}_n \mathcal {O}/[\mathcal {F}_n \mathcal {O}, \mathcal {F}_n \mathcal {O}]$, where $x \in \mathcal {F}_n\mathfrak {b}$ or $x \in \mathcal {F}_n \mathcal {O}$, and $n \in \mathbb {Z}$. Then Proposition 5.7 implies that $\{\overline{\mathcal {U}}_n, \dots , \overline{\mathcal {U}}_{2n+2}\}$ forms a basis of $\mathcal {F}_n \mathcal {O}/[\mathcal {F}_n\mathcal {O},\mathcal {F}_n\mathcal {O}]$, and $\{\overline{\mathcal {U}}_n, \dots , \overline{\mathcal {U}}_{2n+2}\} \cup \{\overline{\mathcal {V}}_n, \dots , \overline{\mathcal {V}}_{2n}\}$ forms a basis of $\mathcal {F}_n \mathfrak {b}/[\mathcal {F}_n\mathfrak {b},\mathcal {F}_n\mathfrak {b}]$.

The simplest application of Proposition 5.7 is that we can completely classify all one-dimensional representations of $\mathcal {O}$ and $\mathfrak {b}$.

#### Definition 5.8

Let $\lambda , \mu \in \Bbbk$, and define the $\mathcal {O}$-module $\Bbbk _{\lambda ,\mu }$ as follows: as a vector space, $\Bbbk _{\lambda ,\mu } = \Bbbk 1_{\lambda ,\mu }$, and the $\mathcal {O}$-action is given by

$$\begin{aligned} \mathcal {U}_{-1} \cdot 1_{\lambda ,\mu } = \lambda 1_{\lambda ,\mu }, \quad \mathcal {U}_0 \cdot 1_{\lambda ,\mu } = \mu 1_{\lambda ,\mu }. \end{aligned}$$

The action of the other basis elements is determined by these and the formula

$$\begin{aligned} \mathcal {U}_n \cdot 1_{\lambda ,\mu } = 4 \, \mathcal {U}_{n - 2} \cdot 1_{\lambda ,\mu } \quad (n \ge 1). \end{aligned}$$

For example, we have $\mathcal {U}_1 \cdot 1_{\lambda ,\mu } = 4\lambda 1_{\lambda ,\mu }$ and $\mathcal {U}_2 \cdot 1_{\lambda ,\mu } = 4\mu 1_{\lambda ,\mu }$. In fact, all one-dimensional representations of $\mathcal {O}$ and $\mathfrak {b}$ are of the above form, which is an immediate consequence of Lemmas 5.4 and 5.5, and Proposition 5.7.

#### Corollary 5.9

If *M* is a one-dimensional $\mathcal {O}$-representation, then $M \cong \Bbbk _{\lambda ,\mu }$ for some $\lambda , \mu \in \Bbbk$. If *M* is a one-dimensional $\mathfrak {b}$-representation, then $\mathcal {P}\cdot M = 0$, so *M* can be viewed as a representation over $\mathfrak {b}/\mathcal {P}\cong \mathcal {O}$. □

## Whittaker modules

Having defined and analysed the filtrations for $\mathcal {O}$ and $\mathfrak {b}$, we now study Whittaker modules for these Lie algebras (recall Lemma 2.11). We emphasise that the construction of the filtration from Sect. 5 is not compatible with the known central extension of the BCCA, so we focus on the centreless BCCA in this section.

### Whittaker modules for $\mathcal {O}$

We start by defining Whittaker functions for $\mathcal {O}$, which in turn define universal Whittaker modules for $\mathcal {O}$. We further characterise precisely when these modules are irreducible.

#### Definition 6.1

A *Whittaker function on* $\mathcal {O}$ is a Lie algebra homomorphism $\varphi _n :\mathcal {F}_n\mathcal {O}\rightarrow \Bbbk$ for some $n \in \mathbb {N}$.

As we show next, a Whittaker function $\varphi _n :\mathcal {F}_n \mathcal {O}\rightarrow \Bbbk$ is determined by its values on $\mathcal {U}_n, \dots , \mathcal {U}_{2n+2}$.

#### Lemma 6.2

A Whittaker function $\varphi _n :\mathcal {F}_n\mathcal {O}\rightarrow \Bbbk$ is determined by its values on $\mathcal {U}_n, \dots , \mathcal {U}_{2n+2}$, and $\varphi _n(\mathcal {U}_{m + 2}) = 4\varphi _n(\mathcal {U}_m)$ for all m≥2n+1.

#### Proof

Follows immediately from Lemma 5.4 and Proposition 5.7. □

#### Definition 6.3

Let $\varphi _n :\mathcal {F}_n\mathcal {O}\rightarrow \Bbbk$ be a Whittaker function and $\Bbbk _{\varphi _n} = \Bbbk 1_{\varphi _n}$ be the one-dimensional $\mathcal {F}_n\mathcal {O}$-module defined by $\varphi _n$, in other words,

$$\begin{aligned} x \cdot 1_{\varphi _n} = \varphi _n(x)1_{\varphi _n} \end{aligned}$$

for all $x \in \mathcal {F}_n\mathfrak {b}$. Define the *universal Whittaker module for* $\mathcal {O}$ *of type* $\varphi _n$ to be the induced module

$$\begin{aligned} M_{\varphi _n} :={{\,\textrm{Ind}\,}}_{\mathcal {F}_n\mathcal {O}}^{\mathcal {O}}\Bbbk _{\varphi _n}. \end{aligned}$$

#### Remark 6.4

Note that $\Bbbk _{\varphi _n}$ is a continuous module for $\mathcal {F}_n\mathcal {O}$ in the sense of Rudakov [75] if and only if $\varphi _n(\mathcal {U}_{2n + 1}) = \varphi _n(\mathcal {U}_{2n + 2}) = 0$. In fact, if $\varphi _n(\mathcal {U}_{2n + 1}) \ne 0$, then $\varphi (\mathcal {U}_{2n + 2k + 1}) \ne 0$ for all k≥0, and if $\varphi _n(\mathcal {U}_{2n + 2}) \ne 0$, then $\varphi _n(\mathcal {U}_{2m + 2k + 2}) \ne 0$ for all k≥0, by Lemma 6.2.

#### Remark 6.5

The Lie algebra $\mathcal {F}_0 \mathcal {O}$ is not quasi-nilpotent [30, Remark 3.5], so $M_{\varphi _0}$ is not a Whittaker module according to Definition 2.9. As we will see, the criteria for irreducibility for $M_{\varphi _0}$ and their proofs are slightly different to those for $M_{\varphi _n}$ with n≥1, highlighting the importance of the quasi-nilpotence condition. However, to avoid piling onto the nomenclature, we abuse terminology and opt to call $M_{\varphi _0}$ a universal Whittaker module.

We also highlight that $\mathcal {F}_n \mathcal {O}$ is not an ideal of $\mathcal {O}$ if n≥0. Therefore, the universal Whittaker modules $M_{\varphi _n}$ are never quasi-Whittaker modules according to Definition 2.12.

The following result is the main theorem of this subsection.

#### Theorem 6.6

Let $n \in \mathbb {N}$, and let $\varphi _n :\mathcal {F}_n \mathcal {O}\rightarrow \Bbbk$ be a Whittaker function. If n=0, the universal Whittaker module $M_{\varphi _0}$ is irreducible if and only if $\varphi _0(\mathcal {U}_{2}) \ne 4 \varphi _0(\mathcal {U}_{0})$. If n≥1, the universal Whittaker module $M_{\varphi _n}$ is irreducible if and only if $\varphi _n(\mathcal {U}_{2n + 1}) \ne 4 \varphi _n(\mathcal {U}_{2n - 1})$ or $\varphi _n(\mathcal {U}_{2n + 2}) \ne 4\varphi _n(\mathcal {U}_{2n})$.

We now proceed to prove that $M_{\varphi _n}$ is reducible if the condition from Theorem 6.6 is not satisfied, proving one direction of Theorem 6.6. As mentioned in Remark 6.5, the proofs are slightly different depending on whether n=0 or n≥1.

#### Lemma 6.7

Let $\varphi _0 :\mathcal {F}_0 \mathcal {O}\rightarrow \Bbbk$ be a Whittaker function with $\varphi _0(\mathcal {U}_{2}) = 4\varphi _0(\mathcal {U}_{0})$. Then $U(\mathcal {O})\mathcal {U}_{-1} \cdot 1_{\varphi _0}$ is a proper submodule of the universal Whittaker module $M_{\varphi _0}$.

#### Proof

Let $\lambda _i :=\varphi _n(\mathcal {U}_{i})$ for i≥0, and $w :=(\lambda _1 - 4 \, \mathcal {U}_{-1}) \cdot 1_{\varphi _0}=[\mathcal {U}_0, \mathcal {U}_{-1}]\cdot 1_{\varphi _0}$. We claim that $U(\mathcal {O}) \cdot w$ is a proper $\mathcal {O}$-submodule of $M_{\varphi _0}$.

Lemma 6.2 together with the assumption that $\lambda _2 = 4\lambda _0$ imply that $\lambda _{n + 2} = 4\lambda _n$ for all n≥0. Then,

$$\begin{aligned} \mathcal {U}_0 \cdot w&= \mathcal {U}_0(\lambda _1 - 4 \, \mathcal {U}_{-1}) \cdot 1_{\varphi _0} \\&= \Big (\lambda _0\lambda _1 - 4[\mathcal {U}_0,\mathcal {U}_{-1}] - 4 \, \mathcal {U}_{-1}\mathcal {U}_0\Big ) \cdot 1_{\varphi _0} \\&= \Big (\lambda _0\lambda _1 - 4(\mathcal {U}_1 - 4 \, \mathcal {U}_{-1}) - 4 \lambda _0 \, \mathcal {U}_{-1}\Big ) \cdot 1_{\varphi _0} \\&= \Big ((\lambda _0 - 4)\lambda _1 - 4(\lambda _0 - 4)\mathcal {U}_{-1}\Big ) \cdot 1_{\varphi _0} \\&= (\lambda _0 - 4)w. \end{aligned}$$

Now letting n≥1, we have

$$\begin{aligned} \mathcal {U}_n \cdot w&= \mathcal {U}_n(\lambda _1 - 4\,\mathcal {U}_{-1}) \cdot 1_{\varphi _0} \\&= \Big (\lambda _n\lambda _1 - 4[\mathcal {U}_n,\mathcal {U}_{-1}] - 4\,\mathcal {U}_{-1}\mathcal {U}_n\Big ) \cdot 1_{\varphi _0} \\&= \Big (\lambda _n\lambda _1 - 4(n + 1)(\mathcal {U}_{n + 1} - 4\,\mathcal {U}_{n - 1}) - 4\lambda _n \, \mathcal {U}_{-1}\Big ) \cdot 1_{\varphi _0} \\&= \lambda _n (\lambda _1 - 4\,\mathcal {U}_{-1}) \cdot 1_{\varphi _0} - 4(n + 1)(\lambda _{n + 1} - 4\lambda _{n - 1}) \cdot 1_{\varphi _0} \\&= \lambda _n w, \end{aligned}$$

where we used that $\lambda _{n + 1} = 4\lambda _{n - 1}$ in the last equality, since n≥1. This proves the claim, and the result follows. □

#### Remark 6.8

When $\lambda _2 = 4\lambda _0$, the submodule $U(\mathcal {O})(\lambda _1 - 4 \, \mathcal {U}_{-1}) \cdot 1_{\varphi _0}$ in the proof of Lemma 6.7 is isomorphic to $M_{\varphi _0'}$, where $\varphi '_0 :\mathcal {F}_0\mathcal {O}\rightarrow \Bbbk$ is the Whittaker function with $\varphi '_0(\mathcal {U}_0) = \lambda _0 - 4$ and $\varphi '_0(\mathcal {U}_n) = \lambda _n$ for n≥1.

In this case, the quotient module $M_{\varphi _0}/(U(\mathcal {O})(\lambda _1 - 4 \, \mathcal {U}_{-1}) \cdot 1_{\varphi _0})$ is a 1-dimensional $\mathcal {O}$-module determined by $\mathcal {U}_{-1} \cdot 1_{\varphi _0} = \frac{\lambda _1}{4} 1_{\varphi _0}, \mathcal {U}_{0} \cdot 1_{\varphi _0} = \lambda _0 1_{\varphi _0}$.

The case n≥1 follows from the existence of an analogous submodule.

#### Lemma 6.9

Let n≥1 and let $\varphi _n :\mathcal {F}_n \mathcal {O}\rightarrow \Bbbk$ be a Whittaker function with $\varphi _n(\mathcal {U}_{2n + 1}) = 4 \varphi _n(\mathcal {U}_{2n - 1})$ and $\varphi _n(\mathcal {U}_{2n + 2}) = 4\varphi _n(\mathcal {U}_{2n})$. Then, $U(\mathcal {O}) \mathcal {U}_{n - 1} \cdot 1_{\varphi _n}$ is a submodule of the universal Whittaker module $M_{\varphi _n}$.

#### Proof

Let $\lambda _i :=\varphi _n(\mathcal {U}_{i})$ for i≥n, and $w :=\mathcal {U}_{n - 1} \cdot 1_{\varphi _n}$. We claim that $U(\mathcal {O}) \cdot w$ is a proper submodule of $M_{\varphi _n}$.

Lemma 6.2 together with the assumptions that $\lambda _{2n + 1} = 4\lambda _{2n - 1}$ and $\lambda _{2n + 2} = 4\lambda _{2n}$ imply that $\lambda _{k + 2} = 4\lambda _k$ for all k≥2n-1. For m≥n, we have

$$\begin{aligned} \mathcal {U}_m \cdot w&= \mathcal {U}_m \mathcal {U}_{n - 1} \cdot 1_{\psi _n} = [\mathcal {U}_m,\mathcal {U}_{n - 1}] \cdot 1_{\psi _n} + \mathcal {U}_{n - 1} \mathcal {U}_m \cdot 1_{\psi _n} \\&= (m - n + 1) (\mathcal {U}_{m + n + 1} - 4 \, \mathcal {U}_{m + n - 1}) \cdot 1_{\psi _n} + \lambda _m w \\&= \lambda _m w, \end{aligned}$$

since m+n-1≥2n-1, where we used that $\lambda _{k + 2} = 4\lambda _k$ for all k≥2n-1.

The above shows that kw is a one-dimensional $\mathcal {F}_n \mathcal {O}$-module isomorphic to $\Bbbk _{\varphi _n}$. It is now easy to see that $U(\mathcal {O}) \cdot w$ is a proper submodule of $M_{\varphi _n}$ isomorphic to $M_{\varphi _n}$ itself. □

Note that together Lemmas 6.7 and 6.9 prove one direction of Theorem 6.6. To prove the converse, we use the theory of local functions and the orbit method, which we introduce in the next subsection.

### Local functions and the orbit method for $\mathcal {O}$

In this subsection, we make a key observation that the Whittaker function $\varphi _n$ from Theorem 6.6 can be viewed as the restriction of a certain element χ of $\mathbb {W}_1^*$ whose stabilizer has finite codimension in $\mathbb {W}_1$. Then Whittaker modules are exactly restrictions of modules from the Kirillov orbit method for $\mathbb {W}_1$ (see [71, 81]). Kirillov’s orbit method [58] is a guiding principle in Lie theory, establishing a correspondence between equivalence classes of unitary irreducible representations and coadjoint orbits. As a result, we will readily obtain the irreducibility criteria in Theorem 6.6.

Note that in this subsection, we view $\mathbb {W}_1 = {{\,\textrm{Der}\,}}(\Bbbk [s]) = \Bbbk [s]\partial _s$ (in other words, we use the variable *s* instead of *t*), so that we can view $\mathcal {O}$ as the subalgebra $(s^2 - 4)\mathbb {W}_1$ of $\mathbb {W}_1$. We start by recalling the notion of a local function on $\mathbb {W}_{1}$, originally defined in [72].

#### Definition 6.10

Letting $x, \alpha _0, \alpha _1, \dots ,\alpha _n \in \Bbbk$ with $\alpha _n \ne 0$, we define a *one-point local function* $\chi _{x; \alpha _0, \dots ,\alpha _n} \in \mathbb {W}_1^*$ by

6.1 $$\begin{aligned} \chi _{x; \alpha _0, \dots ,\alpha _n} :\mathbb {W}_{1} \rightarrow \Bbbk , \quad f \partial \mapsto \alpha _0 f(x) + \alpha _1 f'(x) + \dots +\alpha _n f^{(n)}(x), \end{aligned}$$

where $f \in \Bbbk [s]$ and $f^{(i)}$ denotes taking the $i^{\text {th}}$ derivative of *f* with respect to *s*. We call *x* the *base point* of χ and *n* the *order* of χ.

A *local function* χ on $\mathbb {W}_1$ is a sum of finitely many functions of the form  (6.1) with (possibly) distinct *x*. In other words,

$$\begin{aligned} \chi = \chi _1 + \dots + \chi _{\ell }, \end{aligned}$$

where $\chi _i$ is a one-point local function based at $x_i \in \Bbbk$ of order $n_i$, and the $x_i$ are pairwise distinct.

Next, we recall the notion of a polarisation for a local function on $\mathbb {W}_{1}$ in [71].

#### Definition 6.11

For every $\chi \in \mathbb {W}_{1}^*$, we define a bilinear form

$$\begin{aligned} B_\chi : \mathbb {W}_{1} \times \mathbb {W}_{1}&\rightarrow \Bbbk \\ (u, v)&\mapsto \chi ([u, v]). \end{aligned}$$

A *polarisation* $\mathfrak {p}_\chi$ of χ is a finite-codimensional subalgebra of $\mathbb {W}_{1}$ that is maximally totally isotropic subspace with respect to $B_\chi$, in other words, $B_\chi ([\mathfrak {p}_\chi , \mathfrak {p}_\chi ]) = 0$.

#### Remark 6.12

In general, an arbitrary element $\chi \in \mathfrak {g}^*$ for a Lie algebra $\mathfrak {g}$ might not have a polarisation. However, if χ is a local function on $\mathbb {W}_1$, then χ always has a polarisation [71, Proposition 3.2]. In this case, the polarisation can moreover be chosen as a submodule-subalgebra of $\mathbb {W}_1$.

We consider an example of a local function and its polarisation to illustrate the concept.

#### Example 6.13

([72]) Let $\chi :=\chi _{x; \nu _0, \nu _1}$ be a one-point local function on $\mathbb {W}_1$ where $x \in \Bbbk$ and $(\nu _0, \nu _1) \in \Bbbk ^2 \setminus \{(0, 0)\}$. In other words,

$$\begin{aligned} \chi (f\partial ) = \nu _0 f(x) + \nu _1 f'(x). \end{aligned}$$

Consider the submodule-subalgebra $(s - x)\mathbb {W}_1$ of $\mathbb {W}_1$. Certainly, $(s - x)\mathbb {W}_1$ has codimension 1 in $\mathbb {W}_1$. Moreover, (4.1) implies that

$$\begin{aligned} \chi ([(s - x)\mathbb {W}_1, (s - x)\mathbb {W}_1]) = \chi ((s - x)^2\mathbb {W}_1) = 0, \end{aligned}$$

and therefore $(s - x)\mathbb {W}_1$ is a totally isotropic subspace of $\mathbb {W}_1$ with respect to $B_\chi$. Note that $(s - x)\mathbb {W}_1$ is maximally totally isotropic by a dimension count [71, Lemma 3.1], and therefore is a polarisation of χ.

#### Remark 6.14

The polarisation $(s - x)\mathbb {W}_1$ is in fact the unique polarisation for the local function $\chi _{x; \nu _0, \nu _1}$ (see, for example, [72, Section 7.1] for a proof). However, polarisations for local functions of $\mathbb {W}_{1}$ are in general not unique (for an example, see [71, Example 3.3]).

#### Remark 6.15

If $\mathfrak {q}$ is a subalgebra of a polarisation of a local function χ, then $\chi ([\mathfrak {q},\mathfrak {q}]) = 0$, and thus, χ defines a one-dimensional representation of $\mathfrak {q}$:

$$\begin{aligned} { \left. \hspace{0.0pt}\chi \phantom {\big |} \right| _{\mathfrak {q}} } :\mathfrak {q}&\rightarrow \Bbbk \\ f\partial&\mapsto \chi (f\partial ). \end{aligned}$$

We now explain the connection between the Whittaker functions on $\mathcal {O}$ and the orbit method for the Lie algebra $\mathbb {W}_{1}$ constructed in [71, 81], which will allow us to use results in [71, 81] to prove the other direction of Theorem 6.6. The next result shows how to associate a local function to each Whittaker function $\varphi _n :\mathcal {F}_n \mathcal {O}\rightarrow \Bbbk$.

#### Proposition 6.16

Let $n \in \mathbb {N}$ and let $\varphi _n :\mathcal {F}_n \mathcal {O}\rightarrow \Bbbk$ be a Whittaker function. Then, there exists a local function $\chi = \chi _{0; \alpha _0, \dots , \alpha _{2n+1}} + \chi _{2; \beta , \beta _1} + \chi _{-2; \gamma , \gamma _1}$ on $\mathbb {W}_1$ such that $\varphi _n = { \left. \hspace{0.0pt}\chi \phantom {\big |} \right| _{\mathcal {F}_n \mathcal {O}} }$.

Moreover, we can always choose $\alpha _0, \beta , \gamma \in \Bbbk$ arbitrarily, and

$$\begin{aligned} \varphi _n(\mathcal {U}_{2n + 2}) \ne 4 \varphi _n(\mathcal {U}_{2n})&\iff \alpha _{2n+1}\ne 0, \\ \varphi _n(\mathcal {U}_{2n + 1}) \ne 4 \varphi _n(\mathcal {U}_{2n - 1})&\iff \alpha _{2n} \ne 0 \quad ({\text {provided }} n \ge 1). \end{aligned}$$

#### Proof

Choose arbitrary $\alpha _i, \beta , \beta _1, \gamma , \gamma _1 \in \Bbbk$, and let

6.2 $$\begin{aligned} \chi :=\chi _{0; \alpha _0, \dots , \alpha _{2n+1}} + \chi _{2; \beta , \beta _1} + \chi _{-2; \gamma , \gamma _1}. \end{aligned}$$

It is easy to check that χ vanishes on $s^{2n+2} (s^2-4)^2\mathbb {W}_1 = [\mathcal {F}_n \mathcal {O}, \mathcal {F}_n \mathcal {O}]$, thus $\mathcal {F}_n \mathcal {O}$ is contained in a polarisation of χ. Hence, χ restricts to a one-dimensional representation ${ \left. \hspace{0.0pt}\chi \phantom {\big |} \right| _{\mathcal {F}_n \mathcal {O}} }$ of $\mathcal {F}_n \mathcal {O}$. Since both ${ \left. \hspace{0.0pt}\chi \phantom {\big |} \right| _{\mathcal {F}_n \mathcal {O}} }$ and $\varphi _n$ vanish on $[\mathcal {F}_n \mathcal {O}, \mathcal {F}_n \mathcal {O}]$, we require the following system of equations to have ${ \left. \hspace{0.0pt}\chi \phantom {\big |} \right| _{\mathcal {F}_n \mathcal {O}} } = \varphi _n$:

6.3 $$\begin{aligned} \varphi _n(\mathcal {U}_k)&= (k + 3)! \alpha _{k + 3} - 4(k + 1)! \alpha _{k + 1} + 2^{k + 3}(\beta _1 + (-1)^k \gamma _1) {\text { for }} n \le k \le 2n-2,\end{aligned}$$

6.4 $$\begin{aligned} \varphi _n(\mathcal {U}_{2n-1})&= - 4(2n)! \alpha _{2n} + 2^{2n + 2}(\beta _1 + (-1)^{2n-1} \gamma _1) ,\end{aligned}$$

6.5 $$\begin{aligned} \varphi _n(\mathcal {U}_{2n})&= - 4(2n+1)! \alpha _{2n+1} + 2^{2n + 3}(\beta _1 + (-1)^{2n} \gamma _1), \end{aligned}$$

6.6 $$\begin{aligned} \varphi _n(\mathcal {U}_{2n+1})&= 2^{2n + 4}(\beta _1 + (-1)^{2n+1} \gamma _1), \end{aligned}$$

6.7 $$\begin{aligned} \varphi _n(\mathcal {U}_{2n+2})&= 2^{2n + 5}(\beta _1 + (-1)^{2n+2} \gamma _1). \end{aligned}$$

Note that (6.3) only exists for n≥2, and (6.4) only exists for n≥1. For all *n*, there are n+3 equations (from $\varphi _n(\mathcal {U}_{n})$ to $\varphi _n(\mathcal {U}_{2n+2})$) with n+3 variables (from $\alpha _{n+1}$ to $\alpha _{2n+1}$, as well as $\beta _1$, $\gamma _1$). Furthermore, there are no inconsistencies on the right-hand side; thus, there always exist $\alpha _{n+1}, \dots , \alpha _{2n+1}, \beta _1, \gamma _1 \in \Bbbk$ satisfying the above system. In other words, there always exists χ of the form (6.2) such that $\varphi _n = { \left. \hspace{0.0pt}\chi \phantom {\big |} \right| _{\mathcal {F}_n\mathcal {O}} }$. Since $\alpha _0, \beta , \gamma$ are not involved in the system of equations, we can choose them arbitrarily.

The last part of the statement follows from the combination of (6.4) and (6.6) (if n≥1), and the combination of (6.5) and (6.7). □

For the rest of this section, we fix $n \in \mathbb {N}$ and a Whittaker function $\varphi _n :\mathcal {F}_n \mathcal {O}\rightarrow \Bbbk$. Given Proposition 6.16, we introduce notation for the local function associated to $\varphi _n$.

#### Notation 6.17

For k≥n, define $\lambda _k :=\varphi _n(\mathcal {U}_k)$. Furthermore, using Proposition 6.16, choose $\alpha _i, \beta _1, \gamma _1 \in \Bbbk$ such that $\varphi _n = { \left. \hspace{0.0pt}\chi \phantom {\big |} \right| _{\mathcal {F}_n \mathcal {O}} }$, where

$$\begin{aligned} \chi :=\chi _{0; 1, \alpha _1, \dots , \alpha _{2n+1}} + \chi _{2; 1, \beta _1} + \chi _{-2; 1, \gamma _1}. \end{aligned}$$

We will denote

$$\begin{aligned} \eta :=\chi _{0; 1, \alpha _1, \dots , \alpha _{2n+1}}, \quad \theta _{a,b} :=\chi _{2; 1, a} + \chi _{-2; 1, b}, \quad \chi _{a,b} :=\eta + \theta _{a,b} \end{aligned}$$

for $a,b \in \Bbbk$. For simplicity of notation, write $\theta :=\theta _{\beta _1,\gamma _1}$, so that $\chi = \eta + \theta$.

Although it is not obvious at this point why we have introduced the family of local functions $\chi _{a,b}$, this soon become clear.

#### Remark 6.18

The splitting $\chi = \eta + \theta$ was introduced because θ defines a Lie algebra homomorphism $\theta :\mathcal {O}\rightarrow \Bbbk$, since θ vanishes on $[\mathcal {O},\mathcal {O}] = (s^2 - 4)^2\mathbb {W}_1$. Therefore, $\Bbbk _\theta$ is a one-dimensional representation of $\mathcal {O}$. This is not true for η, and we will soon see that η completely controls the reducibility of $M_{\varphi _n}$.

Having introduced the necessary background and notation, we proceed to prove the irreducibility of these representations under the conditions of Theorem 6.6, using the perspective of local functions. We begin this proof with realising the subalgebra $\mathcal {F}_n \mathcal {O}$ as a polarisation of the local function χ, using results in [81] and [71].

#### Lemma 6.19

([81, Theorem 3.5], [71, Proposition 3.2]) Let $\chi = \chi _1 + \dots + \chi _\ell$ be a local function on $\mathbb {W}_{1}$, where $\chi _i$ is a one-point local function at $x_i$ with order $n_i$. Letting $m_i :=\left\lfloor \frac{n_i}{2} \right\rfloor$, the subalgebra

$$\begin{aligned} \mathfrak {p}_{\chi _i} :=(s - x_i)^{m_i + 1}\mathbb {W}_1 \end{aligned}$$

is a polarisation of $\chi _i$. Furthermore,

$$\begin{aligned} \mathfrak {p}_\chi :=\mathfrak {p}_{\chi _1} \cap \dots \cap \mathfrak {p}_{\chi _\ell } = (s - x_1)^{m_1 + 1} \dots (s - x_\ell )^{m_\ell + 1}\mathbb {W}_1 \end{aligned}$$

is a polarisation of χ.

#### Corollary 6.20

If n=0, then $\mathcal {F}_0 \mathcal {O}$ is a polarisation of $\chi _{a,b}$ for all $a,b \in \Bbbk$. If n≥1, and $\lambda _{2n + 1} \ne 4 \lambda _{2n - 1}$ or $\lambda _{2n + 2} \ne 4\lambda _{2n}$, then $\mathcal {F}_n \mathcal {O}$ is a polarisation of $\chi _{a,b}$ for all $a,b \in \Bbbk$.

#### Proof

Throughout this proof, we assume that $\lambda _{2n + 1} \ne 4 \lambda _{2n - 1}$ or $\lambda _{2n + 2} \ne 4\lambda _{2n}$ if n≥1.

We first claim that $s^{n + 1}\mathbb {W}_1$ is a polarisation of η. If n=0, then by definition we have $\eta = \chi _{0;1,\alpha _1}$ for some $\alpha _1 \in \Bbbk$. Therefore, $\mathfrak {p}_\eta = s\mathbb {W}_1$ is a polarisation of η, by Lemma 6.19. If n≥1, then Proposition 6.16 implies that either $\alpha _{2n} \ne 0$ or $\alpha _{2n + 1} \ne 0$. Thus, by Lemma 6.19, it follows that $\mathfrak {p}_{\eta } = s^{n+1} \mathbb {W}_1$ is a polarisation of η. This proves the claim.

Recalling that $\theta _{a,b} = \chi _{2; 1, a} + \chi _{-2; 1, b}$ by definition, we also see that $\mathfrak {p}_{\theta _{a,b}} = (s^2 - 4)\mathbb {W}_1$ is a polarisation of $\theta _{a,b}$, by Lemma 6.19. We conclude that

$$\begin{aligned} \mathfrak {p}_{\chi _{a,b}} = \mathfrak {p}_{\eta + \theta _{a,b}} = s^{n+1} \mathbb {W}_1 \cap (s^2 - 4)\mathbb {W}_1 = s^{n + 1} (s^2 - 4)\mathbb {W}_1 = \mathcal {F}_n \mathcal {O}\end{aligned}$$

is a polarisation of $\chi _{a,b}$. □

The conditions under which ${{\,\textrm{Ind}\,}}^{\mathbb {W}_{1}}_{\mathfrak {p}_\chi } \Bbbk _\chi$ is a simple $\mathbb {W}_{1}$-module are completely characterised in [81] and [71]. We summarise the relevant results in the following theorem.

#### Theorem 6.21

([81, Theorem 4.17], [71, Corollary 4.27, Proposition 4.23]) Let $\chi = \chi _1 + \dots + \chi _\ell$ be a local function on $\mathbb {W}_{1}$, where $\chi _i$ is a one-point local function at $x_i$ with order $n_i$. Letting $m_i :=\left\lfloor \frac{n_i}{2} \right\rfloor$, let

$$\begin{aligned} \mathfrak {p}_\chi = (s - x_1)^{m_1 + 1} \dots (s - x_\ell )^{m_\ell + 1}\mathbb {W}_1 \end{aligned}$$

be the polarisation of χ from Lemma 6.19. Then, ${{\,\textrm{Ind}\,}}_{\mathfrak {p}_\chi }^{\mathbb {W}_{1}} \Bbbk _\chi$ is an irreducible $\mathbb {W}_{1}$-module if and only if $n_i > 0$ for all *i*.

Corollary 6.20 and Theorem 6.21 imply that ${{\,\textrm{Ind}\,}}_{\mathcal {F}_n \mathcal {O}}^{\mathbb {W}_1} \Bbbk _\chi$ can only be an irreducible $\mathbb {W}_1$-module if $\beta _1$ and $\gamma _1$ are nonzero. However, this is not guaranteed by Proposition 6.16. Thankfully, we can solve this issue: the reducibility of $M_{\varphi _n} = {{\,\textrm{Ind}\,}}_{\mathcal {F}_n \mathcal {O}}^{\mathcal {O}} \Bbbk _{\chi }$ does not depend on the values of $\beta _1$ and $\gamma _1$, as we aim to show next. In other words, the reducibility of $M_{\varphi _n}$ is equivalent to that of ${{\,\textrm{Ind}\,}}_{\mathcal {F}_n \mathcal {O}}^{\mathcal {O}} \Bbbk _{\chi _{a,b}}$ for any choice of $a,b \in \Bbbk$.

We now decompose the universal Whittaker module of $\mathcal {O}$ into the tensor product of an induced module of a local function and a one-dimensional representation. This helps us view the universal Whittaker module of $\mathcal {O}$ as a twisted induced module of the one-point local function η.

#### Lemma 6.22

Let $a,b \in \Bbbk$, and let $\Bbbk _{\eta }$ and $\Bbbk _{\theta _{a,b}}$ be the one-dimensional representations of $\mathcal {F}_n \mathcal {O}$ and $\mathcal {O}$ induced by η and $\theta _{a,b}$, respectively. Then,

$$\begin{aligned} {{\,\textrm{Ind}\,}}_{\mathcal {F}_n \mathcal {O}}^\mathcal {O}\Bbbk _{\chi _{a,b}} \cong ({{\,\textrm{Ind}\,}}_{\mathcal {F}_n \mathcal {O}}^\mathcal {O}\Bbbk _\eta ) \otimes \Bbbk _{\theta _{a,b}}. \end{aligned}$$

In particular,

$$\begin{aligned} M_{\varphi _n} \cong ({{\,\textrm{Ind}\,}}^{\mathcal {O}}_{\mathcal {F}_n \mathcal {O}} \Bbbk _{\eta }) \otimes \Bbbk _{\theta }. \end{aligned}$$

#### Proof

Note that $\Bbbk _{\chi _{a,b}} = \Bbbk _{\eta + \theta _{a,b}} \cong \Bbbk _\eta \otimes \Bbbk _{\theta _{a,b}}$ as a representation over $\mathcal {F}_n \mathcal {O}$. The proof now follows by [40, Proposition 5.1.15] or [68, Lemma 8]. The final sentence of the result follows by the observation that $\Bbbk _{\varphi _n} = \Bbbk _{\chi }$ as $\mathcal {F}_n \mathcal {O}$-modules. □

The reducibility of a module is preserved when taking the tensor product with a one-dimensional representation of a Lie algebra. Thus, by Lemma 6.22, the irreducibility of the universal Whittaker module is completely governed by the local function η.

#### Corollary 6.23

Let $a,b \in \Bbbk$. Then, the $\mathcal {O}$-module ${{\,\textrm{Ind}\,}}^{\mathcal {O}}_{\mathcal {F}_n \mathcal {O}} \Bbbk _{\chi _{a,b}}$ is irreducible if and only if ${{\,\textrm{Ind}\,}}^{\mathcal {O}}_{\mathcal {F}_n \mathcal {O}} \Bbbk _\eta$ is irreducible. Consequently, the universal Whittaker module $M_{\varphi _n} = {{\,\textrm{Ind}\,}}_{\mathcal {F}_n \mathcal {O}}^{\mathcal {O}} \Bbbk _\chi$ is irreducible if and only if ${{\,\textrm{Ind}\,}}^{\mathcal {O}}_{\mathcal {F}_n \mathcal {O}} \Bbbk _{\chi _{1,1}}$ is irreducible. □

#### Remark 6.24

Corollary 6.23 implies that reducibility of the universal Whittaker module of type $\varphi _n$ is equivalent to the reducibility of the universal Whittaker module of type $\varphi '_n :={ \left. \hspace{0.0pt}\chi _{1,1} \phantom {\big |} \right| _{\mathcal {F}_n \mathcal {O}} }$.

We are now ready to complete the proof of Theorem 6.6.

#### Proof of Theorem 6.6

First, assume that $\lambda _{2} \ne 4 \lambda _0$ (if n=0) or that $\lambda _{2n + 1} \ne 4 \lambda _{2n - 1}$ or $\lambda _{2n + 2} \ne 4\lambda _{2n}$ (if n≥1). By Corollary 6.23, $M_{\varphi _n}$ is irreducible if and only if ${{\,\textrm{Ind}\,}}_{\mathcal {F}_n \mathcal {O}}^{\mathcal {O}} \Bbbk _{\chi _{1,1}}$ is irreducible. Now, Proposition 6.16 implies the following:

- If n=0, then $$\alpha _1 \ne 0$$.
- If n≥1, then $\alpha _{2n}$ and $\alpha _{2n+1}$ are not simultaneously 0.

By Corollary 6.20, we know that $\mathfrak {p}_{\chi _{1,1}} = \mathcal {F}_n \mathcal {O}$ is a polarisation of $\chi _{1,1}$. Furthermore, the order of each one-point local function which makes up $\chi _{1,1}$ is at least 1. Thus, Theorem 6.21 implies that ${{\,\textrm{Ind}\,}}_{\mathcal {F}_n \mathcal {O}}^{\mathbb {W}_1} \Bbbk _{\chi _{1,1}}$ is an irreducible $\mathbb {W}_1$-module. It is easy to see that

$$\begin{aligned} {{\,\textrm{Ind}\,}}_{\mathcal {F}_n \mathcal {O}}^{\mathbb {W}_1} \Bbbk _{\chi _{1,1}} = {{\,\textrm{Ind}\,}}_{\mathcal {O}}^{\mathbb {W}_1}({{\,\textrm{Ind}\,}}_{\mathcal {F}_n \mathcal {O}}^{\mathcal {O}} \Bbbk _{\chi _{1,1}}), \end{aligned}$$

so the irreducibility of ${{\,\textrm{Ind}\,}}_{\mathcal {F}_n \mathcal {O}}^{\mathbb {W}_1} \Bbbk _{\chi _{1,1}}$ as a $\mathbb {W}_1$-module implies the irreducibility of ${{\,\textrm{Ind}\,}}_{\mathcal {F}_n \mathcal {O}}^{\mathcal {O}} \Bbbk _{\chi _{1,1}}$ as an $\mathcal {O}$-module. We therefore conclude that $M_{\varphi _n}$ is irreducible.

For the converse, the case n=0 follows from Lemma 6.7 and the case n≥1 follows from Lemma 6.9. □

### Whittaker modules for the centreless BCCA

We move on to the study of Whittaker modules over the full centreless BCCA. Although these may seem more complicated, since the BCCA is a larger Lie algebra than $\mathcal {O}$, we will see that the conditions governing the reducibility of the universal Whittaker modules over $\mathfrak {b}$ are much simpler, and our proof is more elementary and direct. This is thanks to the abelian subalgebra $\mathcal {P}$ of $\mathfrak {b}$. As we did in Sect. 6.1, we start by defining Whittaker functions on $\mathfrak {b}$.

#### Definition 6.25

A *Whittaker function on* $\mathfrak {b}$ is a Lie algebra homomorphism $\psi _n :\mathcal {F}_n\mathfrak {b}\rightarrow \Bbbk$ for some $n \in \mathbb {N}$.

The following result is an immediate consequence of Lemma 5.5 and Proposition 5.7.

#### Lemma 6.26

A Whittaker function $\psi _n :\mathcal {F}_n\mathfrak {b}\rightarrow \Bbbk$ is determined by its values on $\mathcal {U}_n, \dots , \mathcal {U}_{2n+2}$ and $\mathcal {V}_n, \dots , \mathcal {V}_{2n}$. Once these values are determined, we have

$$\begin{aligned} \psi _n(\mathcal {U}_{m + 2}) = 4\psi _n(\mathcal {U}_m), \quad \psi _n(\mathcal {V}_m) = 0 \end{aligned}$$

for all m≥2n+1. □

We now define the universal Whittaker modules for the full centreless BCCA and then proceed to study their reducibility.

#### Definition 6.27

Let $\psi _n :\mathcal {F}_n\mathfrak {b}\rightarrow \Bbbk$ be a Whittaker function, and $\Bbbk _{\psi _n} = \Bbbk 1_{\psi _n}$ be the one-dimensional $\mathcal {F}_n\mathfrak {b}$-module defined by $\psi _n$, in other words,

$$\begin{aligned} x \cdot 1_{\psi _n} = \psi _n(x)1_{\psi _n} \end{aligned}$$

for all $x \in \mathcal {F}_n\mathfrak {b}$. Define the *universal Whittaker module for* $\mathfrak {b}$ *of type* $\psi _n$ to be the induced module

$$\begin{aligned} M_{\psi _n} :={{\,\textrm{Ind}\,}}_{\mathcal {F}_n\mathfrak {b}}^{\mathfrak {b}} \Bbbk _{\psi _n}. \end{aligned}$$

As in Remark 6.5, the $\mathfrak {b}$-modules $M_{\psi _n}$ for n≥1 are Whittaker modules, but $M_{\psi _0}$ are not Whittaker modules.

The following result is the main theorem of this subsection.

#### Theorem 6.28

Let $n \in \mathbb {N}$, and let $\psi _n :\mathcal {F}_n\mathfrak {b}\rightarrow \Bbbk$ be a Whittaker function. If n=0, then $M_{\psi _0}$ is irreducible if and only if $\psi _0(\mathcal {V}_0) \ne 0$. If n≥1, then $M_{\psi _n}$ is irreducible if and only if $\psi _n(\mathcal {V}_{2n}) \ne 0$ or $\psi _n(\mathcal {V}_{2n - 1}) \ne 0$.

We first prove the easier direction of Theorem 6.28: we construct a nonzero proper submodule of $M_{\psi _n}$ when the conditions of Theorem 6.28 are not satisfied. We first consider the case n=0.

#### Lemma 6.29

Suppose n=0 and $\psi _0(\mathcal {V}_0) = 0$. Then, $U(\mathfrak {b})\mathcal {V}_{-1} \cdot 1_{\psi _0}$ is a proper submodule of $M_{\psi _0}$.

#### Proof

By Lemma 6.26, we have $\psi _0(\mathcal {V}_m) = 0$ for all m≥0. In other words, $\mathcal {V}_m \cdot 1_{\psi _0} = 0$ for all m≥0. Let $w :=\mathcal {V}_{-1} \cdot 1_{\psi _n}$. It is clear that $\mathcal {V}_m \cdot w = 0$ for all m≥0, since the $\mathcal {V}_k$ commute. Furthermore, for m≥0, we have

$$\begin{aligned} \mathcal {U}_m \cdot w&= \mathcal {U}_m \mathcal {V}_{-1} \cdot 1_{\psi _0} = [\mathcal {U}_m,\mathcal {V}_{-1}] \cdot 1_{\psi _0} + \mathcal {V}_{-1} \mathcal {U}_m \cdot 1_{\psi _0} \\&= \Big ((m + 2)\mathcal {V}_{m + 1} - 4(m + 1)\mathcal {V}_{m - 1}\Big ) \cdot 1_{\psi _0} + \psi _0(\mathcal {U}_m) w \\&= -4(m + 1)\mathcal {V}_{m - 1} \cdot 1_{\psi _0} + \psi _0(\mathcal {U}_m) w, \end{aligned}$$

since m+1≥0, where we used that $\mathcal {V}_k \cdot w = 0$ for all k≥0. If m≥1, then the above implies that $\mathcal {U}_m \cdot w = \psi _0(\mathcal {U}_m) w$. On the other hand, if m=0, then

$$\begin{aligned} \mathcal {U}_0 \cdot w = (\psi _0(\mathcal {U}_0) - 4)w. \end{aligned}$$

It follows that kw is a one-dimensional $\mathcal {F}_0 \mathfrak {b}$-module, and thus, $U(\mathfrak {b}) \cdot w$ is a proper submodule of $M_{\psi _0}$. □

#### Remark 6.30

In the notation from the proof of Lemma 6.29, it is easy to see that $U(\mathfrak {b}) \cdot w$ is isomorphic to $M_{\psi _0'}$, where $\psi _0' :\mathcal {F}_0 \mathfrak {b}\rightarrow \Bbbk$ is given by $\psi _0'(\mathcal {U}_0) = \psi _0(\mathcal {U}_0) - 4$, $\psi _0'(\mathcal {U}_m) = \psi _0(\mathcal {U}_m)$ for m≥1, and $\psi _0'(\mathcal {V}_m) = 0$ for m≥0. Therefore, Lemma 6.29 implies that there is chain of submodules

$$\begin{aligned} M_{\psi _0} \supseteq U(\mathfrak {b})\mathcal {V}_{-1} \cdot 1_{\psi _0} \supseteq U(\mathfrak {b})\mathcal {V}_{-1}^2 \cdot 1_{\psi _0} \supseteq U(\mathfrak {b})\mathcal {V}_{-1}^3 \cdot 1_{\psi _0} \supseteq \dots . \end{aligned}$$

Next, we construct an analogous submodule to the one in Lemma 6.29 in the case n≥1.

#### Lemma 6.31

Let n≥1. If $\psi _n(\mathcal {V}_{2n}) = \psi _n(\mathcal {V}_{2n - 1}) = 0$, then $U(\mathfrak {b}) \mathcal {V}_{n - 1} \cdot 1_{\varphi _n}$ is a proper submodule of $M_{\psi _n}$ isomorphic to $M_{\psi _n}$ itself.

#### Proof

Let $w :=\mathcal {V}_{n - 1} \cdot 1_{\psi _n}$. For m≥n, we have

$$\begin{aligned} \mathcal {U}_m \cdot w&= \mathcal {U}_m \mathcal {V}_{n - 1} \cdot 1_{\psi _n} = [\mathcal {U}_m,\mathcal {V}_{n - 1}] \cdot 1_{\psi _n} + \mathcal {V}_{n - 1} \mathcal {U}_m \cdot 1_{\psi _n} \\&= \Big ((m - n + 2)\mathcal {V}_{n + m + 1} - 4(m - n + 1)\mathcal {V}_{n + m - 1}\Big ) \cdot 1_{\psi _n} + \psi _n(\mathcal {U}_m) w \\&=\psi _n(\mathcal {U}_m) w, \end{aligned}$$

since n+m-1≥2n-1, where we used that $\mu _k = 0$ for all k≥2n-1. Similarly,

$$\begin{aligned} \mathcal {V}_m \cdot w = \mathcal {V}_m \mathcal {V}_{n - 1} \cdot 1_{\psi _n} = \mathcal {V}_{n - 1} \mathcal {V}_m \cdot 1_{\psi _n} = \psi _n(\mathcal {V}_m) w, \end{aligned}$$

where we used that $\mathcal {V}_m$ and $\mathcal {V}_{n - 1}$ commute.

The above shows that kw is a one-dimensional $\mathcal {F}_n \mathfrak {b}$-module isomorphic to $\Bbbk _{\psi _n}$. It is now easy to see that $U(\mathfrak {b}) \cdot w$ is isomorphic to $M_{\psi _n}$. □

For the remainder of this section, we assume that at least one of $\psi _n(\mathcal {V}_{2n})$ and $\psi _n(\mathcal {V}_{2n - 1})$ is nonzero (if n≥1) or that $\psi _0(\mathcal {V}_0) \ne 0$ (if n=0). As a result, we introduce the following notation.

#### Notation 6.32

Fix $n \in \mathbb {N}$ and a Whittaker function $\psi _n :\mathcal {F}_n \mathfrak {b}\rightarrow \Bbbk$. For m≥n, define

$$\begin{aligned} \lambda _m :=\psi _n(\mathcal {U}_m), \quad \mu _m :=\psi _n(\mathcal {V}_m). \end{aligned}$$

If n≥1, let $\kappa \in \{2n - 1, 2n\}$ be maximal such that $\mu _\kappa \ne 0$. If n=0, let κ=0.

By Lemma 6.26, we have $\lambda _{m + 2} = 4\lambda _{m}$ and $\mu _m = 0$ for all m≥2n+1.

Having introduced the necessary notation, we now proceed to prove the other direction of Theorem 6.28. To achieve this, we need to show that we can use actions of $\mathfrak {b}$ to get $1_{\varphi _n}$ from any nonzero element of $M_{\psi _n}$ when $\mu _{2n}\ne 0$ or $\mu _{2n-1}\ne 0$. We do this by successively removing $\mathcal {U}_i$ and $\mathcal {V}_j$ from elements of $U(\mathfrak {b})$. First, we prove that $\mathcal {V}_m$ annihilates elements in $M_{\psi _n}$, which do not contain $\mathcal {U}_{-1}$, provided m>κ. Recall that $\mathcal {P}= {{\,\textrm{span}\,}}\{\mathcal {V}_k| k \ge -1 \}$ is an abelian subalgebra of $\mathfrak {b}$.

#### Lemma 6.33

Let $x = \mathcal {U}_{r_1} \dots \mathcal {U}_{r_\ell } v \in M_{\psi _n}$, where $v \in U(\mathcal {P}) \cdot 1_{\psi _n}$ and $0 \le r_1 \le r_2 \le \dots \le r_\ell \le n - 1$. Then, $\mathcal {V}_{\kappa + m} \cdot x = 0$ for all m≥1.

#### Proof

The result is clearly true if n=0, so assume that n≥1. We proceed by induction on ℓ, the base case ℓ=0 being trivial. For the induction step, assume that the statement holds for some $\ell \in \mathbb {N}$. Let $x = \mathcal {U}_{r_0} \mathcal {U}_{r_1} \dots \mathcal {U}_{r_\ell } v$, where $v \in U(\mathcal {P}) \cdot 1_{\psi _n}$ and $0 \le r_0 \le r_1 \le \dots \le r_\ell \le n - 1$. For all m≥1, we have

$$\begin{aligned} \mathcal {V}_{\kappa + m} \cdot x&= \mathcal {V}_{\kappa + m} \mathcal {U}_{r_0} \mathcal {U}_{r_1} \dots \mathcal {U}_{r_\ell } v \\&= [\mathcal {V}_{\kappa + m},\mathcal {U}_{r_0}]\mathcal {U}_{r_1} \dots \mathcal {U}_{r_\ell } v + \mathcal {U}_{r_0} \mathcal {V}_{\kappa + m} \mathcal {U}_{r_1} \dots \mathcal {U}_{r_\ell } v \\&= \Big ((\kappa + m - r_0 - 1)\mathcal {V}_{\kappa + m + r_0 + 2} - 4(\kappa + m - r_0)\mathcal {V}_{\kappa + m + r_0}\Big )\mathcal {U}_{r_1} \dots \mathcal {U}_{r_\ell } v \\&= 0, \end{aligned}$$

where we used the induction hypothesis in the last two equalities. □

Next, we extend Lemma 6.33 to the situation where the element of $M_{\psi _n}$ contains $\mathcal {U}_{-1}$.

#### Lemma 6.34

Let $x \in U(\mathcal {F}_0 \mathcal {O}\ltimes \mathcal {P}) \cdot 1_{\psi _n}$. Then $\mathcal {V}_{\kappa + m} \cdot \mathcal {U}_{-1}^N x = 0$ if m>N.

#### Proof

Follows via an easy induction on *N*, with Lemma 6.33 as the base case. □

In the following result, we show that the action $\mathcal {V}_{\kappa + N} \cdot \mathcal {U}_{-1}^N x$ results in the complete removal of $\mathcal {U}_{-1}$, in other words, that $\mathcal {V}_{\kappa + N} \cdot \mathcal {U}_{-1}^N x$ is a nonzero element of $U(\mathcal {F}_0 \mathcal {O}\ltimes \mathcal {P}) \cdot 1_{\psi _n}$, where $x \in U(\mathcal {F}_0 \mathcal {O}\ltimes \mathcal {P}) \cdot 1_{\psi _n}$.

#### Proposition 6.35

Let $x \in U(\mathcal {F}_0 \mathcal {O}\ltimes \mathcal {P}) \cdot 1_{\psi _n} \setminus \{0\}$. Then, for any N≥1, there exists $c \in \Bbbk \setminus \{0\}$ such that

$$\begin{aligned} \mathcal {V}_{\kappa + N} \cdot \mathcal {U}_{-1}^N x = c x + {\text {lower degree terms in }} U(\mathcal {F}_0 \mathcal {O}\ltimes \mathcal {P}) \ne 0, \end{aligned}$$

where *lower degree terms* means terms with lower degree than *x* under the PBW filtration on $U(\mathfrak {b})$.

#### Proof

We proceed by induction on *N*. First, consider the base case N=0:

$$\begin{aligned} \mathcal {V}_\kappa \cdot x = \mu _\kappa x + {\text {lower degree terms in }} U(\mathcal {F}_0 \mathcal {O}\ltimes \mathcal {P}), \end{aligned}$$

which is nonzero.

For the induction step, assume the statement is true for some $N \in \mathbb {N}$. Then

$$\begin{aligned} \mathcal {V}_{\kappa + N + 1} \cdot \mathcal {U}_{-1}^{N + 1} x&= [\mathcal {V}_{\kappa + N + 1}, \mathcal {U}_{-1}] \mathcal {U}_{-1}^N x + \mathcal {U}_{-1} \mathcal {V}_{\kappa + N + 1} \mathcal {U}_{-1}^N x \\&= \Big ((\kappa + N + 1)\mathcal {V}_{\kappa + N + 2} - 4(\kappa + N + 2)\mathcal {V}_{\kappa + N}\Big ) \mathcal {U}_{-1}^N x \\&= -4(\kappa + N + 2)\mathcal {V}_{\kappa + N} \mathcal {U}_{-1}^N x, \end{aligned}$$

where we used Lemma 6.34 in the last two equalities. By the induction hypothesis, it follows that $\mathcal {V}_{\kappa + N + 1} \cdot \mathcal {U}_{-1}^{N + 1} x \in U(\mathcal {F}_0 \mathcal {O}\ltimes \mathcal {P}) \setminus \{0\}$, which concludes the proof. □

Having shown how $\mathcal {U}_{-1}$ can be removed from any element of $U(\mathfrak {b})$, we now proceed to also remove $\mathcal {U}_0$.

#### Proposition 6.36

Suppose n≥1. Then the following hold.

1. Let $$x \in U(\mathcal {F}_1 \mathcal {O}\ltimes \mathcal {P}) \cdot 1_{\psi _n} \setminus \{0\}$$. Then $$\mathcal {V}_\kappa \cdot x = \mu _\kappa x$$.
2. Let $x \in U(\mathcal {F}_1 \mathcal {O}\ltimes \mathcal {P}) \cdot 1_{\psi _n} \setminus \{0\}$. Then there exists $c \in \Bbbk \setminus \{0\}$ such that $(\mathcal {V}_{\kappa }-\mu _\kappa )^N \cdot \mathcal {U}_{0}^N x = cx$.

#### Proof

For (1), it suffices to consider $x = \mathcal {U}_{r_1} \dots \mathcal {U}_{r_\ell }y$, where $y \in U(\mathcal {P}) \cdot 1_{\psi _n}$ and $1 \le r_1 \le r_2 \le \dots \le r_\ell \le n - 1$. The result is clearly true for n=1, so assume that n≥2. We proceed by induction on ℓ, the case ℓ=0 being trivial. For the induction step, assume that the statement is true for some $\ell \in \mathbb {N}$. Then

$$\begin{aligned} \mathcal {V}_\kappa \cdot \mathcal {U}_{r_0} \mathcal {U}_{r_1} \dots \mathcal {U}_{r_\ell } y = \mathcal {U}_{r_0} \mathcal {V}_\kappa \mathcal {U}_{r_1} \dots \mathcal {U}_{r_\ell } y + [\mathcal {V}_\kappa ,\mathcal {U}_{r_0}]\mathcal {U}_{r_1} \dots \mathcal {U}_{r_\ell } y. \end{aligned}$$

By the induction hypothesis, we have

$$\begin{aligned} \mathcal {V}_\kappa \mathcal {U}_{r_1} \dots \mathcal {U}_{r_\ell } y = \mu _\kappa \mathcal {V}_\kappa \mathcal {U}_{r_1} \dots \mathcal {U}_{r_\ell } y. \end{aligned}$$

Therefore,

$$\begin{aligned} \mathcal {V}_\kappa \cdot \mathcal {U}_{r_0} \mathcal {U}_{r_1} \dots \mathcal {U}_{r_\ell } y = \mu _\kappa \mathcal {U}_{r_0} \mathcal {U}_{r_1} \dots \mathcal {U}_{r_\ell } y + \Big ((\kappa - r_0 - 1)\mathcal {V}_{\kappa + r_0 + 2} - 4(\kappa - r_0)\mathcal {V}_{\kappa + r_0}\Big )\mathcal {U}_{r_1} \dots \mathcal {U}_{r_\ell } y. \end{aligned}$$

By Lemma 6.33, it follows that $\Big ((\kappa - r_0 - 1)\mathcal {V}_{\kappa + r_0 + 2} - 4(\kappa - r_0)\mathcal {V}_{\kappa + r_0}\Big )\mathcal {U}_{r_1} \dots \mathcal {U}_{r_\ell } y = 0$, since $r_0 \ge 1$. We conclude that $\mathcal {V}_\kappa \cdot \mathcal {U}_{r_0} \mathcal {U}_{r_1} \dots \mathcal {U}_{r_\ell } y = \mu _\kappa \mathcal {U}_{r_0} \mathcal {U}_{r_1} \dots \mathcal {U}_{r_\ell } y$, which concludes the proof of (1).

For (2), the proof is similar to that of Proposition 6.35, using (1). □

The next result shows how to remove all the other $\mathcal {U}_i$ from an element of $U(\mathfrak {b})$ by successively acting by $(\mathcal {V}_m - \mu _m)$ for some appropriately chosen $m \in \mathbb {N}$. Of course, if n=0 or n=1, then we have already removed all $\mathcal {U}_i$ in the previous results, so we do not need to consider these cases.

#### Proposition 6.37

Suppose n≥2, and let $x = \mathcal {U}_{q_1}^{p_{1}} \dots \mathcal {U}_{q_k}^{p_{k}} y$, where $y \in U(\mathcal {P}) \cdot 1_{\psi _n}$ and $1 \le q_1< q_2< \dots < q_k \le n - 1$. Then

$$(\mathcal {V}_m - \mu _m) \cdot x = {\left\{ \begin{array}{ll} -4 p_1 \mu _\kappa (\kappa - 2q_1) \mathcal {U}_{q_1}^{p_{1} - 1} \dots \mathcal {U}_{q_k}^{p_{k}} y, & {\text {if }} m = \kappa - q_1, \\ 0, & {\text {if }} m > \kappa - q_1. \end{array}\right. }$$

#### Proof

Rewrite *x* as

$$\begin{aligned} x = \mathcal {U}_{s_1} \dots \mathcal {U}_{s_\ell } y, \end{aligned}$$

where $1 \le s_1 \le s_2 \le \dots \le s_\ell \le n - 1$. Of course, we have $s_1 = q_1$. For m≥n, we have

$$\begin{aligned} \mathcal {V}_m \cdot x&= \mathcal {V}_m \mathcal {U}_{s_1} \dots \mathcal {U}_{s_\ell } y \\&= \mathcal {U}_{s_1} \dots \mathcal {U}_{s_\ell } \mathcal {V}_m y + [\mathcal {V}_m,\mathcal {U}_{s_1} \dots \mathcal {U}_{s_\ell }]y \\&= \mu _m x + \sum _{j = 1}^\ell \mathcal {U}_{s_1} \dots \mathcal {U}_{s_{j - 1}} [\mathcal {V}_m,\mathcal {U}_{s_j}] \mathcal {U}_{s_{j + 1}} \dots \mathcal {U}_{s_\ell }y \\&= \mu _m x + \sum _{j = 1}^\ell \mathcal {U}_{s_1} \dots \mathcal {U}_{s_{j - 1}} \Big ((m - s_j - 1)\mathcal {V}_{m + s_j + 2} - 4(m - s_j)\mathcal {V}_{m + s_j}\Big ) \mathcal {U}_{s_{j + 1}} \dots \mathcal {U}_{s_\ell }y. \end{aligned}$$

If $m + s_j > \kappa$, then the $j^{\text {th}}$ term of the above summation is zero, by Lemma 6.33. In particular, if $m > \kappa - s_1 = \kappa - q_1$, then the entire summation vanishes, and thus $\mathcal {V}_m \cdot x = \mu _m x$, as required.

Now suppose $m = \kappa - s_1 = \kappa - q_1$. By the above discussion, we can only get a contribution from the $j^{\text {th}}$ term of the above summation if $s_j = s_1$, since in this case we have $m + s_j = \kappa$. Therefore, switching back to the original notation for *x*, we get

$$\begin{aligned} \mathcal {V}_{\kappa - q_1} \cdot x = \mu _{\kappa - q_1} x - 4(\kappa - 2q_1) \sum _{i = 0}^{p_1 - 1} \mathcal {U}_{q_1}^i \mathcal {V}_\kappa \mathcal {U}_{q_1}^{p_1 - i - 1} \mathcal {U}_{q_2}^{p_2} \dots \mathcal {U}_{q_k}^{p_k} y. \end{aligned}$$

By Proposition 6.36(1), it follows that

$$\begin{aligned} \mathcal {V}_\kappa \cdot \mathcal {U}_{q_1}^{p_1 - i - 1} \mathcal {U}_{q_2}^{p_2} \dots \mathcal {U}_{q_k}^{p_k} y = \mu _\kappa \mathcal {U}_{q_1}^{p_1 - i - 1} \mathcal {U}_{q_2}^{p_2} \dots \mathcal {U}_{q_k}^{p_k} y, \end{aligned}$$

and thus

$$\begin{aligned} \mathcal {V}_{\kappa - q_1} \cdot x = \mu _{\kappa - q_1} x - 4 p_1 \mu _\kappa (\kappa - 2q_1) \mathcal {U}_{q_1}^{p_1 - 1} \mathcal {U}_{q_2}^{p_2} \dots \mathcal {U}_{q_k}^{p_k} y, \end{aligned}$$

which concludes the proof. □

The next result is a direct consequence of Propositions 6.35, 6.36, and 6.37.

#### Corollary 6.38

Let *M* be a nonzero submodule of $M_{\psi _n}$. Then, *M* contains a nonzero element in $U(\mathcal {P}) \cdot 1_{\psi _n}$. □

Thanks to Corollary 6.38, we can simply focus on elements of $M_{\psi _n}$ not containing any $\mathcal {U}_i$. Thankfully, the removal of $\mathcal {V}_j$ is much easier, since the $\mathcal {V}_j$ commute with each other.

#### Lemma 6.39

Let $y = \mathcal {V}_{q_1}^{p_1} \dots \mathcal {V}_{q_k}^{p_k} \cdot 1_{\psi _n} \in M_{\psi _n}$ with $-1 \le q_1< \dots < q_k \le n - 1$. Then

$$(\mathcal {U}_m - \lambda _m) \cdot y = {\left\{ \begin{array}{ll} -4 p_1 \mu _\kappa (\kappa - 2q_1) \mathcal {V}_{q_1}^{p_1 - 1} \mathcal {V}_{q_2}^{p_2} \dots \mathcal {V}_{q_k}^{p_k} \cdot 1_{\psi _n}, & {\text {if }} m = \kappa - q_1, \\ 0, & {\text {if }} m > \kappa - q_1. \end{array}\right. }.$$

#### Proof

Rewrite *y* as

$$\begin{aligned} y = \mathcal {V}_{s_1} \dots \mathcal {V}_{s_\ell } \cdot 1_{\psi _n}, \end{aligned}$$

where $-1 \le s_1 \le s_2 \le \dots \le s_\ell \le n - 1$ (and thus $s_1 = q_1$). We have

$$\begin{aligned} \mathcal {U}_m \cdot y&= \mathcal {U}_m \mathcal {V}_{s_1} \dots \mathcal {V}_{s_\ell } \cdot 1_{\psi _n} = \mathcal {V}_{s_1} \dots \mathcal {V}_{s_\ell } \mathcal {U}_m \cdot 1_{\psi _n} + \sum _{j = 1}^\ell \mathcal {V}_{s_1} \dots \mathcal {V}_{s_{j - 1}} [\mathcal {U}_m,\mathcal {V}_{s_j}] \mathcal {V}_{s_{j + 1}} \dots \mathcal {V}_{s_\ell } \cdot 1_{\psi _n} \\&= \lambda _m y + \sum _{j = 1}^\ell \mathcal {V}_{s_1} \dots \mathcal {V}_{s_{j - 1}} \Big ((m - s_j + 1)\mathcal {V}_{m + s_j + 2} - 4(m - s_j)\mathcal {V}_{m + s_j}\Big ) \mathcal {V}_{s_{j + 1}} \dots \mathcal {V}_{s_\ell } \cdot 1_{\psi _n} \end{aligned}$$

Suppose $m = \kappa - q_1 = \kappa - s_1$. Then, $m + s_j > \kappa$ if $s_j > s_1$. Therefore, we only get a contribution from the $j^{\text {th}}$ term in the above summation if $s_j = s_1$, since in this case we have $m + s_j = \kappa$. It follows that

$$\begin{aligned} \mathcal {U}_{\kappa - q_1} \cdot y = \lambda _{\kappa - q_1} y - 4 p_1 \mu _\kappa (\kappa - 2q_1) \mathcal {V}_{q_1}^{p_1 - 1} \mathcal {V}_{q_2}^{p_2} \dots \mathcal {V}_{q_k}^{p_k} \cdot 1_{\psi _n}, \end{aligned}$$

which concludes the proof. The case where $m > \kappa - q_1$ follows similarly. □

We are now ready to prove Theorem 6.28.

#### Proof of Theorem 6.28

One direction of the statement is simply Lemmas 6.29 and 6.31. For the converse, assume that $\mu _{2n} \ne 0$ or $\mu _{2n - 1} \ne 0$, and let *M* be a nonzero submodule of $M_{\psi _n}$. Using Corollary 6.38, choose an element $w \in M \cap (U(\mathcal {P}) \cdot 1_{\psi _n}) \setminus \{0\}$. Now apply Lemma 6.39 to *w* repeatedly to deduce that *M* contains a nonzero scalar multiple of the generating vector $1_{\psi _n}$ of $M_{\psi _n}$. Therefore, $M = M_{\psi _n}$, which concludes the proof. □

#### Remark 6.40

One may wonder about using the methods of [32] to prove Theorems 6.6 and 6.28. The main issue is that $\mathcal {F}_n \mathcal {O}$ and $\mathcal {F}_n \mathfrak {b}$ are not ideals of $\mathcal {O}$ and $\mathfrak {b}$ for n≥0, making it impossible to apply their results directly, since quasi-Whittaker functions must be defined on ideals of the Lie algebra (recall Definition 2.12). But $\mathcal {F}_n \mathcal {O}$ and $\mathcal {F}_n \mathfrak {b}$ are ideals of $\mathcal {F}_0 \mathcal {O}$ and $\mathcal {F}_0 \mathfrak {b}$, so one could get some irreducibility results by inducing from $\mathcal {F}_n \mathcal {O}$ or $\mathcal {F}_n \mathfrak {b}$ to $\mathcal {F}_0 \mathcal {O}$ or $\mathcal {F}_0 \mathfrak {b}$ and analysing the Whittaker annihilator, as is done in [32]. However, deducing the irreducibility of the universal Whittaker module $M_{\varphi _n}$ or $M_{\psi _n}$ from the irreducibility of ${{\,\textrm{Ind}\,}}_{\mathcal {F}_n \mathcal {O}}^{\mathcal {F}_0 \mathcal {O}} \Bbbk _{\varphi _n}$ or ${{\,\textrm{Ind}\,}}_{\mathcal {F}_n \mathfrak {b}}^{\mathcal {F}_0 \mathfrak {b}} \Bbbk _{\psi _n}$ is a non-trivial task which would require similar analysis to our proof of Theorem 6.28. For this reason, we opted for a more direct proof in the case of $\mathfrak {b}$, and a proof using the orbit method for $\mathcal {O}$.

## Future work

**Fig. 1:**
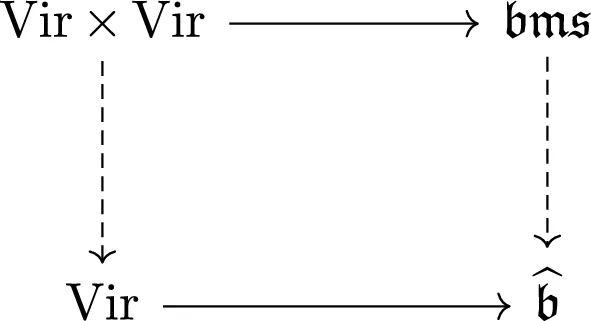
Diagram summarising the relations between ${{\,\textrm{Vir}\,}}$, $\mathfrak {bms}$ and $\widehat{\mathfrak {b}}$. The vertical dashed arrows denote restrictions, while the horizontal solid ones denote Lie algebra contractions

In summary, this paper provides new insights into the algebraic structure of the BCCA and studies some of its representations, namely restrictions of representations of larger Lie algebras and Whittaker modules. We hope our work serves as a useful starting point for further exploration of the BCCA and the related mathematical and physical concepts that one would encounter. We conclude this paper by presenting some of these future research directions:

1. The BCCA can be obtained as the Lie algebra contraction of ${{\,\textrm{Vir}\,}}$ (see [12, Equation (8)]). Hence, one could study $\mathfrak {b}$-modules obtained as contractions of ${{\,\textrm{Vir}\,}}$-modules. Contractions at the level of representations are essential in Carrollian physics [34], but there is still much that is unclear in this regard. For instance, the contractions of ${{\,\textrm{Vir}\,}}\times {{\,\textrm{Vir}\,}}$-modules built from “mode algebras” or free fields (i.e. *BC*-systems, Heisenberg algebra, etc.) have been considered in the physics literature^4^ (see, for example, [54, Sections 5.1.2 and 5.2], [8, Section 4] and [9, Section 3.2.1]). However, there are still technical subtleties (e.g. issues with “normal-ordering”) when it comes to contracting these infinite-dimensional representations that need to be addressed. Attempting to construct sensible representations of the BCCA as contractions of representations of the Virasoro algebra may help address some of these subtleties (see, for instance, [74] for some work in this direction on VOA modules). They should also help guide us towards the quantisation of the tensionless open string since we expect such representations to describe the possible spectra of this string. It would be particularly interesting if representations constructed in this manner belong to one of the classes of $\widehat{\mathfrak {b}}$-modules discussed in this paper.
2. The above line of enquiry leads naturally to the consideration of restrictions of $\mathfrak {bms}$-modules that are vertex operator algebra (VOA) modules [24, 41, 44] (see [84] for the first appearance of $\mathfrak {bms}$-modules^5^ in mathematics), such as the $\mathfrak {bms}$-module formed from two fermionic *BC* systems [42, 45, 48], each of conformal weight (2,-1). This module is also known as the semi-infinite wedge or fermionic Fock representation [42, 43], denoted $\Lambda ^{\boldsymbol{\cdot }}_\infty (\mathfrak {bms})$. For one of the three vacua of closed tensionless string theory, $\Lambda ^{\boldsymbol{\cdot }}_\infty (\mathfrak {bms})$ is the so-called ghost sector of the theory that appears during Becchi–Rouet–Stora–Tyutin (BRST) quantisation [26–28, 79]. Thus, understanding the restriction of $\Lambda ^{\boldsymbol{\cdot }}_\infty (\mathfrak {bms})$ to $\mathfrak {b}$ (note that it was shown in [46, Theorem 3.19] that $c_M = 0$ for such a $\mathfrak {bms}$-module) could be the first step towards formulating a BRST quantisation procedure for the tensionless open string, or at least one of its vacua (if there exist many). It may also help formulate a generalised notion of semi-infinite cohomology for Lie algebras without integer-grading.
3. In general, studying the restrictions and, if possible, contractions of VOA modules over $\mathfrak {bms}$, ${{\,\textrm{Vir}\,}}\times {{\,\textrm{Vir}\,}}$ and ${{\,\textrm{Vir}\,}}$ would provide field-theoretic insights for the tensionless open string, possibly informing us about its spectrum, quantisation, etc. Some of these modules are well-known examples of highest weight modules, so one should expect that after carefully dealing with normal-ordering ambiguities, their restriction to $\widehat{\mathfrak {b}}$-modules does indeed give rise to the “almost free” modules discussed in Sect. 3. Overall, together with the work in Sect. 3, we expect these two strands of exploration to aid in understanding the commutative diagram given in Fig. 1 at the level of Lie algebra representations while providing new field-theoretic realisations of the BCCA, which would be very informative in deducing the possible spectra of quantised tensionless open strings.
4. Proving Conjecture 3.17 could not only aid the search for field-theoretic realisations of the BCCA, but also result in the development of novel methods to check the (in)decomposability of Lie algebra modules and/or study representations of infinite-dimensional non-$\mathbb {Z}$-graded Lie algebras.
5. A similar programme to the one in this paper can be undertaken for higher spin BCCAs [12, Equation (26)]. Following our notation in Sect. 2, the higher spin BCCAs form the one-parameter family of Lie algebras $\mathcal {O}\ltimes \mathcal {P}_b$ (and their central extensions), where *b* is usually taken to be an integer in physics, though the programme undertaken in this paper could be done for arbitrary $b \in \Bbbk$.
6. One could compute the low-dimensional cohomology of the two-parameter family of higher spin BCCAs, analogous to what was done for the Lie algebras $\mathcal {W}(a,b) :=\mathcal {W}\ltimes I (a,b)$ (see Definition 2.2) in [50]. This would include a computation of the central extensions of $\mathfrak {b}$.
7. One can go beyond the methods in this paper to construct modules over $\widehat{\mathfrak {b}}$ or the centrally closed version of $\mathfrak {b}$, if it is proven not to be $\widehat{\mathfrak {b}}$. Such representations would inevitably be vital in tensionless open string theory and other fields of theoretical physics.
8. Finally, our work can be extended to the supersymmetric versions of the BCCA. Two such algebras, dubbed the *homogenous and inhomogeneous boundary superconformal Carrollian algebras (BSCCAs)*, were obtained in [11] via similar constructions to the ones in [12]. The study of modules over the homogenous BSCCA should serve as an invaluable stepping stone towards understanding the spectrum of the tensionless open superstring.

## Data Availability

The authors declare that this work has no associated data.

## More subalgebras of the Witt algebra

Given the results of Sect. 4.2, it is natural to ask if we get similar subalgebras of the Witt algebra by choosing generators

$$\begin{aligned} \mathcal {X}_1 :=L_1 - \lambda L_{-1}, \quad \mathcal {X}_2 :=L_2 - \mu L_{-2} \end{aligned}$$

for some $\lambda , \mu \in \Bbbk ^*$. As the following lemma shows, these elements generate the entirety of $\mathcal {W}$ unless $\mu = \lambda ^2$.

### Lemma A.1

If $\mu \ne \lambda ^2$, then $\mathcal {X}_1$ and $\mathcal {X}_2$ generate $\mathcal {W}$.

### Proof

This proof is most easily done with the help of a computer. As such, we omit the tedious computations and instead focus on the main arguments of the proof.

Let $\mathfrak {g}$ be the subalgebra of $\mathcal {W}$ generated by $\mathcal {X}_1$ and $\mathcal {X}_2$. For n≥3, inductively define $\mathcal {X}_n :=\frac{1}{n - 2}[\mathcal {X}_{n - 1},\mathcal {X}_1] \in \mathfrak {g}$. In this way, we have $\mathcal {X}_n = L_n + {\text {lower degree terms}}$ for all n≥1. There is another way to generate an element of degree 5: we have $\mathcal {Y}_5 :=[\mathcal {X}_3,\mathcal {X}_2] \in \mathfrak {g}$. It can be checked that

$$\begin{aligned} \mathcal {X}_5 - \mathcal {Y}_5 = -\frac{16}{3}\lambda L_3 + {\text {lower degree terms}}, \end{aligned}$$

so we define $\mathcal {Y}_3 :=-\frac{3}{16\lambda }(\mathcal {X}_5 - \mathcal {Y}_5) \in \mathfrak {g}$. Similarly, we can check that

$$\begin{aligned} \mathcal {X}_3 - \mathcal {Y}_3 = -\frac{45}{16\lambda }(\lambda ^2 - \mu )L_1 + {\text {lower degree terms}}. \end{aligned}$$

Since we assumed that $\mu \ne \lambda ^2$, we know that $\mathcal {X}_3 - \mathcal {Y}_3 \ne 0$ (in fact, $\mathcal {X}_3 = \mathcal {Y}_3$ if and only if $\mu = \lambda ^2$). Thus, define $\mathcal {Y}_1 :=\frac{16\lambda }{45(\lambda ^2 - \mu )}(\mathcal {X}_3 - \mathcal {Y}_3) \in \mathfrak {g}$. Continuing this process, we have

$$\begin{aligned} \mathcal {X}_1 - \mathcal {Y}_1 = -\frac{16}{15}\lambda L_{-1} + {\text {lower degree terms}}, \end{aligned}$$

so we define $\mathcal {Y}_{-1} :=-\frac{15}{16\lambda }(\mathcal {X}_1 - \mathcal {Y}_1) \in \mathfrak {g}$. Specifically, we have

$$\begin{aligned} \mathcal {Y}_{-1} = L_{-1} + \frac{3\mu }{16\lambda } L_{-3} + \frac{\mu }{16} L_{-5}. \end{aligned}$$

We also have the following element of $\mathfrak {g}$:

$$\begin{aligned} \mathcal {Z} :=[\mathcal {X}_1,\mathcal {Y}_{-1}] = 2\lambda L_0 + \frac{3}{4} \mu L_{-2} - \frac{\lambda ^2 \mu }{4} L_{-6}. \end{aligned}$$

Notice that $\mathcal {W}_{\le 1} :={{\,\textrm{span}\,}}\{L_n \mid n \le 1\}$ is a subalgebra of $\mathcal {W}$ isomorphic to $\mathbb {W}_1$. By [29, Lemma 4.7], it follows that $\mathcal {Y}_{-1}$ and $\mathcal {Z}$ generate a subalgebra of $\mathcal {W}_{\le 1}$ finite codimension. Therefore, $\mathfrak {g}\cap \mathcal {W}_{\le 1}$ has finite codimension in $\mathcal {W}_{\le 1}$.

By a completely symmetric argument, we also get that $\mathfrak {g}\cap \mathbb {W}_1$ has finite codimension in $\mathbb {W}_1$. Therefore, $\mathfrak {g}$ has finite codimension in $\mathcal {W}$. But now Proposition 4.3 implies that

A.1 $$\begin{aligned} g^n\mathcal {W}\subseteq \mathfrak {g}\subseteq g\mathcal {W}\end{aligned}$$

for some $g \in \Bbbk [t,t^{-1}]$, and therefore *g* must divide both $\mathcal {X}_1$ and $\mathcal {X}_2$. Without loss of generality, we may assume that $g \in \Bbbk [t]$, *g* is monic, and g(0)≠0. Note that $\mathcal {X}_1 = -(t^2 - \lambda )\partial$ and $\mathcal {X}_2 = -t^{-1}(t^4 - \mu )\partial$. But $\gcd (t^2 - \lambda , t^4 - \mu ) = 1$, since $\mu \ne \lambda ^2$, and therefore g=1, since *g* is a common divisor of $\mathcal {X}_1$ and $\mathcal {X}_2$. Now (A.1) gives that $\mathfrak {g}= \mathcal {W}$. □

## Footnotes

1 These are known as *induced modules* in the physics literature, with the name “massive modules” reserved for a subset of them. In this paper, we choose to refer to all modules given in Definition 3.6 as massive modules.

2 Not to be confused with our $\mathcal {W}(a,b)$ algebras.

3 In the physics literature, the modules we introduce in this subsection are called *induced modules* and the term “massive modules” only refers to a subset of them. However, we use the term “massive modules” to refer to all such modules instead, as the term “induced modules” would be too vague in our context.

4 However, the procedure by which these representations are contracted seems different to the one introduced in [34].

5 The BMS_3_ algebra was called *W*(2, 2) algebra in this work, with both central elements being equal.
